# Universal Apparent Quantum Yield Model Manifests a Dual Role of Dissolved Organic Matter as Sensitizer and Inhibitor of Methylmercury Photodegradation in Lakes

**DOI:** 10.1021/acs.est.5c18570

**Published:** 2026-06-01

**Authors:** Andrea G. Bravo, Torfi Geir Hilmarsson, Birgit Koehler, Erik Björn, Anders V. Lindfors, Tomas Landelius, Sergi Díez, Stefan Bertilsson, Ulf Skyllberg

**Affiliations:** † Department of Marine Biology and Oceanography, Institut de Ciències del Mar, ICM-CSIC, Barcelona E-08003, Spain; ‡ Department of Ecology and Genetics, Limnology, 8095 University of Uppsala, Uppsala SE-75236, Sweden; § Department of Chemistry, Umeå University, Umeå SE-90187, Sweden; ∥ Finnish Meteorological Institute, Helsinki FI-00101, Finland; ⊥ Swedish Meteorological and Hydrological Institute, Norrköping SE-601 76, Sweden; # Department of Environmental Chemistry, Institute of Environmental Assessment and Water Research, IDAEA-CSIC, Barcelona E-08034, Spain; ∇ Department of Aquatic Sciences and Assessment, Swedish University of Agricultural Sciences, Uppsala SE-75007, Sweden; ○ Department of Forest Ecology and Management, Swedish University of Agricultural Sciences, Umeå SE-90183, Sweden

**Keywords:** Methylmercury, Chromophoric Dissolved Organic Matter, Photodegradation, Lakes

## Abstract

Photodegradation in lakes is a major sink for the toxin methylmercury (MeHg) in forest-wetland-lake ecosystems. Previous attempts to estimate annual rates of MeHg photodegradation in lakes have followed a “black-box approach”, where the process has been related to incident sunlight rather than photon absorption. Here we use experimental data from three contrasting dark and clear boreal lakes to develop the first apparent quantum yield (AQY) model for spectral MeHg photodegradation rates in lakes. The model was proven universal by its ability to predict experimental data from 22 lakes representing five global regions, covering wide ranges in dissolved organic carbon (DOC), specific UV absorbance at 254 nm, and pH (1.8–39.5 mg C L^–1^, 1.7–5.7 L mg^–1^ C m^–1^ and 4.8–8.5, respectively). The AQY model manifests a dual role played by dissolved organic matter (DOM) as a sensitizer, by producing reactive transient species (RTS) upon photon absorbance, and as an inhibitor, by scavenging of RTS by antioxidants associated with aromatic structures in DOM. Using site-specific data on direct and diffuse solar irradiance, MeHg concentrations, and spectral light absorption properties in 1033 lakes, we estimate an annual MeHg photodegradation rate of 12.1 kg y^–1^ in the total volume of Swedish lakes. This value corresponds to 24% of the estimated 51 kg of MeHg that annually is transported with runoff from soil into the same lakes. By the AQY model, we calculate the first regional estimates of MeHg photodegradation in lakes of boreal and temperate Europe, temperate North America, subtropical North America, and tropical South America, providing a basis for the establishment of regional MeHg cycling models.

## Graphical abstract

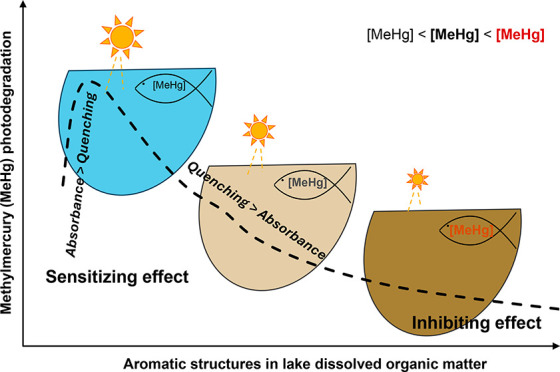

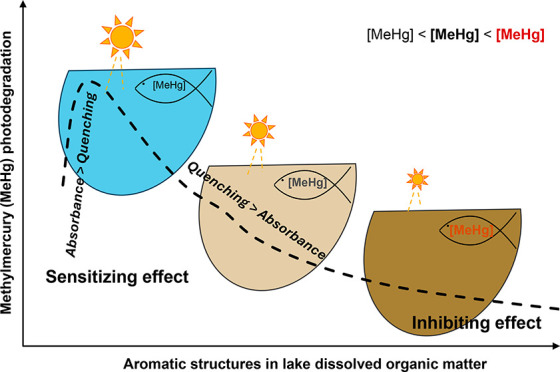

## Introduction

The monomethylmercury (MeHg) molecule, a neurotoxin that is biologically produced from inorganic Hg­(II) in oxygen-deficient soils, sediments and waters,^1–3^ biomagnifies in aquatic food webs and poses health threats to organisms feeding on fish and other aquatic biota.^2^ The concentration level of MeHg in forest-wetland-lake ecosystems is the net result of biotic formation and a multitude of biotic and abiotic degradation processes, where photodegradation in lakes is considered the main sink of MeHg.^4–6^

While it is clearly demonstrated that the MeHg molecule does not undergo direct photolysis by sunlight in absence of dissolved natural organic matter (DOM)^7–10^ stimulatory and inhibitory effects caused by DOM are seemingly complex.^6,10–13^ It is undisputed that the MeHg molecule binds to thiol groups (RS) associated with DOM to form a thermodynamically stable C–Hg–SR-DOM structure.^14^ While the shift in electron density from Hg to S weakens the C–Hg bond,^7,9,10,15–17^ mechanisms involved in the electron transfer to break the bond is under debate. Studies of simple model systems have suggested a direct intramolecular electron transfer involving light-absorbing aromatic structures in direct association to MeHg adsorbing thiol groups,^9,16,17^ while other studies present support for an indirect mechanism where photon absorption by CDOM (chromophoric dissolved organic matter) results in the formation of short-lived, reactive transient species (RTS) with oxidative power to break the C–Hg bond. The most likely RTS candidates occurring in freshwaters with significant DOM concentrations are excited, triplet states of ^3^CDOM* and singlet oxygen (^1^O_2_),^7,8,10,11,18^ where the latter is formed from ^3^CDOM* reaction with O_2._ ^19,20^ By use of chemical scavengers it has been demonstrated that ^1^O_2_,^7,8,13^ ^3^CDOM* ^10,13,21^ and hydroxyl radicals (·OH)^21–24^ all can be involved in MeHg photodegradation in natural water or experimental solutions containing DOM, while superoxide anions (O_2_^•–^) and hydrogen peroxide (H_2_O_2_) are more abundant in wastewater.^25^ It should be noted that the selection of scavengers may be problematic because of their variable specificity and unwanted, secondary effects,^21^ which also influence the possibility to conclude about a direct process for which there is no probe.

The few reported rates of MeHg photodegradation in lakes are either based on empirical relationships relevant only for the studied water body itself,^5,26^ or rely on modeling approaches^4,27–32^ that do not fully consider the theory of aquatic photochemistry.^33–35^ Despite progress to account for the wavelength-dependency of photons involved in the photolysis of MeHg,^29,31,32^ models reported so far have related MeHg degradation to incident rather than absorbed light, which can be considered as a “black-box approach”. These empirical models do not formulate mechanisms that distinguish between stimulatory and inhibitory effects caused by DOM and can therefore not be used for upscaling purposes in other lakes than the ones from which experimental data originate.

To fill this gap, we here present a model that is shown to be applicable to a wide range of lakes representing different global regions. The model builds on the well-established photochemical concept of apparent quantum yield (AQY, mol E^–1^)^34,35^ by relating the number of MeHg molecules degraded to the number of photons absorbed by CDOM.^36–40^ The model quantifies the stimulatory effect by CDOM photon absorbance, through the indirect formation of RTS, as well as the inhibitory effect caused by antioxidants (AO) associated with DOM, acting as quenchers of RTS. Phenolic AO have been demonstrated to scavenge triplet state radical intermediates of organic pollutants, ^3^CDOM* and ^1^O_2_.^18,41–45^ The model is derived from spectral experimental data collected in three lakes featuring contrasting quantities and qualities of DOM and it is validated and optimized against another set of experimental data collected from 22 globally distributed lakes. Taking advantage of existing large-scale optical data sets available for lakes, we present the first large-scaled estimates of MeHg photodegradation in lakes of boreal, temperate, subtropical and tropical regions. These estimates provide a necessary foundation for the construction of MeHg cycling models on regional and global scales. While models for the cycling of Hg(0) and Hg­(II) between land, air and water are numerous, a quantification of processes of MeHg formation and degradation at different scales is currently missing.

## Materials and Methods

### Experimental Determination of Spectral Rates of MeHg Photodegradation in Three Contrasting Lakes

Experiments were conducted in a solar simulator (light spectrum, Figure S1) using water from three Swedish boreal lakes: Ljustjärn (LJU), Lilla Sångaren (LS) and Ängessjön (ANG), contrasting in lake characteristics, chemical data (Table S1) and spectral absorbance (Figure S1). The LJU and ANG water samples represent end-members of clear (DOC concentration: 2.9 mg L^–1^, SUVA_254_: 1.8 L mg^–1^ m^–1^) and dark (DOC concentration: 39 mg L^–1^, SUVA_254_: 3.7 L mg^–1^ m^–1^) lakes, respectively, while LS is intermediate in DOC (9.0 mg L^–1^) and high in SUVA_254nm_ (4.2 L mg^–1^ m^–1^). Experimental data on spectral rates of MeHg photodegradation in lake water (filtered by 0.2 μm membrane filters, Gelman Supor) from LS and ANG were combined to derive an AQY model for intermediate-to-high SUVA and DOC (dark lake model) and data from LJU were used to derive an AQY model for clear lakes. The experimental setup is described in SI Text S1.

To clarify the effect of sample storage, water taken in lake ANG was divided into two samples (ANG1 and ANG2), where ANG1 was immediately used in experiments and ANG2 was kept stored (after 0.2 μm filtration) in darkness at 4 °C for 2 months. No significant effect of storage on MeHg photodegradation was observed (Figure S2).

### Derivation of Spectral AQY Models from Experimental Data Obtained in Lake Water from Dark and Clear Lakes

The MeHg photodegradation process is considered to follow a pseudo-first-order kinetic reaction, as described by eqs S1–S2, SI Text S2. Based on the absorptive properties of CDOM in the three experimental lakes the spectral, absorbed photon flux (*Q*_*abs*(λ)_) was calculated by eq S4 and summarized for a specific wavelength interval. After multiplication with the light exposure time (*t*) pseudo-first-order MeHg photodegradation rate constants, *k*_*pd abs*_ could be determined from the negative slopes of the linear relationships obtained between *Q*_*abs*_ *t* (x) and the term ln­([MeHg]_*t*_/[MeHg]_0_), as described by eq S3. The apparent quantum yield, AQY (Φ) was finally calculated by eq S5.

To explore the wavelength dependency of MeHg photodegradation we followed the methodology of Koehler et al.^40^ and applied bandpass filters (CVI Laser Corporation, former Gamma Optronik AB, Sweden and Oriel Instruments, Newport Corporation, Irvine, California) to cut off the radiation below wavelengths of 250, 309, 350, 380, and 420 nm. Filters were placed on top of the vials used in the solar simulator. Experimental data showed linear relationships between absorbed photons and ln­([MeHg]_*t*_/[MeHg]_0_) with increasing negative slopes with shorter wavelengths passing through the filters (dark lakes ANG12, LS, Figure S2 and the clear lake LJU, Figure S3).

We calculated experimentally determined AQY by eqs S1–S5 for the wavelength intervals defined by the five cutoff filters (Φ_250–700_, Φ_310–700_, Φ_351–700_, Φ_381–700_, and Φ_421–700_) and then iteratively fitted three a priori mathematical functions to these data, a power function, eq 1, a previously used function for this purpose by Vähätalo et al., eq 2 ^37^ and the exponential function used by Johannessen and Miller, eq 3.^46^

1 $$\Phi_{\lambda }=a\lambda^{-b}$$

2 $$\Phi_{\lambda }=a10^{-b\lambda }$$

$$\Phi_{\lambda }=e^{-(m1+m(2\lambda -290))}$$ 3

4 $$\mathrm{Merit‐of‐fit}=\Sigma {\left(\Phi_{\exp}-\Phi_{\mathrm{model}}\right)}^{2}/{\Phi_{\exp}}^{2}$$

Values of the parameters *a, b* and *m* in eqs 1–3 were optimized by first calculating the AQY for each of the wavelength-intervals 250–309, 310–350, 351–380, 381–420 and 421–700 nm, as explained in SI Text S3. These values were in turned multiplied by the corresponding fraction of photon absorption (of the total spectrum 250–700 nm) and summed up to yield modeled AQY for the five different cutoff filters (Φ_250–700_, Φ_310–700_, Φ_351–700_, Φ_381–700_, and Φ_421–700_). Optimization of the models was judged by minimizing the merit-of-fit, eq 4 for the difference between measured and modeled values on Φ_250–700_, Φ_310–700_, Φ_351–700_, Φ_381–700_, and Φ_421–700_, and by increased linearity for the relationship between measured and modeled AQY (Table S2). In this way three optimized spectral AQY models, eqs 5–7, were derived for the combined data set of the three dark water samples ANG12 + LS (Figure S4), and three spectral AQY models, eqs 8–10, were derived for the clear water lake (LJU, Figure S5).

*Spectral AQY models optimized for LS* + *ANG12–dark lakes:*

Power function:

5 $$AQY_{\lambda \;}\left(nmol\;E^{-1}\right)=10^{20.7}\lambda^{-9.0}$$

Vähätalo function:

6 $$AQY_{\lambda \;}\left(nmol\;E^{-1}\right)=61.5\times \left(10^{-0.0113\lambda }\right)$$

Exponential function:

$$AQY_{\lambda \;}\left(nmol\;E^{-1}\right)=e^{-\left(3.12+0.031\left(\lambda -290\right)\right)}$$ 7

*Spectral AQY models optimized for LJU–clear lake:*

Power function:

8 $$AQY_{\lambda \;}\left(nmol\;E^{-1}\right)=10^{15.3}\lambda^{-6.5}$$

Vähätalo function:

9 $$AQY_{\lambda \;}\left(nmol\;E^{-1}\right)=16.7\times \left(10^{-0.007\lambda }\right)$$

Exponential function:

$$AQY_{\lambda \;}\left(nmol\;E^{-1}\right)=e^{-\left(1.71+0.02\left(\lambda -290\right)\right)}$$ 10

To enable comparison with previously published results, we also formulated incidence radiation models for the dark end-member lake ANG1 (Figure S6), as well as for the clear lake LJU (Figure S5), SI Text S4 and eqs S10–S12 and eqs S16–S18, respectively. A spectral AQY model (Figure S6) was also developed for the dark end-member lake ANG1 by eqs S13–S15. The full matrix of functions and data sets resulted in six models for the calculation of *k*_pd(λ)inci_ and nine models for the calculation of AQY_λ_ (Table S3).

### Experimentally Determined Rates of MeHg Photodegradation in 22 Globally Distributed Lakes

To validate and further optimize the AQY models derived for dark and clear lakes, additional MeHg photodegradation experiments were conducted in the solar simulator under a full light spectrum (applying 250 nm filters) in lake water collected from another 22 lakes representing five different regions of the world (Table S4, S5 and Figure S7) covering DOC, *SUVA*_254_ and pH ranges of 1.8–39.5 mg C L^–1^, 1.7–5.7 L mg^–1^ C m^–1^ and 4.8–8.5, respectively. Lake water was immediately after sampling filtered through 0.2 μm membrane filters (Gelman Supor) to minimize microbial MeHg degradation during shipping and storage. Samples were shipped on ice by air and stored in darkness at 4 °C until experiments were conducted within 1 month after sampling to avoid potential confounders caused by long-term storage. The experimental results of MeHg photodegradation for the 22 lakes are reported in Figure S8.

### AQY Model Validation and Optimization by Experimental Data from 22 Globally Distributed Lakes–Inhibitory Effect of DOM-Associated Antioxidants

To account for the inhibitory effect caused by DOM we followed the approach taken to describe quenching of triplet state intermediates (including ^3^CDOM*) by DOM-associated antioxidants (AO).^42,45,47^ Using UV absorbance at 254 nm (UVA) as a proxy for AO associated with electron donation capacity^41^ and aromatic structures of DOM,^48^ it was substituted for AO in the term 1/(1 + *AO/AO_1/2_*),^42,45^ yielding the inhibitory term *a* exp­(*b*/(1 + *UVA/UVA_1/2_*). This term was added to the wavelength-integrated full spectrum AQY *(*ΣΦ_250–700_), obtained by summarizing the spectral *AQY*_λ_ *(*nmol *E* ^*–1*^) for the dark, LS + ANG12 eqs 5–7 and clear lakes, LJU eqs 8–10 over the full wavelength spectrum of 250 to 700 nm. The *AQY_quench_* models were finally optimized by fitting the constants *a* and *b* to the data set of all 25 lakes, minimizing the merit-of-fit (eq 4) where Φ_exp_ is the measured and Φ_model_ the modeled full spectrum AQY (ΣΦ_250–700_), respectively, yielding eqs 11–16.

*Optimized dark lake (LS + ANG12) full spectrum AQY models including quenching term*

Power function:

$$AQY_{quench\;}\left(nmol\;E^{-1}\right)=\Sigma \Phi_{250-700}\times 0.922\;\exp\left(3.89/\left(1+\mathrm{UVA}/{\mathrm{UVA}}_{1/2}\right)\right)$$ 11

Vähätalo function:

$$AQY_{quench\;}\left(nmol\;E^{-1}\right)=\Sigma \Phi_{250-700}\times 0.976\;\exp\left(3.87/\left(1+{\mathrm{UVA}/\mathrm{UVA}}_{1/2}\right)\right)$$ 12

Exponential function:

$$AQY_{quench\;}\left(nmol\;E^{-1}\right)=\Sigma \Phi_{250-700}\times 0.955\;\exp\left(3.89/\left(1+{\mathrm{UVA}/\mathrm{UVA}}_{1/2}\right)\right)$$ 13

*Optimized clear lake (LJU) full spectrum AQY models including quenching term*

Power function:

$$AQY_{quench\;}\left(nmol\;E^{-1}\right)=\Sigma \Phi_{250-700}\times 0.0186\;\exp\left(5.2/\left(1+\mathrm{UVA}/{\mathrm{UVA}}_{1/2}\right)\right)$$ 14

Vähätalo function:

$$AQY_{quench\;}\left(nmol\;E^{-1}\right)=\Sigma \Phi_{250-700}\times 0.0174\;\exp\left(5.25/\left(1+\mathrm{UVA}/{\mathrm{UVA}}_{1/2}\right)\right)$$ 15

Exponential function:

$$AQY_{quench\;}\left(nmol\;E^{-1}\right)=\Sigma \Phi_{250-700}\times 0.02\;\exp\left(5.25/\left(1+\mathrm{UVA}/{\mathrm{UVA}}_{1/2}\right)\right)$$ 16

For the dark lake model UV*A*_1/2_ was set to 20 (m^–1^), which is the median value of all 25 lakes, while a value of 50 (m^–1^) gave a better fit of the clear lake model. Linearity and coefficients of determination (R^2^) of the regressions between measured and modeled AQY by these equations are reported in Table S6. The full spectrum AQY, ΣΦ_250–700_, can be recalculated to the full spectrum photodegradation constant, *k*_*pd abs*_, which is independent of the concentration of MeHg, by multiplying ΣΦ_250–700_ with the quotient 1/([MeHg]*V/A*), eq S5). This quotient differed in experiments with the three Swedish lakes (5.5–8.0) and the 22 globally distributed lakes (average 3.4), due to differences in MeHg concentrations in the experimental setup. Model fits were therefore slightly improved when these experimental differences were accounted for in the final model validation (Figure 1D,E). Two exponential models, one for dark, eq 17, and one for clear, eq 18 lakes, were derived for the calculation of *k*_*pd abs quench*_ by multiplying the factor of 3.725 (the average of 1/([MeHg]*V/A*) obtained for the experiments with all 25 lakes) with the coefficients of 0.955 and 0.02 in eqs 13 and 16, respectively.

**Figure 1:**
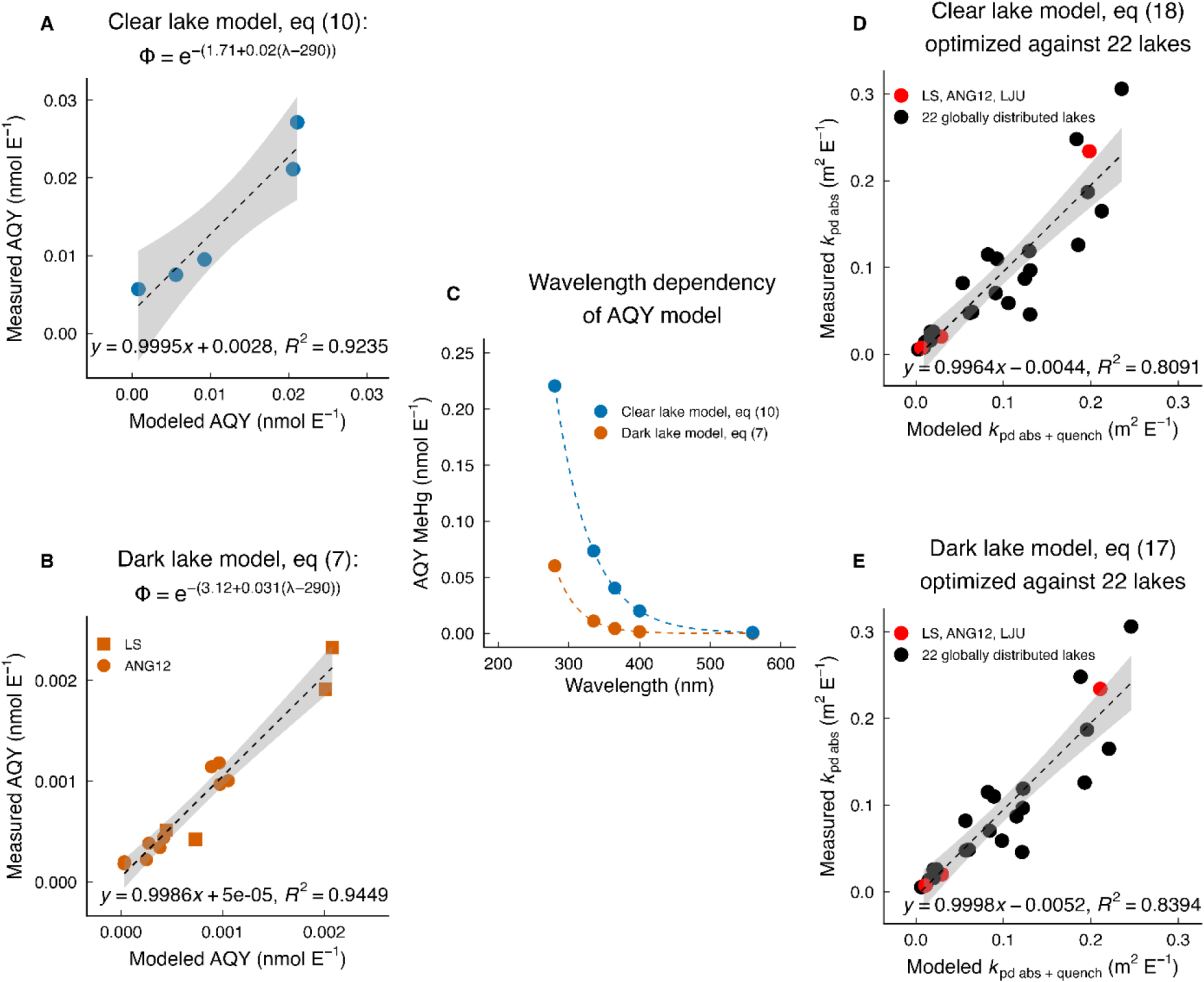
Apparent quantum yield (AQY) model devolopment. Models were derived from experimental data from (A) one clear lake (LJU) and (B) two dark lakes (LS, ANG12). Spectral AQY models for MeHg photodegradation (C) were derived for the clear (eq 10) and dark lakes (eq 7) from data obtained with optical bandpass filters (symbols denote midpoints of 250–309, 310–350, 351–380, 381–420, 421–700 nm intervals). By inclusion of the quenching term *a* exp­(*b*/(1 + *UVA*/*UVA*_1/2_), and optimizing the values *a* and *b* to experimental data from 22 globally distributed lakes could MeHg photodegradation constants (*k_pd abs+quench_*, m^2^ E^–1^) be calculated by eq 17 for dark and eq 18 for clear lakes. The two models (clear lake, D, and dark lake, E) equally well predicted the experimentally determined MeHg photodegradation constant (*k_pd abs_*, m^2^ E^–1^) in the population of all 25 lakes studied. Shading denotes 95% confidence intervals for the regression equations.

*Optimized dark lake (LS + ANG12) full spectrum k_pd abs+quench_ model*

Exponential function:

$$k_{pdabs+quench\;}\left(m^{2}E^{-1}\right)=\Sigma \Phi_{250-700}\times 3.55\;\exp\left(3.89/\left(1+\mathrm{UVA}/{\mathrm{UVA}}_{1/2}\right)\right)$$ 17

*Optimized clear lake (LJU) full spectrum k*_*pd abs+quench*_ *model*

Exponential function:

$$k_{pdabs+quench\;}\left(m^{2}E^{-1}\right)=\Sigma \Phi_{250-700}\times 0.074\;\exp\left(5.25/\left(1+\mathrm{UVA}/{\mathrm{UVA}}_{1/2}\right)\right)$$ 18

### Calculation and Upscaling of MeHg Photodegradation to Lakes in Sweden and Global Regions

The dark and clear lake *AQY*_*quench*_ models were applied to a lake water data set of 1033 Swedish lakes (Table S7), as well as to data sets representative for lakes in boreal, temperate, subtropical and tropical regions (Figure S7), to calculate annual rates of MeHg photodegradation in lakes. At all these sites incoming hourly direct and diffuse spectral solar irradiance was recalculated to down-welling irradiance below the water surface as described in SI Text S5. Spectral AQY, Φ_(λ)_, derived from eqs 5–10 and S13–S15, were together with the data on daily (integrated from hourly) scalar down-welling irradiance below the water surface, *E­(*λ, 0^–^), the spectral CDOM Naperian absorption coefficient (*a_g_*, m^–1^) and molar concentrations of MeHg in lake water used to calculate the daily spectral MeHg photodegradation (ψ_*abs*(λ)_, mol m^–3^ d^–1^) by depth (*z*, m) in the lake water. The spectral degradation of MeHg was integrated between 290 to 700 nm to yield the daily, full spectrum MeHg photodegradation (ψ_abs(z)_, nmol m^–3^ d^–1^) by eq 19.

$$\psi_{\mathrm{abs}\left(z\right)}=\int_{290}^{700}E\left(\lambda ,0^{-}\right)\;a_{g}\left(\lambda \right)e^{-\left(Kd\left(\lambda \right)z\right)}\Phi \left(\lambda \right)d\lambda$$ 19

Because the rate of MeHg photodegradation is first-order in MeHg concentration, this parameter needs to be considered in the equation and Φ­(λ) was therefore substituted for *k*_*pd*(λ)abs_ × [MeHg] × *V/A*, in eq S5. The term *V/A* (m) is the ratio of volume (m^3^) to area (m^2^) of the lake water depth-interval selected, and it can in turn be substituted for the depth-interval Δ*z* (m) to yield eq 20, from which the daily MeHg photodegradation is calculated as mol m^–3^ d^–1^ per depth-interval (Δ*z*).

$$\psi_{\mathrm{abs}\left(\Delta z\right)}=\int_{290}^{700}E\left(\lambda ,0^{-}\right)\;a_{g\;}\left(\lambda \right)e^{-\left(Kd\left(\lambda \right)z\right)}k_{pd\left(\lambda \right)abs}\left[\mathrm{MeHg}\right]\Delta zd\lambda$$ 20

The *k*_*pd*(λ)abs_ in eq 20 is derived from the wavelength-dependent AQY functions, by multiplying Φ_λ_ by the conversion factor 1/([MeHg]*V/A*) of 3.725, which is the average value of the quotient in experiments of the 25 lake waters from which the wavelength-dependent AQY functions and the quenching term were derived. Thus, wavelength-specific values of *k*_*pd*(λ)abs_ were calculated from functions on the form *k*_*pd*(λ)abs_ m^2^ E^–1^ = 3.725 Φ_λ_, where Φ_λ_ was calculated by eqs 5–10.

To account for the light attenuation by depth in lakes, as described by the term *e* ^–(*Kd*(λ)z)^ in eq 20, the degradation of MeHg was calculated for fixed depth-increments of 0.005 m from the surface to the mean water depth. The *K*_*d*_ was calculated from linear regressions established between absorbance and *K*_*d*_ for different wavebands.^40^ The depth-integrated, daily MeHg photodegradation (ψ_abs(z)_, mol m^–2^ d^–1^) was obtained by multiplying the ψ_abs(Δz)_, mol m^–3^ d^–1^, with the depth-increment Δ*z* (m), and summing up all increments from the surface to the mean depth of the lake, eq 21.

$$\psi_{abs\left(z\right)}=\overset{z=mean\;depth}{\underset{z=0}{\sum }}\psi_{\mathrm{abs}\left(\Delta z\right)}\Delta z$$ 21

Daily values on ψ_abs(z)_ (mol m^–2^ d^–1^) were summed up for the ice-free season to yield the annual rate of MeHg photodegradation in the lake (mol m^–2^ y^–1^ or ng m^–2^ y^–1^). Thus, the variability of direct and diffuse light over the day and the year was taken into consideration during upscaling.

To account for the inhibitory effects by DOM, the term *a* exp­(*b*/(1 + *UVA/UVA_1/2_*) was included in the upscaling calculations. Because this term is wavelength-independent, it could either be applied by substituting spectral *k*_*pd*(λ)*abs+quench*_ *for k*_*pd*(λ)abs_ in eq 20 or by multiplication with ψ_abs(Δz)_ after summation over all wavelengths. For comparative purposes we also calculated the upscaled MeHg photodegradation using the incident irradiance model. Values on *k*_pd(λ)inci_ calculated by eqs S10–S12 for ANG1 dark lake water and S16–S18 for LJU clear lake water were substituted for *k*_*pd*(λ)abs_ in eq 20 and the CDOM absorption term a_g_ was omitted. Because the parameter ψ_inci(Δz)_ calculated by eq 20 for each depth-interval has the unit mol m^–2^ d^–1^, ψ_inci(z)_ is calculated by summing up ψ_inci(Δz)_ for all depth layers.

## Results and Discussion

### Apparent Quantum Yield Models Developed for Clear and Dark Lakes

Experimental data from lake LJU, (Figure 1A and S5) and the three water samples ANG12 + LS, (Figures 1B and S4) were used to derive clear and dark lake AQY models, respectively. Because experimental data from ANG1 and ANG2 were not significantly different (Figure S4), storage effects were small and these data could be combined to derive the dark lake AQY model. Spectral AQY, Φ_λ_, functions, defined as moles of MeHg degraded per moles of absorbed photons (nmol E^–1^), were fitted to experimental data. Of the three a priory functions tested, eqs 1–3, the wavelength dependency of AQY was best explained by the exponential function Φ_λ_ = e^–(*m1*+*m2*(λ–290))^ in the three dark lake waters (LS + ANG12), as judged by the merit-of-fit and by linearity of relation between modeled and measured AQY (Figure S4 and Table S2), while fits of the three functions to the clear lake data can be regarded equal. The exponential AQY models, eqs 7 and 10 were used for further validation and optimization of the AQY model for dark and clear lakes, respectively.

### Derivation of Universal AQY Model

A comparison of the AQY calculated at specific wavelengths demonstrates that MeHg is degraded more efficiently in the clear than in the dark lakes (Figure 1C). To further explore this difference, the two AQY models were evaluated by experimental data from 22 globally distributed lakes (Figure S7, Table S4 and S5). The dark and clear lake models, eqs 7 and 10 respectively, integrated to the full irradiance spectrum (AQY, ΣΦ_250–700*model*_), demonstrated significant (R^2^ > 0.78) positive, exponential relationships with the experimentally determined ΣΦ_250–700*exp*_ in the data set of all 25 lakes (Figure S9a). As demonstrated by the proximity between the red symbols (lakes LJU, LS, ANG12) and the 1:1 line, the clear lake model reasonably well predicted the MeHg photodegradation measured in lake LJU, and the dark lake model reasonably well predicted the MeHg photodegradation in the lakes LS and ANG12. However, for the other 22 lakes, the prediction accuracy of the two models was poor. Thus, while the significant, positive relationship between predicted ΣΦ_250–700*model*_ and measured ΣΦ_250–700*exp*_ suggests that light absorbance by CDOM indeed is important, additional mechanisms are obviously involved in the MeHg photodegradation process.

The prediction error of the two models, calculated as the discrepancy between the experimentally measured ΣΦ_250–700*exp*_ and modeled ΣΦ_250–700*model*_, was significantly, negatively correlated with *SUVA*_254_. The dark lake model systematically underestimated the MeHg photodegradation in lakes having a *SUVA*_254_ lower than the LS + ANG12 lakes (Figure S9b), while the clear lake model underestimated (positive numbers) or overestimated (negative numbers) the MeHg photodegradation in lakes having a *SUVA*_254_ lower or higher than the LJU lake, respectively (Figure S10a). The exponential relationships (Figure S9a) could be understood as if only a relatively small fraction of the photons absorbed by CDOM is giving rise to MeHg photodegradation. Further, the systematic model prediction errors as related to *SUVA*_254_ (Figures S9b and S10a) indicate an inhibitory effect, not captured by the AQY model, as related to UV light absorbance.

It is well-established that *SUVA*_254_ is a reasonable proxy for the concentration of aromatic structures associated with DOM.^48^ Aromatic structures, such as phenols, are major antioxidants (AO) with an ability to scavenge (quench) ^3^CDOM* and ^1^O_2_.^41,44,49,50^ We therefore interpret the systematic deviations between modeled and measured MeHg photodegradation as a result of RTS quenching by DOM-associated AO. To quantitatively account for this effect in our model, we followed an approach taken in previous work using the term 1/(1 + *AO/AO*_1/2_), where 1/2 denotes half of the inhibitory effect caused by AO, as a proxy for RTS quenching in connection to DOM-sensitized degradation of organic pollutants.^45^

In previous research AO has been approximated by phenols,^42^ the electron donation capacity (EDC) of DOM,^45^ or simply by DOC.^25^ Given the observed relationships between the AQY model prediction error and *SUVA_254_* we used UV absorbance at 254 nm (*UVA*) as a proxy for AO and incorporated the term 1/*(*1 + *UVA/UVA*_1/2_) into our model. In support for this approach the parameter 1/(1 + *UVA/UVA*_1/2_), plotted against the residuals of the AQY model, demonstrated a significant positive, exponential relationship for the dark lake model (Figure S9c). A similar pattern was observed also for the clear lake model, where negative numbers precluded fitting of an exponential function (Figure S10b). In the final optimization process the term *a* exp­(*b/(*1 + *UVA/UVA*_1/2_)) was fitted to data from the 22 globally distributed lakes, yielding the final *AQY*_*quench*_ models, eqs 11–13 and eqs 14–16 for dark and clear lakes, respectively.

The *UVA*_1/2_ was optimized to a value of 20 for the dark lake model and 50 for the clear lake model. Because the parameters *a* and *b* are freely fitted, the value on *UVA*_1/2_ can be regarded as reflecting relative differences in MeHg photolysis inhibition by DOM rather than reflecting the absolute concentration of AO in DOM. In six lakes with concentrations of Fe exceeding the detection limit (Table S5), UVA was corrected for Fe absorbance at 254 nm following the procedure of Weishaar et al.,^48^ while concentrations of nitrate (absorbing at 254 nm) with exception of Funil reservoir (Table S5) were generally low. Thus, *UVA_254_*, which is a parameter available for many lakes, is expected to be reflecting aromaticity and used as a proxy for AO in the data sets we use.

The two optimized *AQY*_*quench*_ models (dark and clear) equally well predicted the experimentally determined full spectrum *AQY* (ΣΦ_250–700_, Figure S11) and recalculated to the corresponding pseudo–first-order rate constant, *k*_*pd abs+quench*_, eqs 17 and 18 equally well predicted the experimentally determined *k*_*pd abs*_, m^2^ E^–1^ (Figure 1D,E) in the full data set of 25 lakes. The fact that the two models, derived from very different types of lakes (dark: LS + ANG12 and clear: LJU), equally well predicted the MeHg photodegradation in lakes from five regions of the world suggests the *AQY_quench_* model is universally applicable.

### Robustness of the *AQY_quench_* Model: Upscaling of MeHg Photodegradation to Swedish Lakes

To evaluate the robustness of the dark and clear *AQY*_*quench*_ models they were applied to a data set of 1033 Swedish lakes^51^ covering wide ranges in DOC (1.9–50 mg L^–1^), SUVA_254_ (0.6–5.0) and pH (4.2–8.3, Table S7). The full solar spectrum AQY was calculated by eqs 11–13 for the dark lake model and eqs 14–16 for the clear lake model. These data were integrated into annual rates of MeHg photodegradation in each lake by eqs 20 and 21. For this calculation data were required for six major input parameters, (i) lake-specific daily integrated direct and diffuse spectral solar irradiance, corrected for processes at the passage of the water surface to yield below water surface downwelling scalar irradiance (SI Text S5),^40^ (ii) spectral Naperian absorption coefficient (*a*_*g(λ)*_, m^–1^) of CDOM,^37^ (iii) spectral attenuation coefficient in water (*K*_*d(λ)*_, m^–1^),^40^ (iv) water concentrations of MeHg (ng L^–1^) calculated from linear relationships with DOC (Figure S12a), (v) the inhibition term 1/(1 + *UVA/UVA*_1/2_) and (vi) the number of ice-free days and the mean water-depth in the lake.^40^

The average, annual MeHg photodegradation (±SE) in Swedish lakes was 326 ± 4.5 ng m^–2^ y^–1^ and 293 ± 3.4 ng m^–2^ y^–1^, as calculated by the clear and dark lake models, respectively (Table 1). Considering the prediction errors of the two models when applied to the data set of 25 globally distributed lakes (95% confidence intervals in Figure 1D,E) these two estimates are not significantly different.

**Table 1:** Annual Rates of MeHg Photodegradation in Swedish Lakes^a^

MeHg photodegradation	Clear lake model eq 16	Dark lake model eq 13	Combined clear and dark lake model eq 13 + 16	Combined clear and dark lake model eq 12 + 15	Combined clear and dark lake model eq 11 + 14
Median, ng m^–2^ y^–1^	288	297	**302**	340	370
Average, ng m^–2^ y^–1^	326 ± 4.5	293 ± 3.4	**317 ± 4.5**	360 ± 5.2	386 ± 5.4
kg y^–1^ Swedish lake area^–1^	11.5 ± 0.2	11.9 ± 0.2	**12.1 ± 0.2**	13.6 ± 0.2	14.8 ± 0.2

a Median and average (ng m^–2^ y^–1^) ±SE, calculated for 1033 Swedish lakes and upscaled to the Swedish lake area of 39952 km^2^ (kg y^–1^). We used two approaches to fit the models: (*i*) dark and clear lake models were applied separately to the complete data set (Columns 2 and 3), and (ii) the dark lake model was applied to lakes with *SUVA_254_* ≥ 2.5 and the clear model was applied to lakes with *SUVA_254_* < 2.5, as denoted “Combined clear and dark lake model”. The finally selected model (Column 4, bold text) includes the exponential wavelength-dependency function eq 3, which gave a slightly better merit-of-fit than the corresponding power, eq 1, and Vähätalo functions, eq 2, as reported in Table S6. The *AQY*_*quench*_ (nmol *E* ^–1^) calculated by eqs 11–16 was integrated to the average depth of each of the 1033 lakes by eqs 20 and 21

By sorting the 1033 Swedish lakes into DOC classes it is demonstrated that the clear and dark lake *AQY*_*quench*_ model outputs show some systematic differences (Figure 2). According to the clear lake model, Swedish lakes show an optimum of MeHg photodegradation in the DOC class 8–11 mg L^–1^, while the dark lake model predict a plateau of photodegradation above 8–11 mg L^–1^. There are three major parameters driving differences in the outcome of the two models, i) MeHg concentration, ii) photon absorbance by CDOM and iii) the inhibitory factor *a* exp*(b/(*1 *+* *UVA/UVA*_1/2_)).

**Figure 2:**
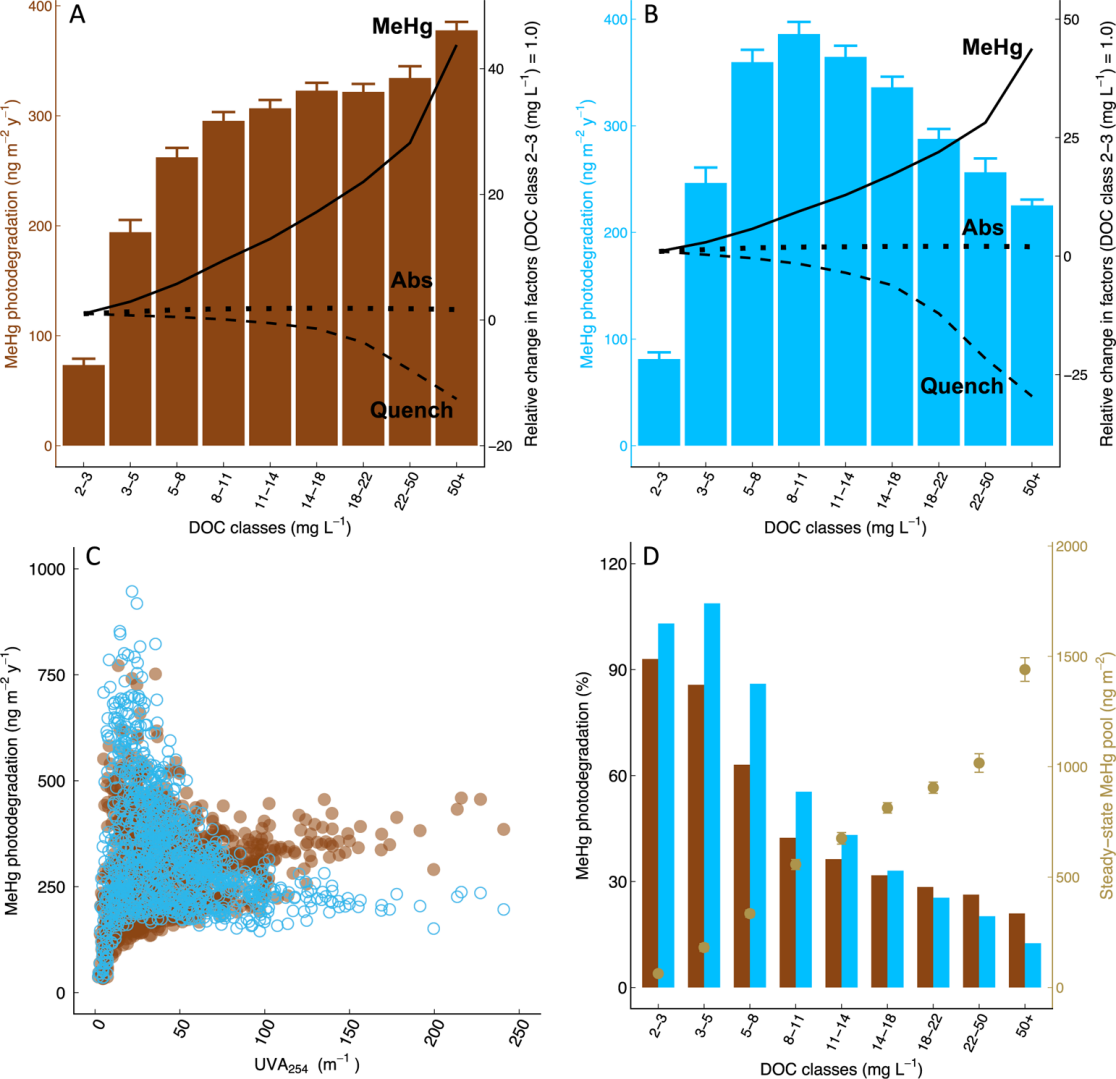
MeHg photodegradation in 1033 Swedish lakes. Results from the dark lake *AQY_quench_* model (A) and the clear lake *AQY_quench_* model (B) sorted into DOC concentration classes. AQY were calculated by the dark lake model by eqs 7 and 13 and by the clear lake model by eqs 10 and 16. Spectral data were integrated to the full solar spectrum and upscaled to whole water bodies by eqs 20 and 21 to yield annual rates of MeHg photodegradation. Absorbance and DOC data were collected in August 2009 ^51^ and direct and diffuse solar radiation during the ice-free season was simulated for the year 2009.^40^ The three lines (right *y*-axis) illustrate the relative change in each of the three factors driving MeHg photodegradation with the DOC class 2–3 mg L^–1^ set to unity. i) MeHg concentration (solid line), ii) photon absorbance by CDOM (dotted line, varying by a factor of ∼2), and iii) quenching reactions of RTS, hatched line, as approximated by the term *a* exp­(*b*/(1 + *UVA*/*UVA*_1/2_). The negative numbers set for quenching demonstrates its inhibitory effect on MeHg photodegradation. Predicted MeHg degradation in relation to *UVA*_254_ (C), and MeHg degradation in % (left axis) of the steady-state MeHg lake pool (right axis) (D) illustrate small but systematic differences between the clear and dark lake models. Error bars denote standard error of the mean for each DOC class.

Because MeHg concentrations were calculated from a linear relationship with DOC, its relative influence increases by a factor of 44 from the lowest to highest DOC class in both models. Neither does CDOM absorption of photons (varying by a factor ∼ 2 among DOC classes) differ significantly between the two models (Figure 2). It is therefore mainly differences in the quenching term *a* exp*(b/(*1 + *UVA/UVA*_1/2_) that is giving rise to differences between the two models, increasing by a factor of 32 between the lowest and highest DOC classes in the clear lake model and by a factor of 15 in the dark lake model. This suggests that Swedish lakes on average have DOM properties with an inhibitory effect on the MeHg photodegradation more in agreement with the dark ANG12 + LS lakes than the clear lake LJU. As noted by Figure 2C, the clear lake model systematically predicted higher MeHg photodegradation in low DOC lakes (*UVA*_254_ < 50 m^–1^) and lower MeHg photodegradation in high DOC lakes (*UVA*_254_ > 100 m^–1^), as compared to the dark lake model. Expressed in relation to the calculated “steady-state MeHg pool” in Swedish lakes, the clear lake model predicted roughly a 10–20% higher degradation in DOC classes < 14 mg L^–1^, and the dark model predicted about 5–10% higher degradation in lakes with >18 mg L^–1^ of DOC (Figure 2D). In SI Text S6 there is some further clarification of differences between the two models.

Following the principle of selecting models in which the RTS quenching properties of DOM are most similar to lakes by which the models were derived, we applied the dark lake model to lakes with *SUVA*_*254*_ ≥ 2.5 (n = 798), and the clear lake model to lakes with *SUVA*_*254*_ < 2.5 (n = 235). By this approach, our final estimate of the MeHg photodegradation in the 1033 Swedish lakes amounts to 317 ± 4.5 ng m^–2^ y^–1^ (mean ± SE, Table 1). To our knowledge, this is the first estimate of MeHg photodegradation covering a region of the world, and it may be considered reasonably representative for southern and northern boreal regions of Europe at latitudes of 55°–68°N.

Because MeHg photodegradation is a key process in the overall cycling of MeHg among soils, waters and the atmosphere, the *AQY*_*quench*_ model is a useful tool by which the first steps can be taken to establish MeHg cycling budgets for freshwater ecosystems of different regions of the world. Such budgets are currently missing, which in turn limits further advancement of scientific understanding of mercury cycling processes, hampering potential actions made by society to mitigate harmful effects of MeHg on humans and wildlife. To further demonstrate the usefulness of the *AQY_quench_* model we can compare our upscaled annual MeHg photodegradation of 12.1 ± 0.2 kg y^–1^ in Swedish lakes (Table 1) with a previously estimated import of 19 and 32 kg y^–1^ MeHg to Swedish lakes from wetland and forest soils, respectively, for the same time period, 2007–2012.^3^ Thus, of the total annual MeHg import of about 51 kg to Swedish lakes from wetlands and forest soils, about 24% is predicted by the AQY model to be photodegraded in the water column. To our knowledge, this is the first robust, regional estimate of the relative importance of annual MeHg input and photodegradation in lakes of forest-wetland-freshwater ecosystems. Previous estimates of MeHg photodegradation in relation to input are limited to a few individual lakes, spanning 27%–106% of the estimated input^5,26,32^ (Table S9).

### MeHg Photodegradation in Lakes of Boreal, Temperate, Subtropical and Tropical Regions

We used the two *AQY*_*quench*_ models to provide the first estimates of MeHg photodegradation in lakes of five global regions (Figure 3 and Table S8). Compared to boreal lakes of Sweden, photodegradation of MeHg is estimated to be roughly two and a half times higher in lakes of temperate Europe (UK lakes, mean ± SD, 800 ± 74 ng m^–2^ y^–1^), four times higher in lakes of temperate N America (Wisconsin, 1180 ± 45 ng m^–2^ y^–1^), five times higher in subtropical N America (Florida Everglades, 1510 ± 35 ng m^–2^ y^–1^), and almost ten times higher in lakes of tropical S America (Brazil 2820 ± 520 ng m^–2^ y^–1^). Comparison of these estimates with model calculations using our globally distributed lakes sorted into smaller groups to cover in principle the same global regions (black bars Figure 3 and Table S8), reveal a very similar pattern, pointing at the robustness of these regional estimates.

**Figure 3:**
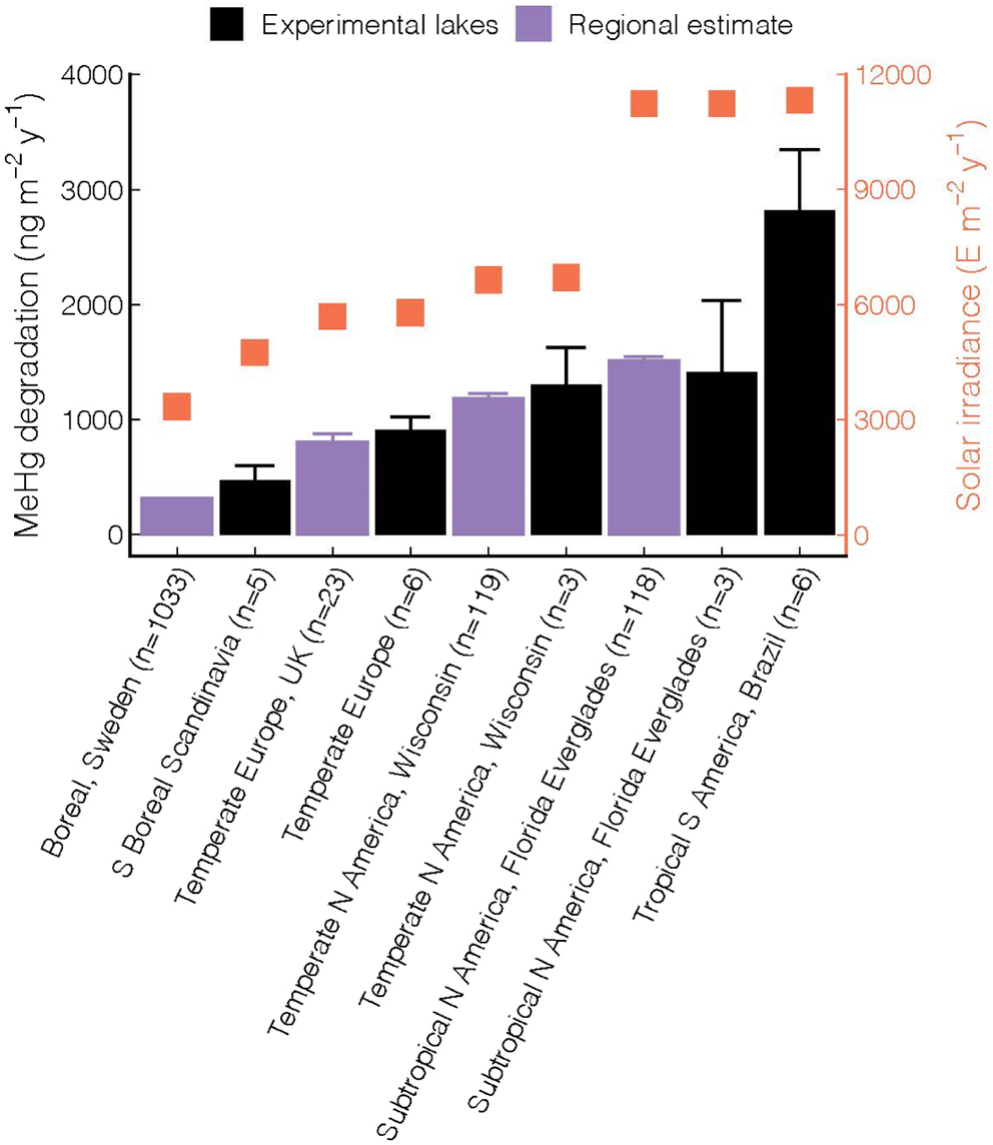
Lake photodegradation of MeHg in different global regions. We used the dark lake model, eq 13, to calculate MeHg photodegradation in lakes with *SUVA*_254_ ≥ 2.0 and the clear lake model, eq 16 in lakes with *SUVA*_254_ < 2.0. Results were integrated to the average depth of lakes by eqs 20 and 21. *Violet bars* represent data sets for which the required lake absorbance data are available to represent four global regions (Figure S7, Table S8, S12). *Black bars* represent experimental data from 23 of the 25 globally distributed lakes (Table S4), averaged and sorted into five regions (Table S8). Error bars denote standard errors of mean. Orange boxes (secondary *y*-axis) illustrate annual, average solar irradiance above ice-free lake water simulated for each of the regions based on hourly spectral (290–600 nm) satellite data, representing the year of 2009 for the 1033 Swedish lakes^40^ and the time period 2005–2016 for the other regions.

The increase in MeHg photodegradation at lower latitude is largely explained by differences in cumulative solar irradiance (driven by solar zenith angle and number of ice-free days). Inhibitory effects, as interpreted to be caused by RTS quenching reactions, are important as well, as demonstrated by *UVA*_254_ (m^–1^) being 2.6 and 3.7 times higher in the Swedish lakes (41 m^–1^), than in lakes of temperate N America (16 m^–1^) and tropical S America (11 m^–1^), respectively. Even if the rate of photodegradation is proportional to MeHg concentrations (as derived from relationships with DOC), the relatively small variability in the latter (ranging from 0.21 ng L^–1^ in tropical S America to 0.39 ng L^–1^ in subtropical N America) has a limited influence on regional differences. Notably in the Florida Everglades, the very shallow waters (averaging 0.4 m water depth) and relatively high *UVA*_254_ (41 m^-1^) resulted in low MeHg degradation rates, despite levels of solar irradiance similar to tropical S America.

### Comparison of Model Results with *In Situ* Measurements of MeHg Photodegradation

It would be highly desirable if the regional estimates of MeHg photodegradation reported here could be validated by independent *in situ* measurements in lakes. Unfortunately, such data are very scarce. The only *in situ* measure of MeHg photodegradation in a boreal lake we are aware of is from lake L240 in Canada^5^ (195 ng m^–2^ y^–1^, 191 ice-free days, Table S9). Another estimate from a boreal lake in southern Norway (298 ng m^–2^ y^–1^, 197 ice-free days, Table S9)^32^ was derived from incident radiation calculations, but it may still be reasonable given the very thin reaction cells used (0.5 cm, resulting in an insignificant “inner-filter-effect”). These estimates may therefore be compared to our calculated annual MeHg photodegradation of 317 ng m^–2^ y^–1^ in Swedish lakes (232 ice-free days). For temperate regions the photodegradation is expected to be higher than in boreal regions due to longer ice-free periods and an average smaller solar zenith angle. The only reported *in situ* measure for a temperate lake is 730 ng m^–2^ y^–1^ in Spring Lake, Minnesota (Table S9).^26^ This value is quite similar to our model calculations of 800 and 910 ng m^–2^ y^–1^ for UK lakes (n = 23) and German and Irish lakes (n = 6), respectively, whereas the average of 1180 ng m^–2^ y^–1^ modeled for 119 Wisconsin temperate lakes is somewhat higher (Table S8). There are to our knowledge no reports on *in situ* measures of MeHg photodegradation in lakes of subtropic or tropic regions of the world.

As illustrated by Figure S14, photon absorption of UV-A (310–399 nm) is largely responsible for the MeHg photodegradation in Swedish boreal lakes (mean ± SE, 63 ± 7%,) and 95% of the photodegradation takes place within the upper 50 cm water depth. These results obtained by the *AQY*_*quench*_ models are in qualitative agreement with previous reports demonstrating the importance of UV-A absorbance for MeHg photodegradation in experiments with DOM, either using different light sources,^10,15^ or film/filters blocking UV–B and UV-A light.^29,31,32^

### Comparison of Results Obtained by AQY and Incident Photons Models

Previously reported models for MeHg photodegradation are based on incident rather than absorbed photons,^4,27–32^ meaning the experimental system is treated as a “black-box” without considering mechanisms behind MeHg photodegradation. To enable comparison with previous reports, we formulated wavelength-dependent incidence radiation models for dark lake ANG1 (eq S12) and clear lake LJU (eq S18) and applied the models to data from the two lakes. While the incidence radiation model inflates the importance of PAR in relation to the UV-A region in both dark and clear lakes, as compared to the AQY model (Figure S15), the two models give reasonably similar estimates of MeHg photodegradation rates in the dark lake, especially if the depth-integration interval is kept small (Figure S16). Thus, data based on “thick experimental solutions” (=high DOM concentration), in which essentially all of the UV-light is absorbed (c.f. ANG lakes in Figure S1), yield estimates by incidence radiation models that may result in reasonable estimates of depth-integrated MeHg photodegradation (despite a biased contribution from different wavelengths), if applied to the same type of lake water as used in the experiments. In contrast, incidence radiation models largely overestimate the MeHg degradation in clear lakes, i.e., “thin experimental solutions” (Figures S15, S16 and SI Text S7). This may be explained by a lack of consideration of the “inner-filter-effect” in incidence photon flux experiments,^15,35^ resulting in incorrect results when integrating biased wavelength contributions by depth in the lake.

### Mechanisms Behind MeHg Photodegradation

Even if stimulatory and inhibitory effects related to DOM have been reported in many studies of MeHg photodegradation, these processes have previously not been mechanistically formulated and unified in one model.^6,10–12,59^ Experimental additions of specific scavengers indicate multiple pathways behind lake MeHg photodegradation,^11,13,21^ where a cocktail of RTS dominated by ^3^DOM*, ^1^O_2_, and ·OH all contribute.^7,8,10,13,21–24^ In lack of specific probes or measures, a direct intramolecular e-transfer process, as demonstrated in simple model systems,^9,16,17^ may be difficult to quantify in natural waters. Support for the process is limited to negative effects on MeHg photodegradation in the presence of known RTS scavengers,^9,21^ which may overestimate its significance.

Even though the primary goal of our study was not to identify molecular scale mechanisms for MeHg photodegradation, the universality of the *AQY*_*quench*_ model clearly manifests a dual role played by DOM in the process, as represented by light absorbance and inhibitory effects. Based on previous research, reaction pathways and a potential cocktail of RTS are expected to vary substantially among the 25 experimental lake waters of this study. The fact that this diversity is reasonably well captured by the quenching term *a* exp*(b/(*1 + *UVA/UVA*_1/2_) in the model suggest photon absorption by CDOM and subsequent quenching of ^3^CDOM* (and secondary RTSs formed from ^3^CDOM*, such as e.g., ^1^O_2_) by antioxidants associated with DOM are key processes behind MeHg photodegradation in many lakes. Notably, similar types of quenching terms, using proxies for phenolic compounds in DOM such as electron-donors and aromaticity have been successfully used to represent the scavenging of triplet state intermediates of organic pollutants^42,44^ and carbon nanomaterials,^52^ as well as of ^3^CDOM* and ^1^O_2_.^45,49,50,62^

If direct, intramolecular e-transfer is contributing significantly to MeHg photodegradation, the quenching term incorporated in the *AQY*_*quench*_ model may represent light absorbance by aromatic keto functionalities not in direct association to the MeHg-SR-DOM molecule, and thus not giving rise to direct MeHg photodegradation. Clearly, the *AQY*_*quench*_ model cannot be used to resolve the relative importance of direct and indirect e-transfer, but nevertheless its ability to handle the variability in MeHg photodegradation in lake waters from different regions of the world, covering wide ranges of environmental factors suggests a universal control of MeHg photolysis by DOM^15^ where MeHg binding to thiol groups, light absorbance by CDOM and inhibitory effects associated with antioxidants are key factors.

### Environmental Implications: DOM in Dark Lakes Protects MeHg from Photodegradation

After the discovery, more than five decades ago,^53^ that the neurotoxin MeHg is produced in natural environments, regions with dark-colored (low pH, high DOC and *SUVA*_254_) lakes have been considered to be at particular risk, demonstrating high rates of MeHg bioaccumulation in aquatic and terrestrial food-webs.^60^ The complex and heterogenic nature of DOM has been emphasized to be in control of the high MeHg concentration levels encountered in these environments, e.g., by serving as energy substrate,^54^ as a regulator of chemical speciation^55^ and in controlling the kinetics of Hg uptake in methylating organisms.^56^ Terrestrial DOM also serves as an important agent for the transport of Hg and MeHg from soils to lakes^57^ and is suggested to be in control of the uptake rate of MeHg at the base of the food-web.^58^

While inhibitory effects exerted by DOM on MeHg photodegradation has been indicated before,^12,59^ the successful quantitative formulation of a RTS quenching term in the *AQY_quench_* model manifests the importance of DOM as an inhibitor of MeHg photolysis in lakes. The inhibitory term varied by a factor of 16 (dark lake model) and 45 (clear lake model), among the 25 globally distributed lakes (dividing the max with min values of ranges reported in Table S6), and by a factor of 15 (dark lake model) and 32 (clear lake model) among the DOC classes of 1033 the Swedish lakes (Figure 2). The much smaller variability in CDOM light absorbance (factor ∼ 2, Figure 2) suggests that, while photon absorption by CDOM is required for the energy transfer to break the Hg–C bond, the concentration of DOM associated antioxidants capable to scavenge RTS is the major factor limiting MeHg photodegradation in many lakes. Put in other words, DOM-associated antioxidants essentially protect MeHg from photolysis in regions with dark, humic lakes, while in clear lakes MeHg is degraded more efficiently. Given that dark lakes in general receive high amounts of MeHg bonded to DOM thiol groups in runoff from wetlands and temporarily water-saturated forest soils,^3,55^ high-DOM-lakes would sustain high levels of MeHg in its water and food-webs. The inhibitory effects of DOM on MeHg photodegradation manifested by the *AQY*_*quench*_ model further strengthen arguments for adjusting land-use practices to counteract the current climate-driven increased browning of surface waters by DOM at northern latitudes.^61^

## Supplementary Material



## Notes

Text on fundamental kinetic modeling and derivation of MeHg photodegradation models; Supporting TEXT S1–S7; Supporting Figures S1–S18; Supporting Tables S1–S12 (PDF)

## ¶

Department of Aquatic Resources, Institute of Coastal Research, Swedish University of Agricultural Sciences, SE-756 51 Uppsala, Sweden
