# Supplementary material for: Universal Apparent Quantum Yield Model Manifests a Dual Role of Dissolved Organic Matter as Sensitizer and Inhibitor of Methylmercury Photodegradation in Lakes
Source: Environ Sci Technol. 2026 Jun 1;60(23):16607–17. doi: 10.1021/acs.est.5c18570 (PMC13276896; doi:10.1021/acs.est.5c18570)
Supplement: Supplementary file 1 [file es5c18570_si_001.pdf]

SUPPORTING INFORMATION

Universal apparent quantum yield model manifests a dual role of dissolved organic matter as sensitizer and inhibitor of methylmercury photodegradation in lakes

Andrea G. Bravo, Torfi Geir Hilmarsson, Birgit Koehler, Erik Björn, Anders V. Lindfors, Tomas Landelius, Sergi Díez, Stefan Bertilsson, Ulf Skyllberg*

Supporting Text for the Materials and Methods Section

SI TEXT S1. Experimental set-up and determination of rates of MeHg photodegradation in a solar simulator

Water samples were exposed to artificial solar radiation using a solar simulator (Q-Sun 1000 Xenon test chamber, Q-panel Lab Products Europe, Bolton, UK) set to 0.59 W m^{-2} at 340 nm (calibrated with the instrument's CR20 Calibration Radiometer). The raw spectrum without applied cut-off filter, and spectra after passing 250, 309, 350, 380 and 420 nm filters are illustrated in **Fig. S1**. It can be noted that Lake water samples were irradiated in duplicate for 3, 6, 12, 18 and 30 hours in 50 mL cylindrical glass vessels with planar quartz top and bottom, painted black and taped on sides and bottom to avoid radiation entering from other directions than from the top. Dark controls (wrapped in Al-foil) were measured at the same time intervals. A cooled water bath maintained the temperature at 25°± 1° C. Thin needles were inserted through the septa covering one of the vial outlets to enable pressure release during radiation exposure. Prior to experiments, reaction vials were soaked in 10 % HNO_3 for at least ten hours, followed by washing with Milli-Q water. Individual dimensions and volumes were determined for each vessel and used in calculations. Individual transmission spectra were determined for each cut-off filter using a Lambda 40 spectrophotometer (PerkinElmer Life and Analytical Sciences). Individual vials and cut-off filters were put at the same positions in the solar simulator in each experiment. Irradiance spectra (250–700 nm) were measured at each of these positions on 10 occasions throughout the study period, using a spectrometer (BLACK Comet UV-VIS, StellarNet Inc., Tampa, Florida, USA) equipped with a fibre optic cable (STEF600-UVVis-SR, StellarNet) and a cosine receptor for UV-Vis near-infrared irradiance (STE-CR2, StellarNet). Nitrite ultraviolet actinometry was employed to validate calculations of photons absorbed by CDOM for the specific irradiation setup.[1,2] Absorbed photons, determined with the spectrally

resolved calculation for AQY determination and broadband actinometry, differed by a factor of 1.43 ± 0.04 under the complete irradiation spectrum in the solar simulator, which is on the same level as in laboratories employing a similar methodology to determine CDOM-absorbed photons.[3]

Water samples were spiked with ^{200}MeHg isotopically enriched standard (synthesized in-house from ^{200}Hg(II) (Oak Ridge National Laboratory, batch 185091)[4] to yield an approximate concentration of 1 ng L^{-1} before exposure in the solar simulator. Immediately after light exposure, ^{204}MeHg was added as an internal standard to the water samples. The standard was allowed to equilibrate for 30 min before preservation of samples by freezing at -20 °C until analysis. Concentrations of MeHg were determined by isotope dilution analysis using thermal desorption gas chromatography inductively coupled plasma mass spectrometry (TDGC-ICPMS, Markes-100, Agilent 6890, Agilent 7700), after ethylation of MeHg by sodium tetraethyl borate at pH 4.9, purged and trapped on Tenax adsorbent tubes[5] and thermally desorbed to a thermal desorption gas chromatography inductively coupled plasma. The detection limit of MeHg was 0.012 ng L^{-1}, corresponding to the mean of concentrations of the method blanks plus three times the standard deviation of the blanks. Experiments for all the 25 globally distributed lakes were conducted under full solar spectrum, without applying any wavelength cut-off filters, and the experimental data are illustrated in **Fig. S11**.

SI TEXT S2. Fundamentals in kinetic modelling: Calculation of first-order MeHg photodegradation constants and apparent quantum yield.

By its simplest description, MeHg photolysis can be described by a second-order process, first-order in the quantity of RTS and first-order in the concentration of reactive forms of MeHg. If light conditions and concentrations of RTS are considered constant throughout the experiment, a pseudo first-order reaction can describe the process, equation (S1).

$$-d[MeHg]/dt = k[MeHg] \quad \text{(S1)}$$

The integrated form yields equation (S2),

$$\ln([MeHg]_t/[MeHg]_0) = -kt \quad \text{(S2)}$$

where the rate constant per days, k (d^{-1}), can be experimentally determined by establishing a linear relationship between $\ln([MeHg]_t/[MeHg]_0)$ and time (t). Thus, a pseudo-first-order reaction is expected if light conditions and concentrations of photon-absorbing components are kept constant throughout the experiment. Yet, the ultimate factor behind MeHg photodegradation is not time *per se*, but rather the photon flux density Q (mol m^{-2} s^{-1}). If time in eqs. (S1) and (S2) is multiplied with Q, the photodegradation constant k_{pd} is determined by the linear relationship between $\ln([MeHg]_t/[MeHg]_0)$ and the density of cumulative moles of photons (Qt, E m^{-2}).

$$\ln([MeHg]_t/[MeHg]_0) = -k_{pd} \times Qt \quad \text{(S3)}$$

The constant k_{pd} has the unit of (m^2 E^{-1}), and it may be calculated for the flux of incident photons, $k_{\text{pd inci}}$, after correction for physical processes when passing the water surface, or the flux of photons absorbed by components in the water sample, $k_{\text{pd abs}}$. The incident irradiance (I, W m^{-2}) is transformed to incident photon flux ($Q_{inci\,(\lambda)}$ µmol m^{-2} s^{-1}) by multiplying I with the wavelength λ (nm) and the unit transformation factor 0.836×10^{-2}. The $Q_{inci\,(\lambda)}$ is recalculated to spectral absorbed photon flux ($Q_{abs\,(\lambda)}$ µmol m^{-2} s^{-1}) by eq. (S4)[6]

$$Q_{abs\,(\lambda)} = Q_{inci\,(\lambda)} \times [1-\exp(-a_\lambda \times L)] \quad \text{(S4),}$$

where a_λ (m^{-1}) is the spectral absorptivity of the water sample and L the length of the reaction vessel (5 cm). The exponential term corrects for the "inner-filter-effect" (i.e. light absorbance by

depth in the experimental reactor). The cumulative absorbed photon flux is obtained by multiplication of $Q_{abs\,(\lambda)}$ by the reaction time and integration over a chosen wavelength range.

The apparent quantum yield (AQY, Φ) is defined as the number of moles of a product formed (or reactant degraded) per moles of absorbed photons, mol E^{-1}. In the case of MeHg photolysis, Φ is calculated by equation (S5) under experimental conditions where $[MeHg]_0$ is the initial concentration of MeHg in nmol L^{-1}, V is the volume (L) and A the cross-section area (m^2) of the reaction vessel.

$$\Phi = k_{\mathrm{pd\ abs}} \times [\mathrm{MeHg}]_0 \times V/A \qquad \text{(S5)}$$

By eqs. (S3)-(S5) the three parameters, $k_{pd\,\mathrm{inci}}$ ($m^2\,E^{-1}$), $k_{pd\,\mathrm{abs}}$ ($m^2\,E^{-1}$) and Φ (mol E^{-1}) were experimentally determined for water samples exposed to solar spectrum irradiance.

SI TEXT S3. Derivation of continuous spectral AQY models

To derive continuous spectral AQY models we first calculated the AQY ($\Phi_{\lambda1\text{-}\lambda2}$) for the wavelength-intervals 250-309 nm, 310-350 nm, 351-380 nm, 381-420 nm and 421-700 nm. The AQY was calculated by eqs. (S3) and (S4) for each of the five filters to get the AQY for each filter ($\Phi_{250\text{-}700}$, $\Phi_{310\text{-}700}$, $\Phi_{351\text{-}700}$, $\Phi_{381\text{-}700}$, and $\Phi_{421\text{-}700}$) and the fraction of absorbed irradiance, f, was summed up for respective wavelength intervals. The very small value of $\Phi_{421\text{-}700}$ was in a first step approximated to zero enabling the four remaining unknowns $\Phi_{250\text{-}309}$, $\Phi_{310\text{-}350}$, $\Phi_{351\text{-}380}$ and $\Phi_{381\text{-}420}$ to be calculated by an iterative procedure from eqs. (S5) – (S8):

$$\Phi_{250\text{-}700} = \Phi_{250\text{-}309} f_{250\text{-}309} + \Phi_{310\text{-}350} f_{310\text{-}350} + \Phi_{351\text{-}380} f_{351\text{-}380} + \Phi_{381\text{-}420} f_{381\text{-}420} + \Phi_{421\text{-}700} f_{421\text{-}700} \qquad \text{(S6)}$$

$$\Phi_{310\text{-}700} = \Phi_{310\text{-}350} f_{309\text{-}350} + \Phi_{351\text{-}380} f_{351\text{-}380} + \Phi_{381\text{-}420} f_{381\text{-}420} + \Phi_{421\text{-}700} f_{421\text{-}700} \qquad \text{(S7)}$$

$$\Phi_{351\text{-}700} = \Phi_{351\text{-}380} f_{351\text{-}380} + \Phi_{381\text{-}420} f_{381\text{-}420} + \Phi_{421\text{-}700} f_{421\text{-}700} \qquad \text{(S8)}$$

$$\Phi_{381\text{-}700} = \Phi_{381\text{-}420} f_{381\text{-}420} + \Phi_{421\text{-}700} f_{421\text{-}700} \qquad \text{(S9)}$$

The value of $\Phi_{421\text{-}700}$ was in a second step allowed to exceed zero to improve the merit-of-fit (eq. 5 in main text) of all five wavelength intervals and to yield continuous functions describing the wavelength-dependency of Φ_λ. The calculated values on $\Phi_{250\text{-}309}$, $\Phi_{310\text{-}350}$, $\Phi_{351\text{-}380}$, $\Phi_{381\text{-}420}$ and $\Phi_{421\text{-}700}$ were used to optimize the spectral AQY models, eqs. (5) – (10), by minimizing the merit-of-fit as and linear relationships with the measured values on $\Phi_{250\text{-}700}$, $\Phi_{310\text{-}700}$, $\Phi_{351\text{-}700}$, $\Phi_{381\text{-}700}$, and $\Phi_{421\text{-}700}$, as explained in the main text.

SI TEXT S4. Spectral incidence irradiance models

Spectral incidence irradiance models and AQY models derived from the very dark lake (ANG1)

In **Figs. S6**, model fits derived for the darkest water sample (ANG1, representing an end-member lake with a very high DOC, 39 mg L^{-1}, and high SUVA, 4.7 L mg^{-1} m^{-1}) are illustrated, where incidence irradiation and AQY models are given as eqs. (S10) – (S12) and eqs. (S13) – (S15), respectively.

Spectral incidence irradiation models (ANG1 – dark end-member lake):

Power function: $k_{pd\,(\lambda)\,inci}\ (m^2\ E^{-1}) = 10^{20.6} \times \lambda^{-8.72}$ (S10)

Vähätalo function: $k_{pd\,(\lambda)\,inci}\ (m^2\ E^{-1}) = 95.4 \times (10^{-0.01\lambda})$ (S11)

Exponential function: $k_{pd\,(\lambda)\,inci}\ (m^2\ E^{-1}) = \exp\text{-}(2.01+0.024(\lambda\text{-}290))$ (S12)

Spectral AQY models (ANG1 – dark end-member lake):

Power function: $AQY_{\lambda}\ (nmol\ E^{-1}) = 4.00 \times 10^{17.0} \lambda^{-7.82}$ (S13)

Vähätalo function: $AQY_{\lambda}\ (nmol\ E^{-1}) = 3.26 \times (10^{-0.008\lambda})$ (S14)

Exponential function: $AQY_{\lambda}\ (nmol\ E^{-1}) = \exp\text{-}(4.05+0.02(\lambda\text{-}290))$ (S15)

Spectral incidence irradiance models derived from the clear lake (LJU)

In **Fig. S5**, fits of the models derived for the clear lake LJU (having a low DOC of 2.9 mg L^{-1} and a low SUVA of 1.8 L mg^{-1} m^{-1}), are illustrated, where the final incidence irradiation models are given as equations (S16) – (S18).

Spectral incidence irradiation models (LJU – clear lake):

Power function: $k_{pd\,(\lambda)\,inci}\ (m^2\ E^{-1}) = 10^{29.3} \lambda^{-12}$ (S16)

Vähätalo function: $k_{pd\,(\lambda)\,inci}\ (m^2\ E^{-1}) = 2280 \times (10^{-0.013\lambda})$ (S17)

Exponential function: $k_{pd\,(\lambda)\,inci}\ (m^2\ E^{-1}) = \exp\text{-}(0.943+0.031(\lambda\text{-}290))$ (S18)

SI TEXT S5. Calculation of solar spectral irradiance reaching the lake surface. For the Swedish lake data set, daily integrated downwelling scalar irradiance spectra was available for the year 2009 at the locations of the 1033 Swedish lakes, as described in Koehler et al.[7] These spectra (covering the wavelength interval 290-700 nm) were obtained by simulations conducted with the libRadtran radiative transfer package.[8,9] In short, radiation for cloud-free conditions was simulated using relevant atmospheric input from the Integrated Forecasting System of the European Centre for Medium-Range Weather Forecasts (https://www.ecmwf.int/en/publications/ifs-documentation), surface albedo from the International Geosphere Biosphere Program (IGBP) database[10] (part of the libRadtran package), and aerosols representative of Swedish conditions. The cloud-free radiation was corrected for the effect of clouds using hourly cloud information from the MESAN analysis system.[11]

For the purpose of upscaling to other major regions of the world, solar radiation calculations were conducted for the 25 globally distributed lakes. To create a globally applicable approach, we combined a previously developed method for reconstructing spectral UV irradiance[12] with components of the Ozone Monitoring Instrument (OMI) satellite UV algorithm.[13,14] For this, we used the libRadtran radiative transfer simulation with total ozone column and cloud optical depth from the OMI UV overpass data, available at a daily time resolution (https://avdc.gsfc.nasa.gov/index.php?site=2057856112&id=79). Information on the albedo was taken from the IGBP library, and the default aerosol model of libRadtran was adjusted to match a climatological[15] monthly aerosol optical depth, single scattering albedo, and asymmetry parameter at 550 nm. In this manner, hourly spectral solar irradiances from 290 to 700 nm reaching Earth's surface were simulated for the period 2005—2016 for each of the 25 lakes. Data were finally aggregated to daily and monthly values of incident solar radiation, divided into global and diffuse components.

Calculations of below water surface down-welling irradiance. Direct and indirect solar irradiance data were transferred to down-welling scalar irradiance below the water surface of lakes following the outline of Fichot and Miller.[16] In short, the diffusive ($f_{\text{diffusive}}$) and direct (f_{direct}) fractions of the irradiance above the air-water interface (0^+) were corrected for losses at the water surface by calculating the transmittance

$T(\lambda)$ of both fractions, by equation (S19).

$T(\lambda) = f_{\text{diffusive}}(\lambda,0^+)0.934 + f_{direct}(\lambda,0^+)(1- R_{Spec}(\lambda))$ (S19)

The specular reflection (R_{Spe}) of direct solar irradiance at the lake surface was calculated using Fresnel's law of reflection.

$$R_{Spec} = 0.5[\frac{sin(\theta-\theta w,\lambda)2}{sin(\theta+\theta w,\lambda)2} + \frac{\tan(\theta-\theta w,\lambda)2}{\tan(\theta+\theta w,\lambda)2}] \quad \text{(S20)}$$

The zenith angle of irradiance below the water surface ($\theta_{w,\lambda}$) was calculated from the zenith angle of solar irradiance (θ), by equation (S20).

$$\theta_{w,,\lambda} = \sin^{-1}(\sin(\theta)/n_\lambda] \quad \text{(S21)}$$

The wavelength-dependent refractive index of water (n_λ) from 290 to 700 nm was calculated in agreement with Quan and Fry.[17]

$$n_\lambda = 1.31279 + 15.762\lambda^{-1} - 4382\lambda^{-2} + 1.1455\times10^6\lambda^{-3} \quad \text{(S22)}$$

Finally, the underwater average cosine for down-welling irradiance, $\mu_d(\lambda)$, was calculated following Fichot and Miller.[16]

$$\frac{1}{\mu d(\lambda)} = \frac{1-fdiff(\lambda)}{cos(\theta)} + \frac{fdiff(\lambda)}{0.859} \quad \text{(S23)}$$

The down-welling irradiance below the water surface ($E(\lambda,0^-)$) was finally obtained by multiplying the scalar irradiance above the water surface, $E(\lambda,0^+)$, with $T(\lambda)$ and $\frac{1}{\mu d(\lambda)}$ by hour and integrating over 24 hours.

$$E(\lambda,0^-, \text{day}) = \int_{0h}^{24h} \frac{E(\lambda,0+,t)T(\lambda,t)}{\mu d(\lambda,t)} dt \quad \text{(S24)}$$

Supporting Text for the Results and Discussion Section

SI TEXT S6. Difference in RTS quenching effects between dark and clear lake models and the Swedish data set of 1033 lakes

All DOC classes of the Swedish lake data set showed a smaller quenching effect in comparison to the ANG12 and LS lakes, by which the dark model was developed. Therefore, inclusion of the $a\exp(b/(1+UVA/UVA_{1/2})$ term in the dark lake AQY model enhanced the MeHg photodegradation in all DOC classes (**Fig. S13a**). In contrast, only lakes in the two lowest DOC classes (2 – 5 mg L^{-1}) showed less effect of RTS quenching than lake LJU. Therefore, inclusion of the $a\exp(b/(1+UVA/UVA_{1/2})$ term in the clear lake AQY model decreased the MeHg photodegradation in lakes with DOC higher than 5 mg L^{-1} (**Fig S13b**).

SI TEXT S7. Comparison of outcome between AQY and incidence radiation models

Incident radiation models overestimate MeHg photodegradation in clear lakes (**Figs. S15, S16**). This is further exemplified by a study in the Arctic region, where *In Situ* incubations combined with an incident radiation model using PAR as input variable resulted in a reported MeHg photodegradation of 1 300 ng m^{-2} for 100 ice-free days in the clearwater Toolik lake, USA.[18] The study was criticized for only considering the PAR waveband, and the MeHg photodegradation was recalculated to 570 ng m^{-2} by an incident radiation model taking UV-B and UV-A wavebands into consideration.[19] Applying our clear lake AQY_{quench} model to the same input data[18] (MeHg concentrations, lake water absorbance and solar irradiance), we estimate the MeHg photodegradation at 360 ng m^{-2} for the 100 ice-free days (**Table S10**).

In dark lakes, the overestimated significance of PAR by incidence radiation models may be partly compensated for by corresponding underestimates of the influence of the UV-A and UV-B regions. These compensatory biases could explain why the incident radiation model used by Li et al.[20] to estimate MeHg photodegradation in the dark and very shallow waters of the Florida Everglades (1240 ng m^{-2} y^{-1}, **Table S9**) is in reasonably good agreement with our estimate of 1510 ng m^{-2} y^{-1}, using the AQY_{quench} model. Also for the 1033 Swedish boreal lakes data set, average estimates of MeHg photodegradation calculated by incident light and AQY models give quite similar results (**Table S11**), despite systematic differences between the two types of models in individual lakes

and in DOC and UVA classes of lakes (**Figs. S17, S18**). In clear lake water, where only a fraction of the irradiance is absorbed in experimental water (so called "thin solutions"), incident radiation models largely overestimate MeHg photodegradation as compared to the AQY model. As demonstrated for the data set of 1033 Swedish lakes in **Figs. S17 and S18** models based on incident radiation underestimate the photodegradation in very dark lakes (e.g. by 17%, calculated as 100(1.0 – slope), in lakes with 22-50 mg L^{-1} DOC and by 20 % in lakes with > 100 m^{-1} absorbance of $UV_{254\,nm}$) while the same model overestimates the photodegradation in the most clear lakes (e.g. by 68% in lakes with 2.0 – 3.0 mg L^{-1} DOC and by 25% in lakes with 1 – 10 m^{-1} absorbance of UV_{254}. Therefore, in a large data set, the effects of under- and overestimation of dark and clear lakes, respectively, partly cancel out and in average models based on incident and absorbed photons may give results on the same order of magnitude. However, it should be noted that only AQY models correctly represents the theory of photochemistry.

Supplementary Figures S1-S18

Figure S1 – Spectrum of solar simulator and photon absorption by experimental lakes
Figure S2 – Experimental data using cut-off filters in two dark lakes (ANG, LS)
Figure S3 – Experimental data using cut-off filters in the clear lake (LJU)
Figure S4 – Experimental data full spectrum in 25 globally distributed lakes
Figure S5 – Measured vs modeled AQY and $k_{pd\ abs}$ dark lake model (ANG12+LS)
Figure S6 –Measured vs modeled AQY and $k_{pd\ inci}$ clear lake model (LJU)
Figure S7 – Measured vs modeled AQY and $k_{pd\ inci}$ dark lake end-member (ANG1)
Figure S8 – Location of lakes used in experiments and in modelling and upscaling calculations
Figure S9 – Measured vs modeled AQY and $k_{pd\ abs}$ evaluated by 25 global lakes
Figure S10 – Relationship between AQY and $SUVA_{254}$ and $1/(1+UVA_{/UVA1/2}))$ in 25 global lakes
Figure S11 –AQY_{quench} models derived from clear and dark lakes
Figure S12 – Relationships between DOC and MeHg in three global regions
Figure S13 – Dark and clear lake AQY model prediction in 1033 Swedish lakes
Figure S14 – Spectral- and depth-dependency of MeHg photodegradation in 1033 Swedish lakes
Figure S15 – Spectral, depth-integrated MeHg degradation in dark (ANG) and clear (LJU) lakes
Figure S16 – Depth-integrated MeHg degradation by absorbed and incidence light models
Figure S17 – Comparison of $k_{pd\ inci}$ and $k_{pd\ abs}$ models in Swedish lakes by DOC classes
Figure S18 – Comparison of $k_{pd\ inci}$ and $k_{pd\ abs}$ models in Swedish lakes by UVA classes

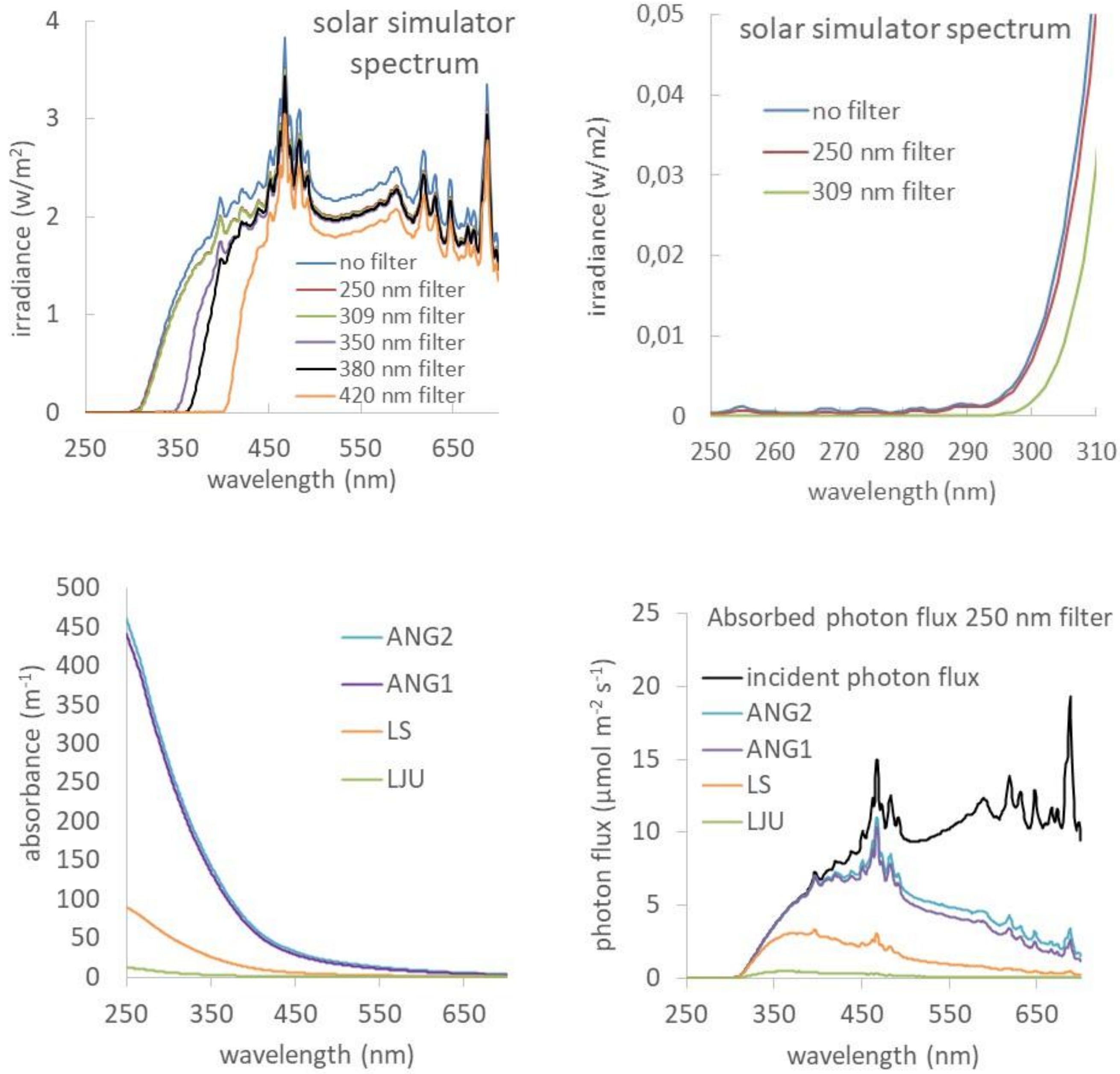

Figure S1. *Top left*: Solar simulator xenon lamp spectrum without and with cut-off filters. *Top right*: close-up demonstrating effect of 250 and 309 nm filters in the UV-C and UV-B range. *Bottom left*: spectral absorption of the four experimental samples from the three lakes ANG, LS and LJU. *Bottom right*: absorbed photon flux passing the 250 nm filter in the three experimental lakes.

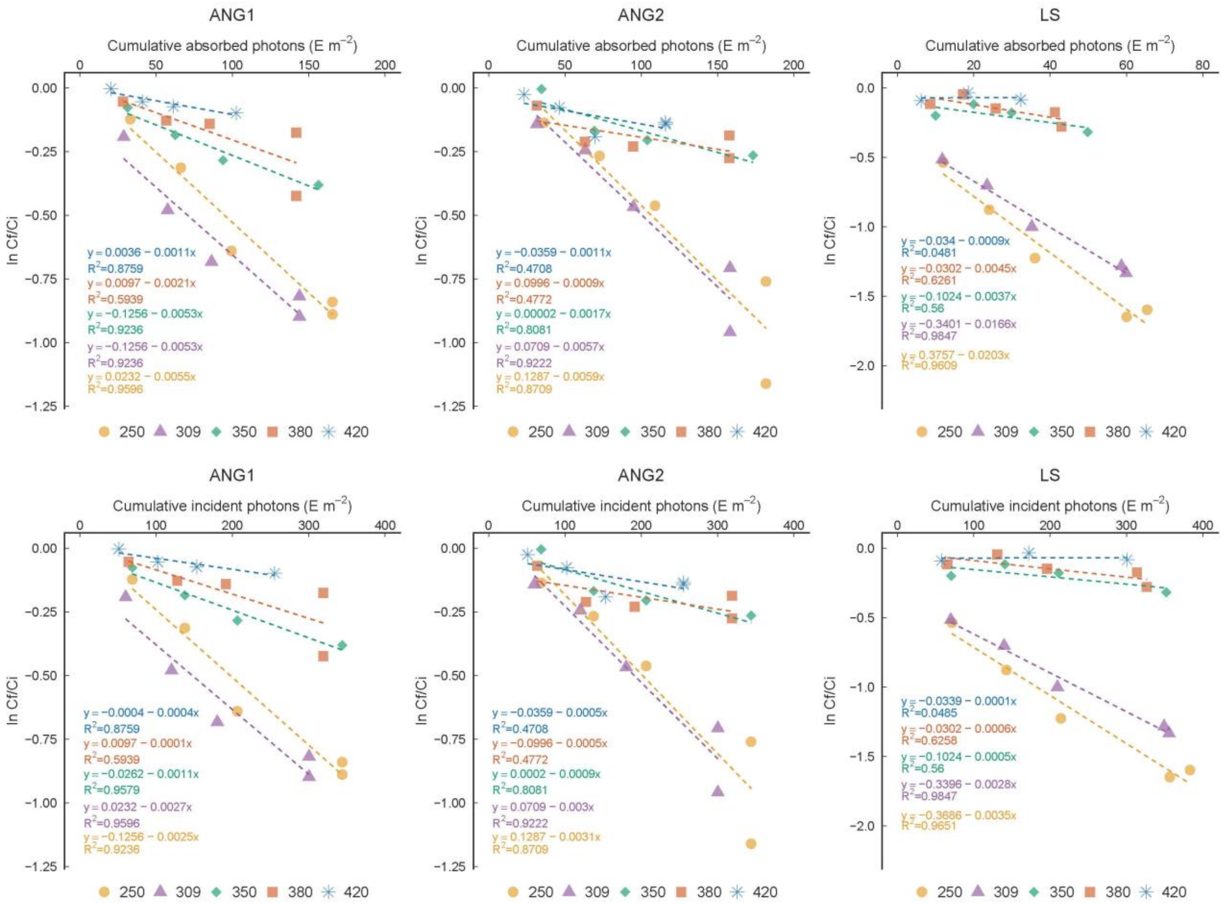

Fig. S2. Dark lakes (ANG12, LS) experimental MeHg photodegradation as a function of cumulative absorbed (top) and incident (bottom) photon flux in presence of optical cut-off filters. Data are presented after subtraction of dark control degradation. Due to uncertainties in the absolute amount of MeHg added at time 0 and small differences in MeHg photodegradation after 3 hours, linear regressions were derived from data obtained after 6, 12, 18 and 30 h. Duplicate samples were determined at 30 h of reaction. In the lake LS sample the cut-off filter 420 nm resulted in very small photodegradation not significantly different from the dark controls.

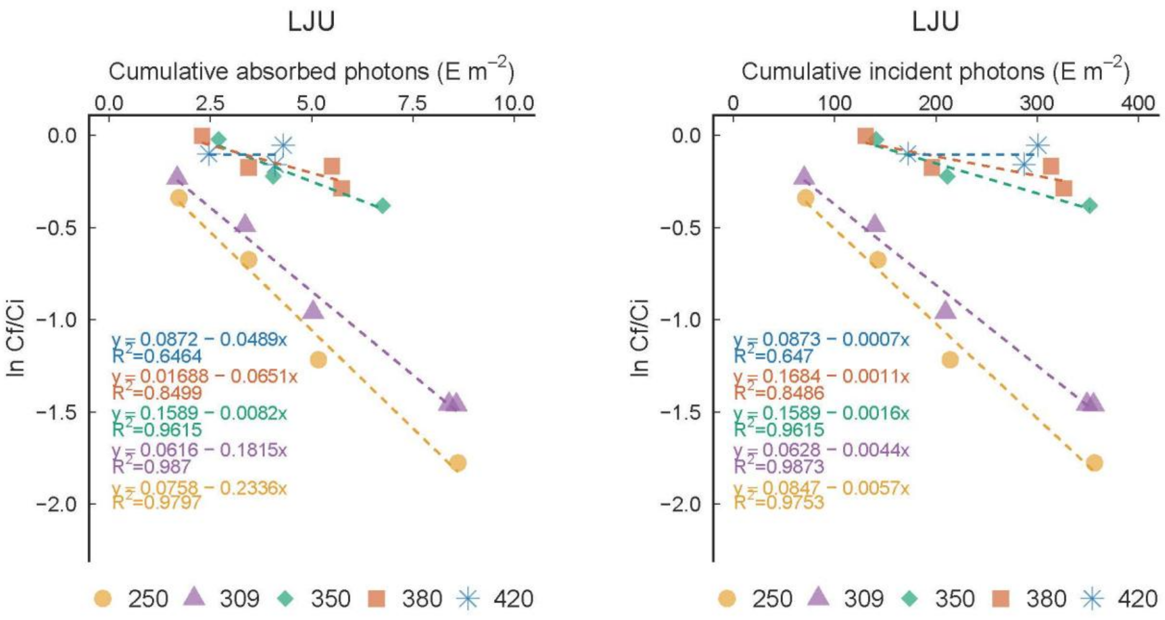

Fig. S3. Clear lake (LJU) experimental MeHg photodegradation as a function of cumulative absorbed (left) and incident (right) irradiation in presence of optical cut-off filters (250, 309, 350, 380 and 420 nm). Data are presented after subtraction of dark control MeHg degradation. Due to uncertainties in the absolute amount of MeHg added at time 0 and small differences in MeHg photodegradation after 3 hours, linear regressions were derived from data obtained after 6, 12, 18 and 30 h of reaction. Duplicate samples were determined at 30 h of reaction.

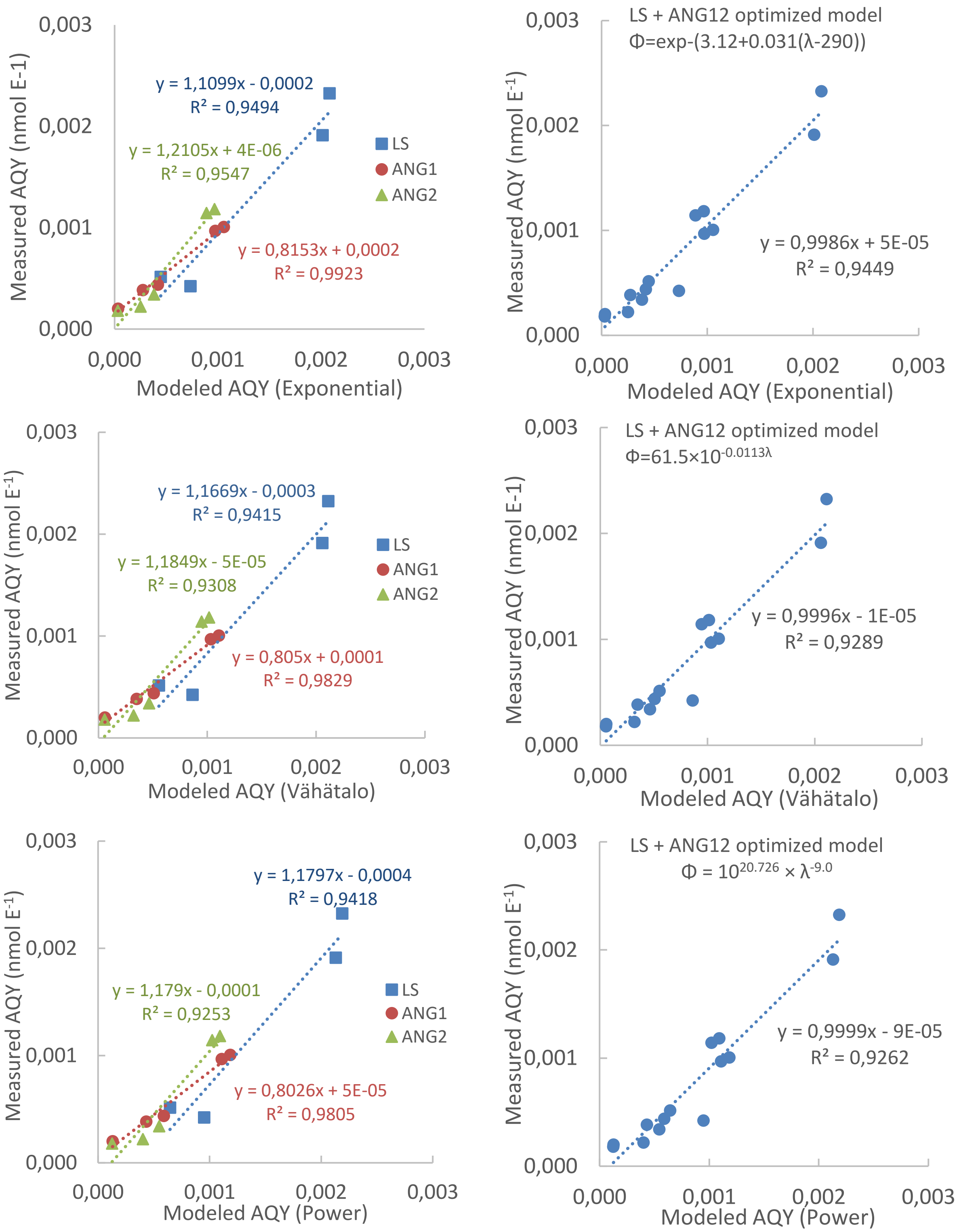

Fig. S4. Optimized AQY models derived from experimental data using cut-off filters for dark lake water samples LS, ANG1 and ANG2. *Left column:* Linear relationships between modeled and measured AQY reported for each of the three data sets were not statistically different. *Right column:* AQY models, eqs. (5) - (7), parameterized to yield 1:1 linear regressions, as derived for the combined data set of all three lake samples. The five data points for each data set represent the MeHg photodegradation for wavelength intervals 421-700, 381-700, 351-700, 310-700 and 250-700 nm in the direction from small to large AQY values.

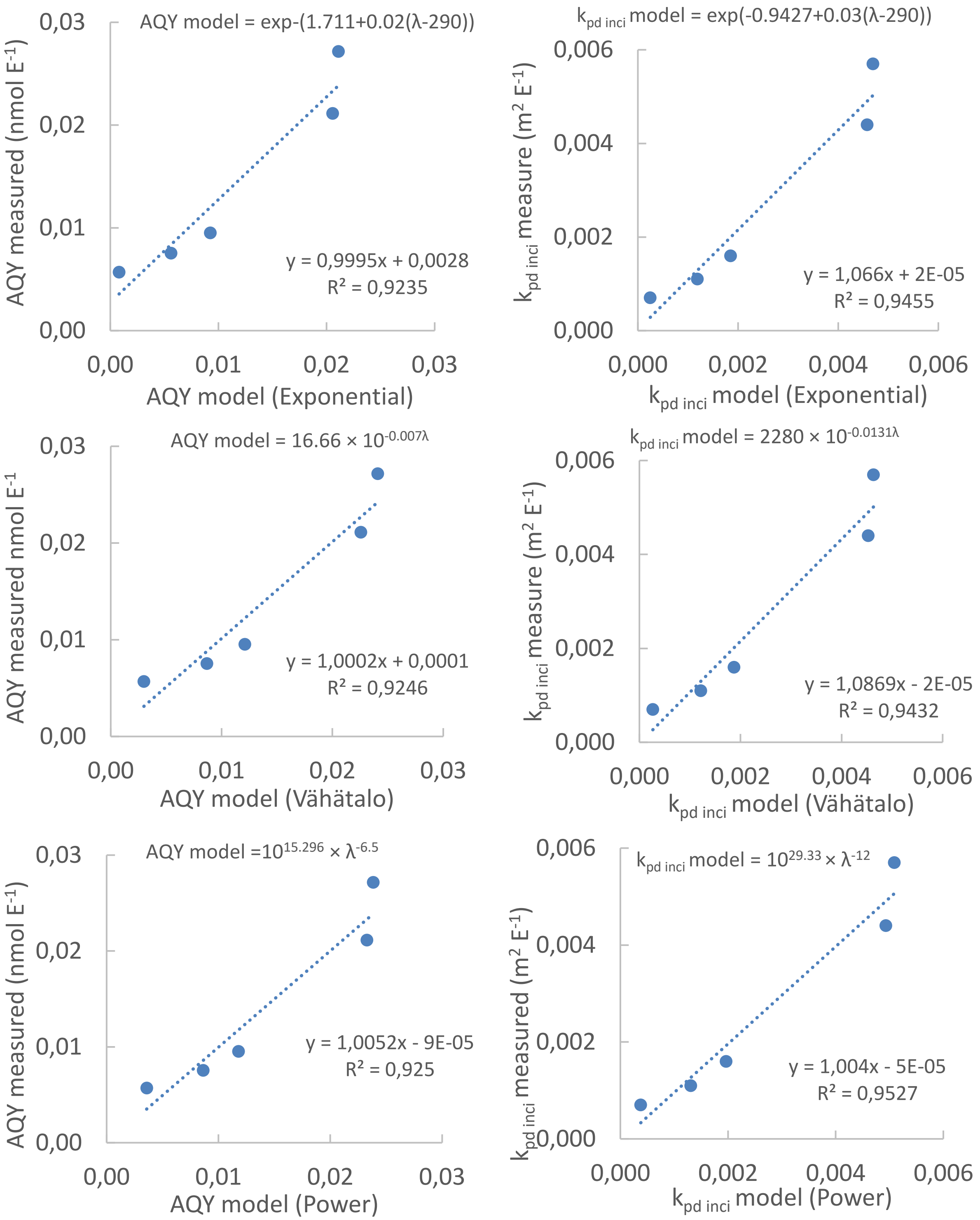

Fig. S5. Optimized AQY models derived from experimental data using cut-off filters for clear lake (LJU) samples. *Left column*: Relationship between modeled, eqs. (8) – (10) and measured apparent quantum yield (AQY) for MeHg photodegradation. *Right column*: Relationship between modeled and measured MeHg photodegradation constants derived from incident radiation ($k_{pd\ inci}$). The five data points represent the MeHg photodegradation for the wavelength intervals 421-700, 381-700, 351-700, 310-700 and 250-700 nm in the direction from small to large AQY values.

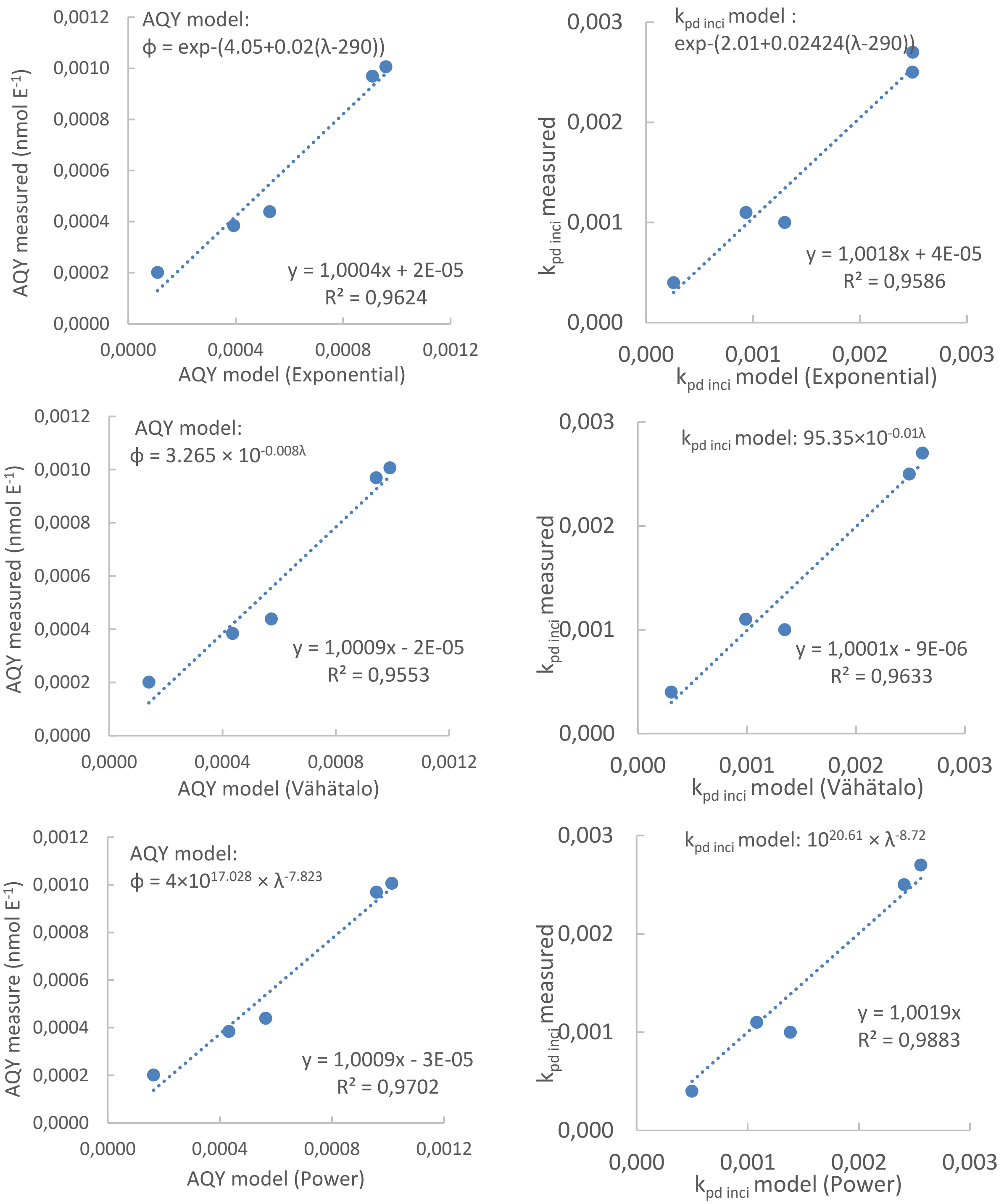

Fig. S6. Dark lake (ANG1) model outcome plotted versus measured experimental MeHg photodegradation data. *Left column*: Relationship between modeled, eqs. (S13) – (S15) and measured apparent quantum yield (AQY) for MeHg photodegradation. *Right column*: Relationship between modeled, eqs. (S10) – (S12), and measured MeHg photodegradation constants derived from incident radiation ($k_{pd\ inci}$). The five data points represent the MeHg degradation in wavelength intervals 421-700, 381-700, 351-700, 310-700 and 250-700 nm in the direction from small to large AQY values.

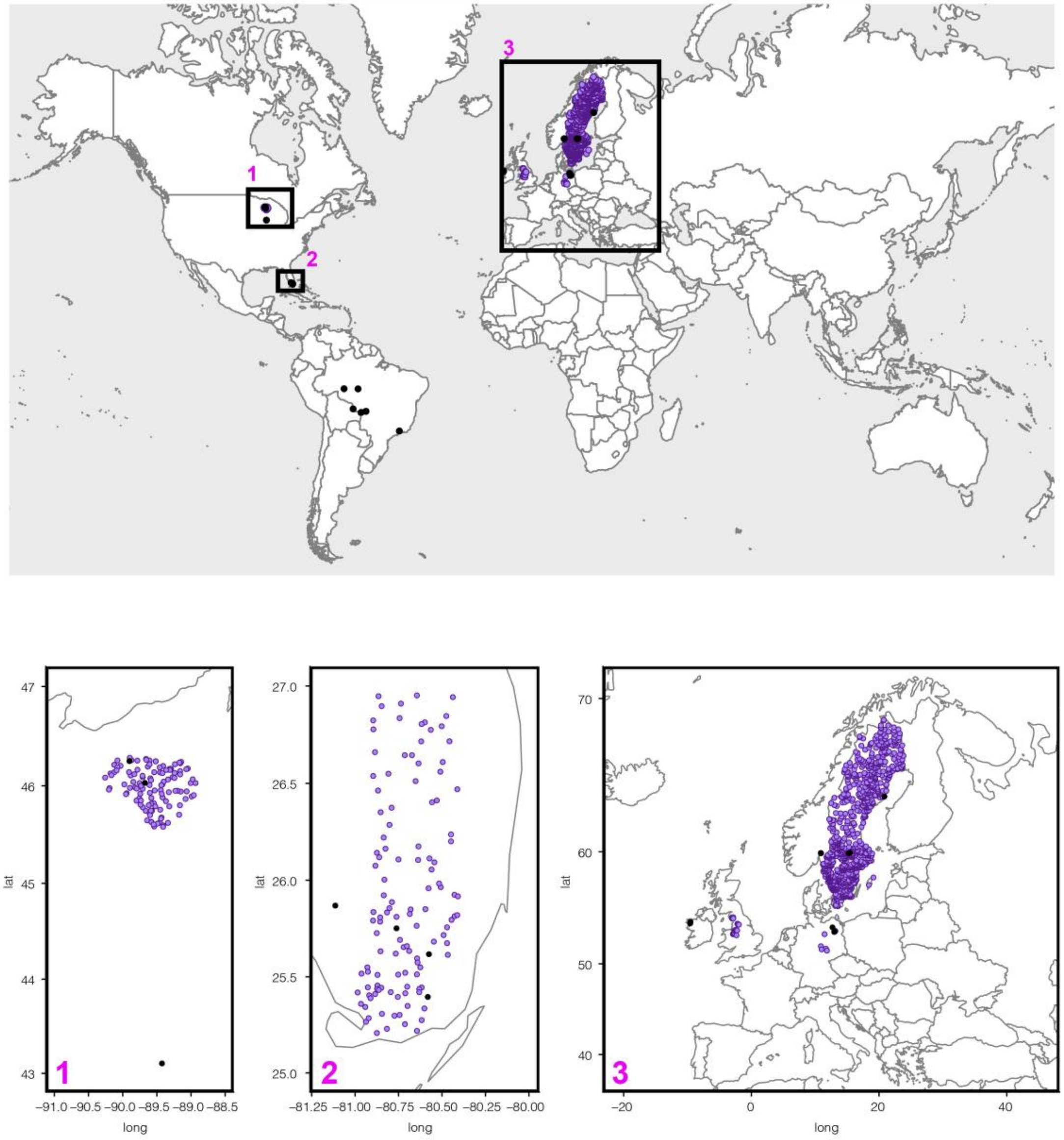

Figure S7. Location of lakes used in this study. Black dots denote the 25 globally distributed lakes for which experimental data on MeHg photodegradation were generated (12 in Europe, 3 in Wisconsin, USA, 4 in the Florida Everglades, USA and 6 in tropical S America, Brazil, **Table S4, S5**). Violet dots represent lakes with reported light absorbance and MeHg concentration data in Wisconsin (119 lakes, inset 1, data **Table S12a**), Florida Everglades (118 sampling stations, inset 2, data **Table S12c,d**), UK (n=23, data **Table S12b**), Irish (n=3), German (n=3) and Swedish lakes (n=1033) (inset 3).

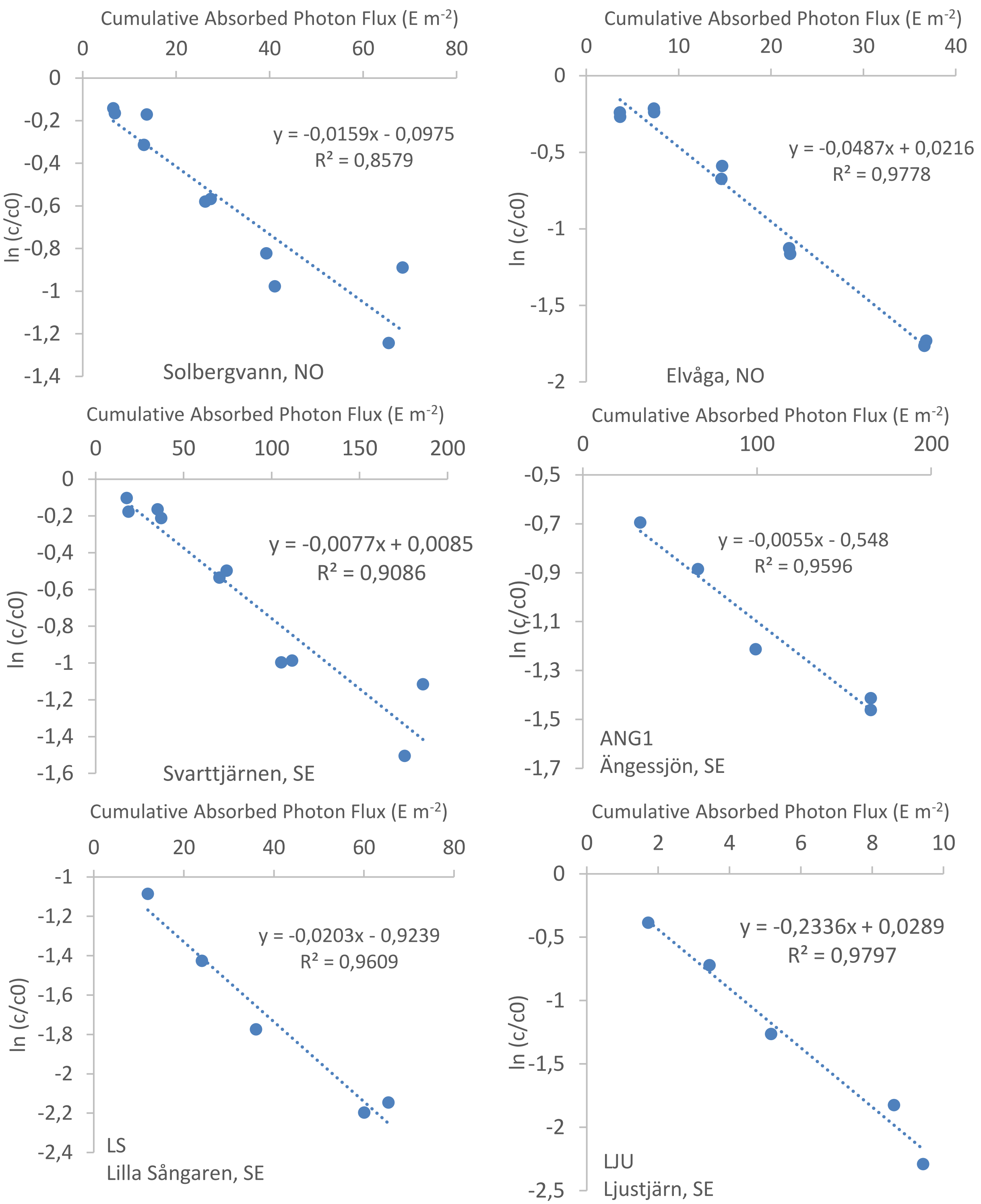

Fig. S8. Temperate Europe: Norway (2 lakes), Sweden (4 lakes). Experimental MeHg photodegradation data for lake water exposed to full irradiance spectrum (250 – 700 nm). Initial concentrations (c_0) and concentrations of MeHg v.s. cumulative absorbed photon flux after subtraction of dark controls. Data points correspond to times 3, 6, 12, 18 and 30 h (n=2 at each time point).

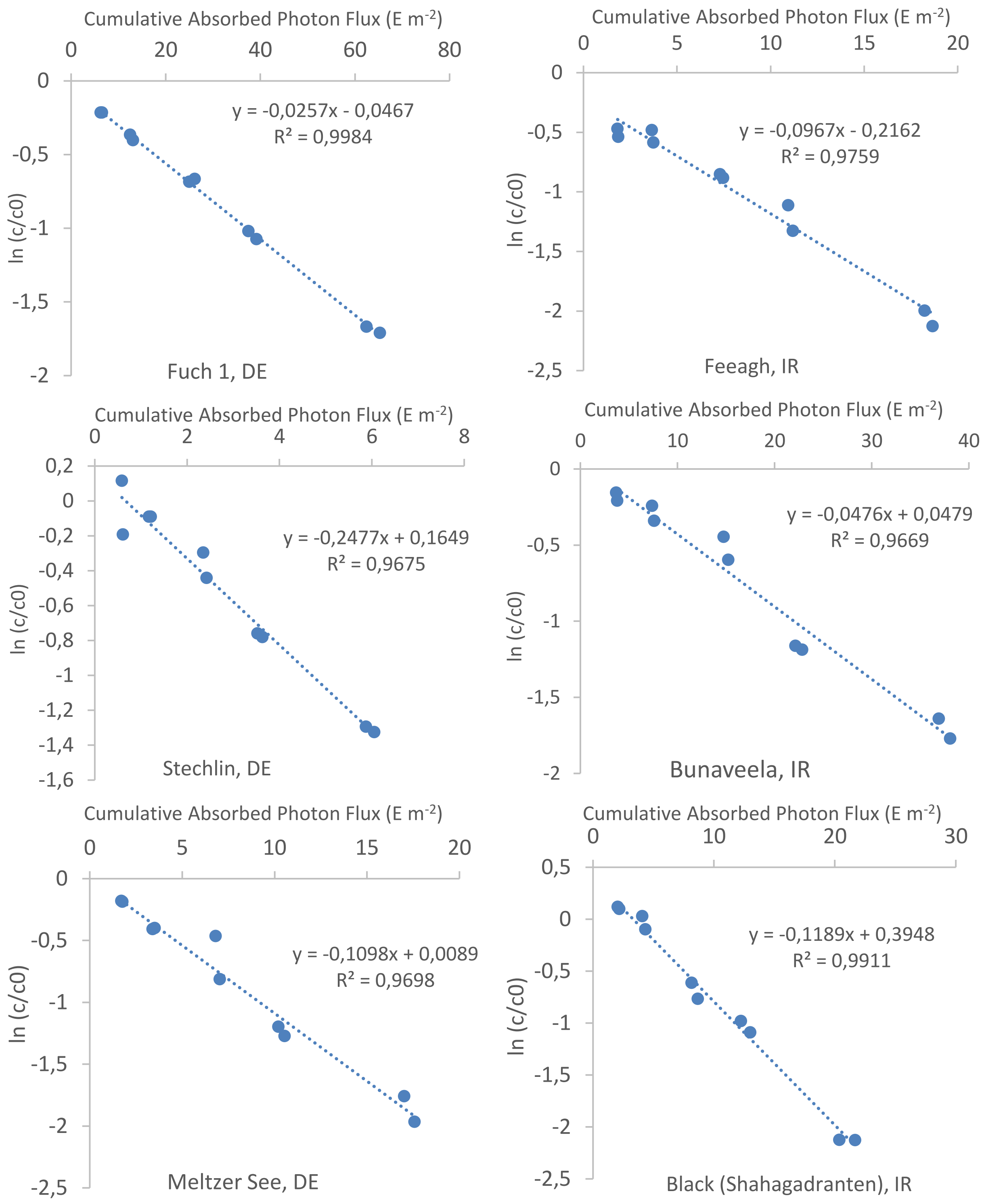

Fig. S8 cont. Temperate Europe: Germany (3 lakes), Ireland (3 lakes). Experimental MeHg photodegradation data for lake water exposed to full irradiance spectrum (250 – 700 nm). Initial concentrations (c_0) and concentrations of MeHg v.s. cumulative absorbed photon flux after subtraction of dark controls. Data points correspond to times 3, 6, 12, 18 and 30 h (n=2 at each time point).

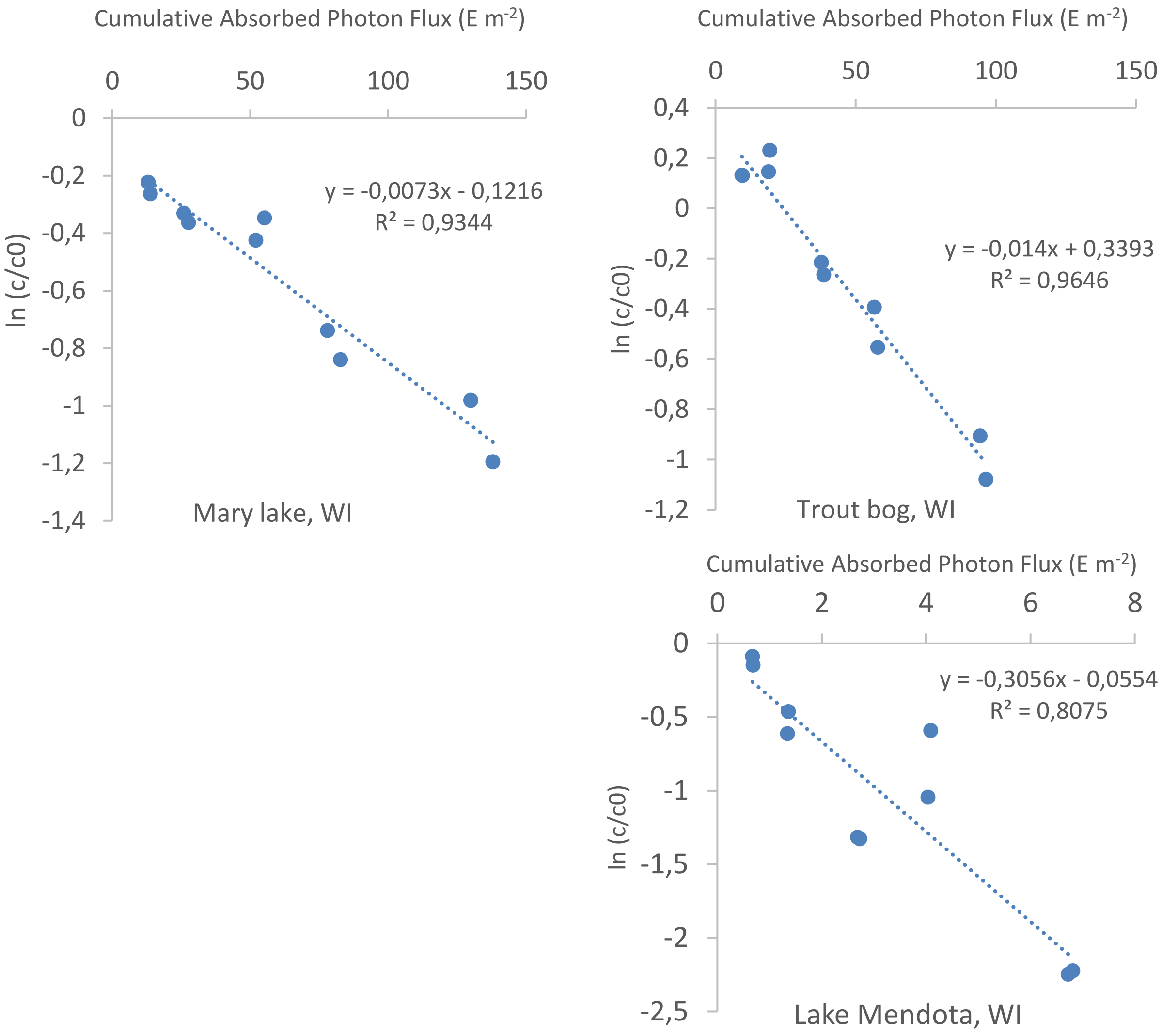

Fig. S8 cont. Temperate North America: Wisconsin (3 lakes). Experimental MeHg photodegradation data for lake water exposed to full irradiance spectrum (250 – 700 nm). Initial concentrations (c_0) and concentrations of MeHg v.s. cumulative absorbed photon flux after subtraction of dark controls. Data points correspond to times 3, 6, 12, 18 and 30 h (n=2 at each time point).

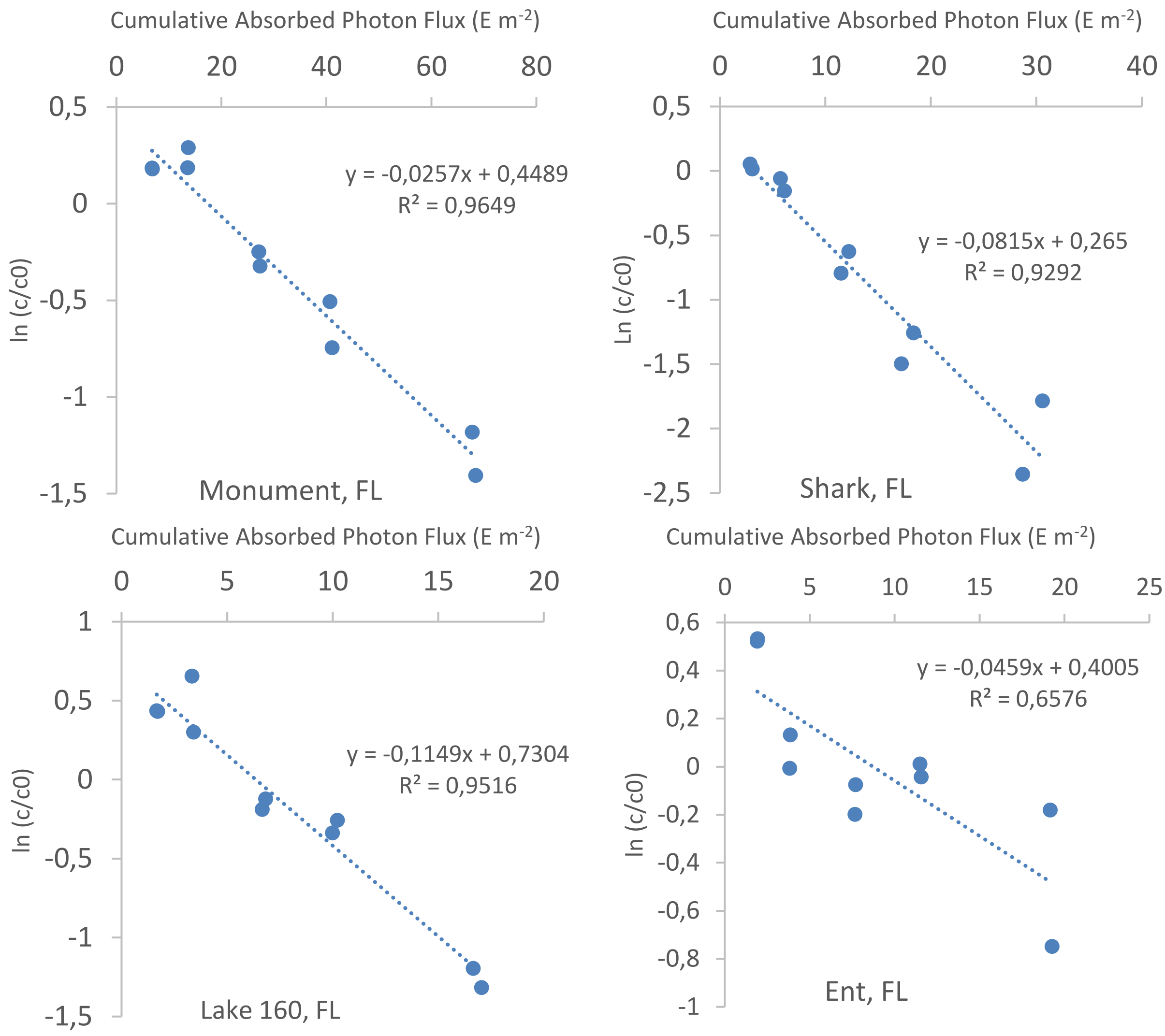

Fig. S8 cont. Sub-tropical North America: Florida Everglades (4 lakes). Experimental MeHg photodegradation data for lake water exposed to full irradiance spectrum (250 – 700 nm). Initial concentrations (c_0) and concentrations of MeHg v.s. cumulative absorbed photon flux after subtraction of dark controls. Data points correspond to times 3, 6, 12, 18 and 30 h (n=2 at each time point).

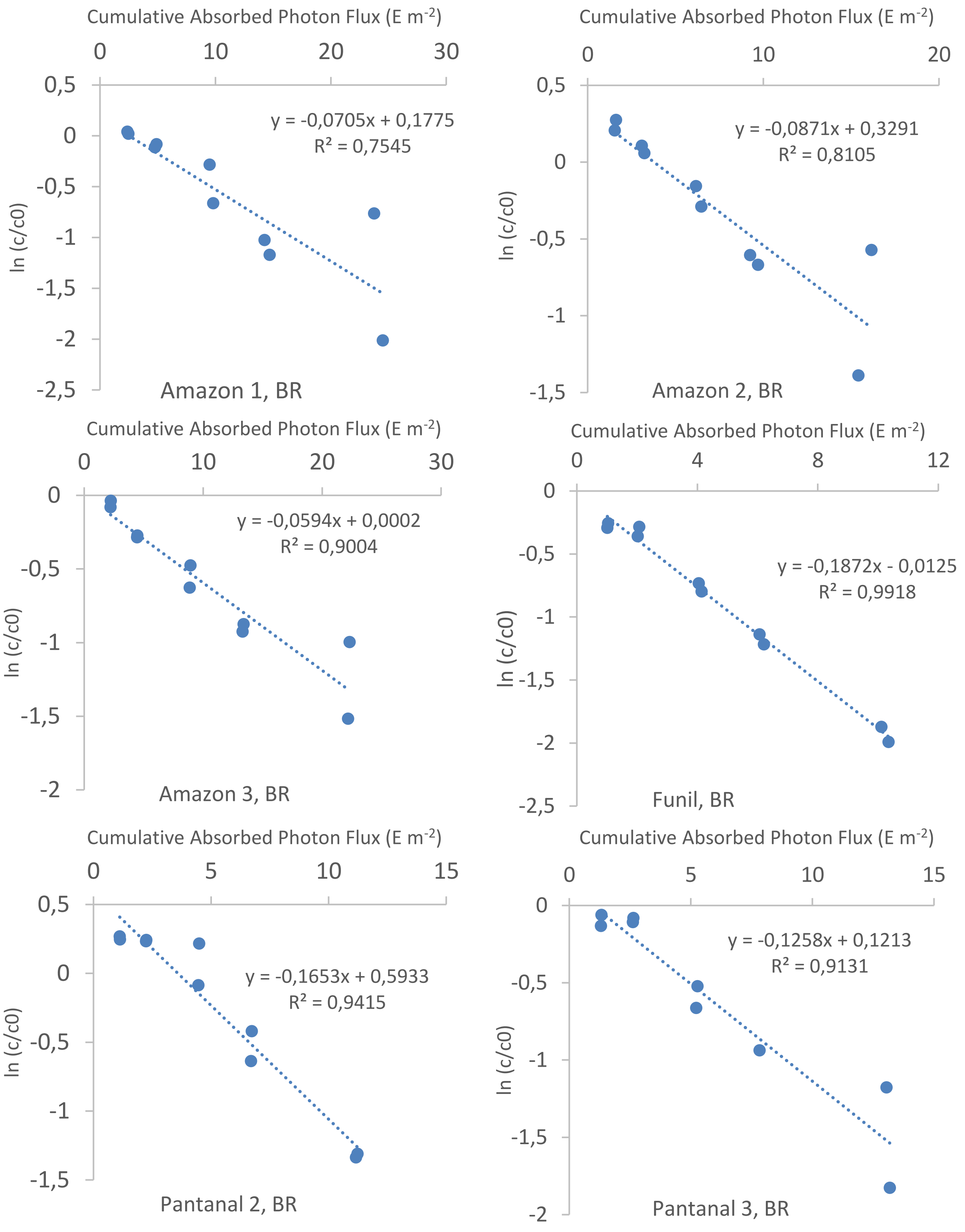

Fig. S8 cont. Tropical South America: (6 lakes). Experimental MeHg photodegradation data for lake water exposed to full irradiance spectrum absorption (250 – 700 nm). Initial concentrations (c_0) and concentrations of MeHg v.s. cumulative absorbed photon flux after subtraction of dark controls. Data points correspond to times 3, 6, 12, 18 and 30 h (n=2 at each time point).

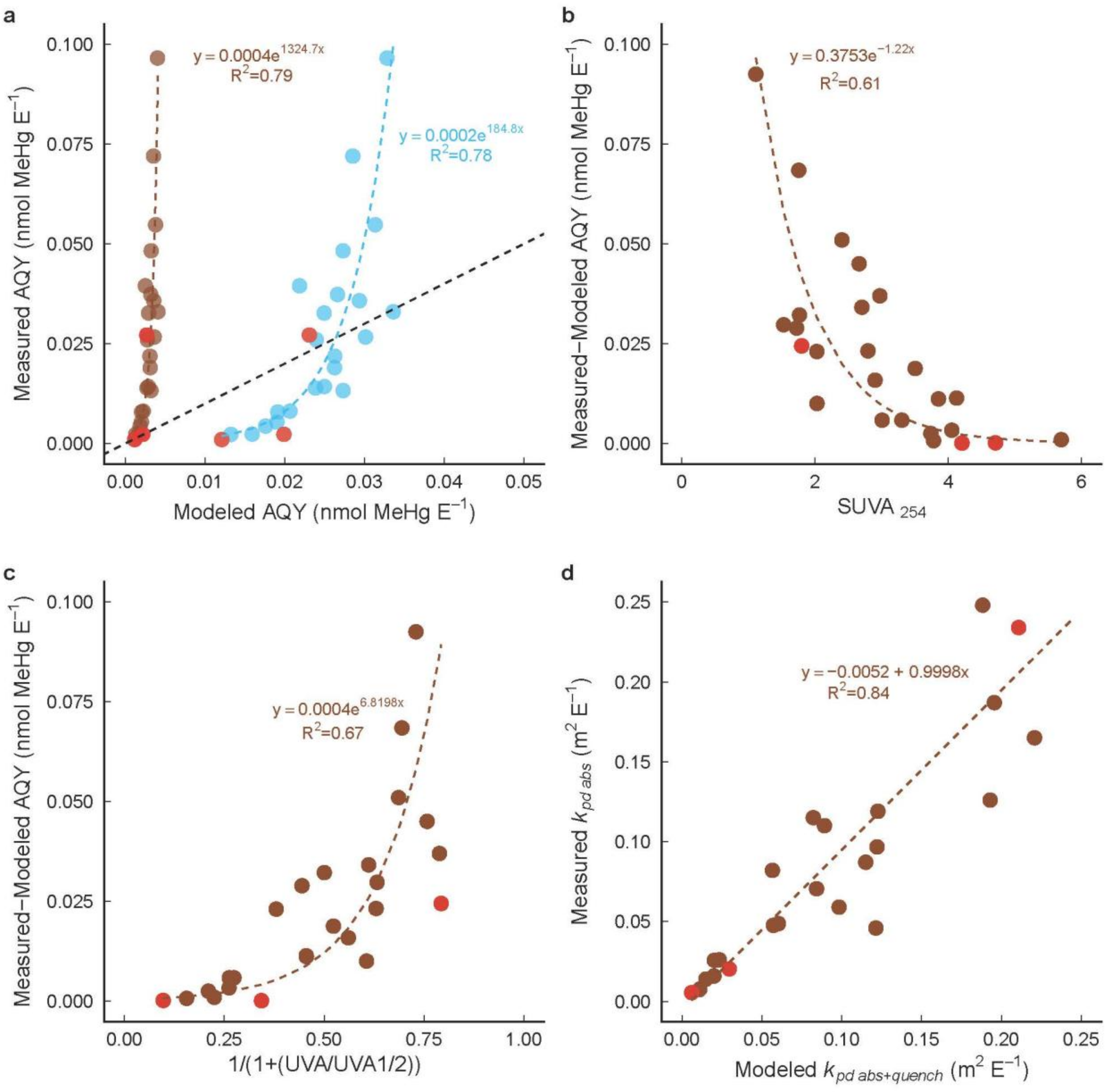

Fig. S9. a) Measured versus modeled Apparent Quantum Yields (AQY, ϕ, nmol E^{-1}) for 25 globally distributed lakes with no quenching term included in the model. The brown dots represent model calculations derived from the dark lakes (LS+ANG12), eq. (7), and blue dots calculations using the clear lake (LJU), eq (10) model. The three red dots represent the three lakes LS, ANG1 and LJU with highest AQY at LJU. Data points below the dotted 1:1 line are underestimated by the models and data above the line are overestimated. **b)** Relationship between the prediction errors (measured – modeled AQY) of the dark model, eq. (7), and measured $SUVA_{254}$. The small error of the high $SUVA_{254}$ (dark) lakes mean they are better predicted by the dark model while low $SUVA_{254}$ (clear) lakes are not. **c)** The discrepancy between modeled AQY by the dark lake model, eq. (7), and measured AQY, plotted versus the quenching term ($1/(1+UVA/UVA_{1/2})$). **d)** Measured $k_{pd\ abs}$ ($k_{pd\ abs}$ = AQY/([MeHg]V/A)) vs. modelled $k_{pd\ abs+quench}$ by the dark lakes model, eq. (17). The factor 1/([MeHg]V/A is 3.725 for the 22 globally distributed lakes (brown dots) and 5.5, 8.0 and 8.5, respectively, for the three experimental lakes LS, ANG1 and LJU (red dots). UVA is the decadic UV absorption at 254 nm.

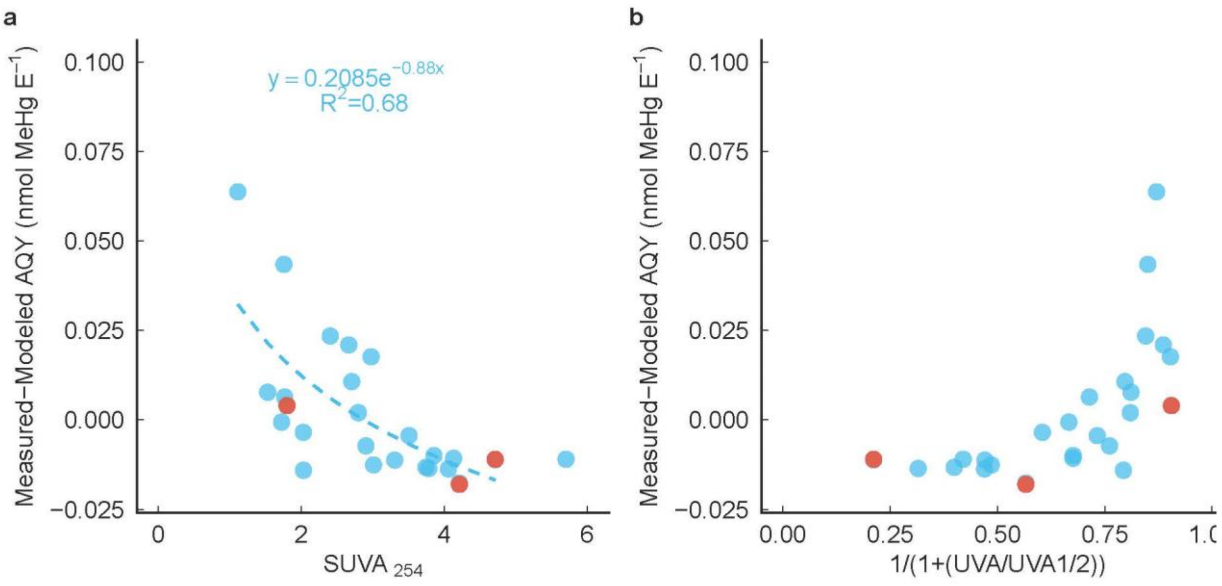

Fig. S10. Relationship between the prediction error of eq. (10) (discrepancy between measured AQY and AQY generated by the clear lake model without inclusion of the quenching term), and **a)** measured $SUVA_{254}$, and **b)** a $1/(1+UVA/UVA_{1/2})$, a proxy for the relative differences in quenching, among all 25 globally distributed lakes. UVA is the decadic UV absorption at 254 nm. The three red dots represent the lake water samples from LS, ANG1 and LJU (where the latter has the lowest $SUVA_{254}$).

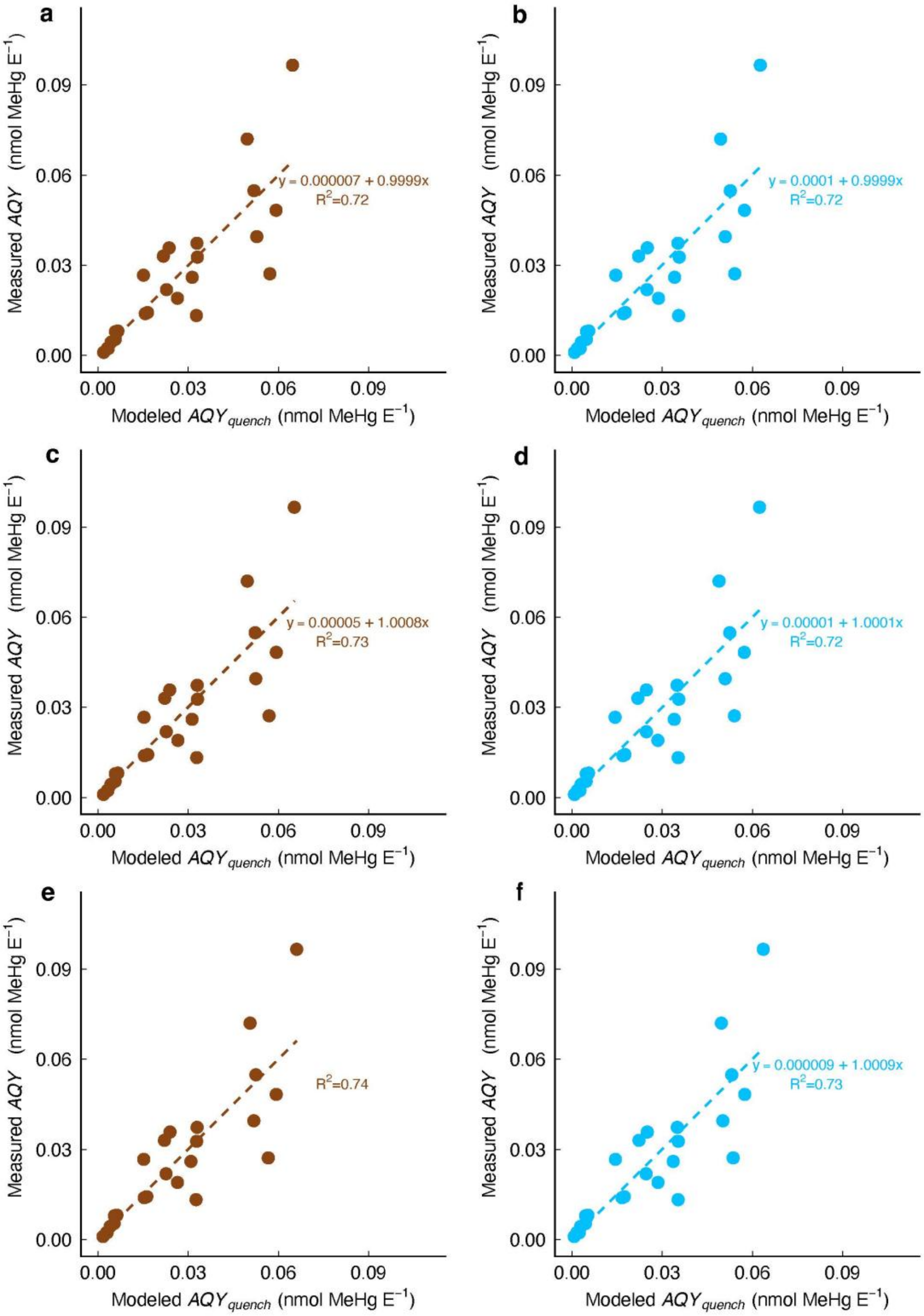

Fig. S11. Modeled AQY_{quench} (nmol E^{-1}) plotted versus experimentally determined AQY for 25 globally distributed lakes. Left column shows results by the dark lake model (LS+ANG12) and right column shows results by the clear lake model (LJU). In both cases the full spectrum AQY ($\Sigma\Phi_{250\text{-}700}$) was multiplied by the term $a\exp(b/(1+\text{UVA}/\text{UVA}_{1/2})$ to account for differences in quenching. Equations given in figures corresponds to **a)** dark lake model power function eq. (11), **b)** clear lake model power function eq. (14), **c)** dark lake model Vähätalo function eq. (12), **d)** clear lake model Vähätalo function eq. (15), **e)** dark lake model exponential function eq. (13), **f)** clear lake model exponential function eq. (16). Details about the linear regressions and merits-of-fits are specified in Table S6.

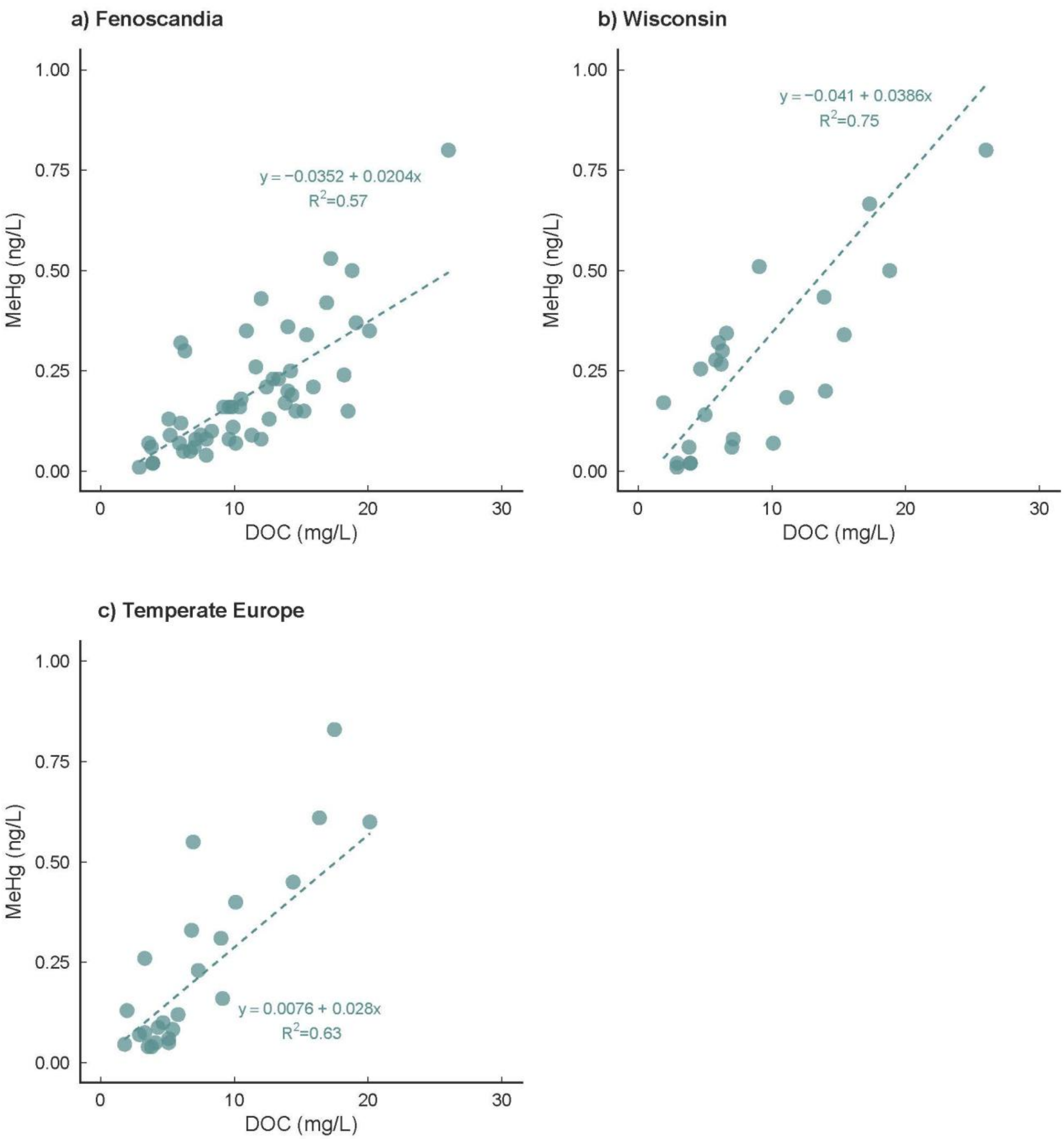

Fig. S12. a) Relationship between dissolved organic carbon (DOC, mg L^{-1}) and concentrations of MeHg (ng L^{-1}) in 54 lakes from Norway, Sweden and Finland, used to calculate concentrations of MeHg in the 1033 Swedish lakes.[21-29] **b)** Relationship between DOC (mg L^{-1}) and concentrations of MeHg (ng L^{-1}) in 24 lakes from Wisconsin, USA.[30-33] **c)** Relationship between DOC (mg L^{-1}) and concentrations of MeHg (ng L^{-1}) in 25 lakes from Temperate Europe[22,24-27] complemented by data from the present study.

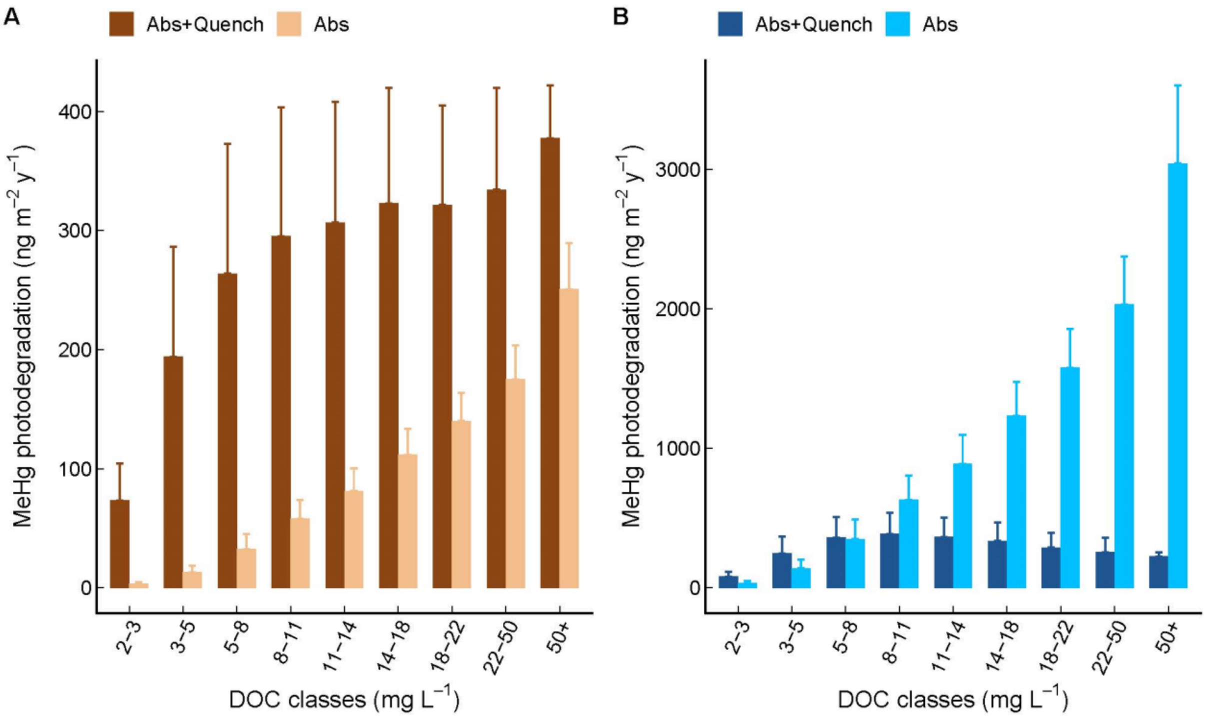

Fig. S13. MeHg photodegradation modeled for 1033 Swedish lakes sorted into DOC concentration classes. **A** Dark lake model. Dark brown bars eq. (13) and light brown bars eq. (7). **B** Clear lake model. Dark blue bars eq. (16) and light blue bars eq. (10). Dark colored bars represent the AQY_{quench} model with the quenching term ($a$exp(b/(1+UVA/UVA$_{1/2}$)) included. Light colored bars represent the AQY model without the quenching term. Notably, the two models give very different results without inclusion of the quenching term. Inclusion of the quenching term makes the model outputs from the two models quite similar (pay notation to the different scales of the y-axis), albeit some systematic differences for specific DOC classes remain (c.f. Fig. 2A,B). Note that dark and light-colored bars of the same size (e.g. for DOC class 5 – 8 mg/L in clear lake model, Fig S13B) indicates that the effect of quenching is similar in the DOC class and in the lake (LJU) by which the clear lake model was derived.

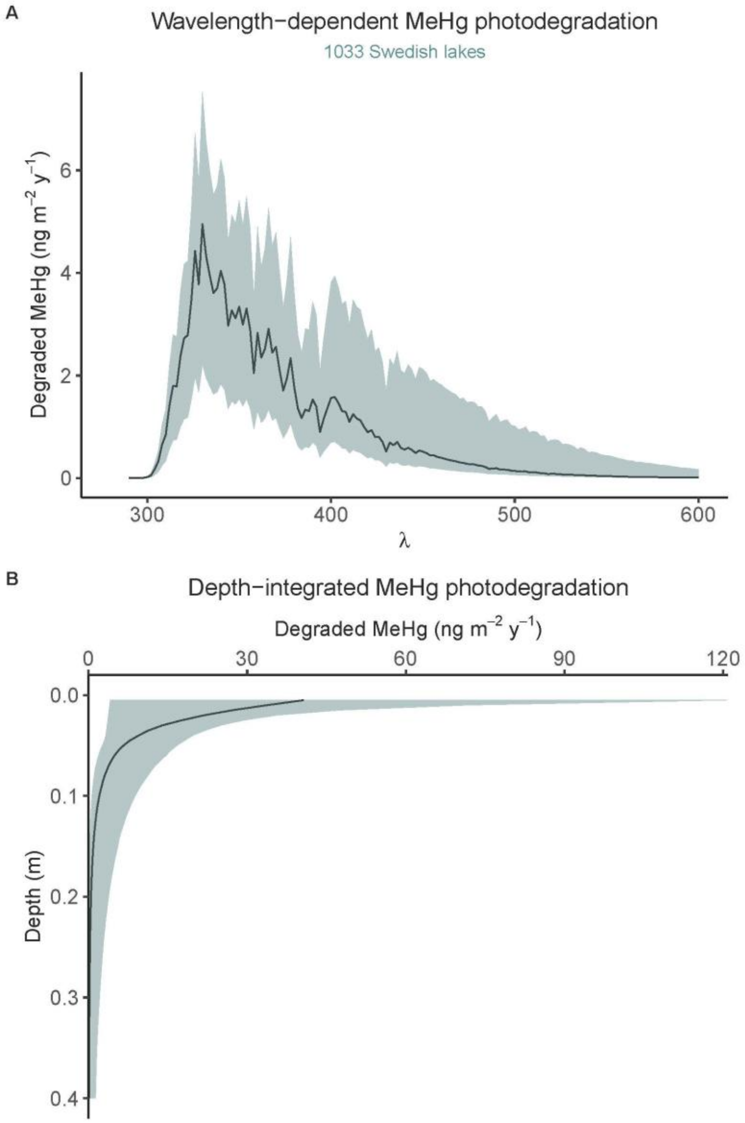

Figure S14. Spectral- and depth-dependency of MeHg photodegradation in 1033 Swedish lakes. Median, 25-% and 75-% percentiles (shading) of the MeHg photodegradation per wavelength (**A**) and per depth (**B**). The percentage contribution of MeHg photodegradation per bandwidth is: UV-B (290-309 nm): 7.7 ± 1.7%, UV-A (310-399 nm): 63.4 ± 7.0%, PAR (400-600 nm): 28.9 ± 8.7%, and total photodegradation per depth is 0-1 cm: 26.8 ± 16.5%, 0-5 cm: 64.5 ± 24.3 %, and 0-50 cm: 95.3 ± 4.4%. Calculations in (**A**) were performed using the combined dark (eq. 7, $SUVA_{254} \geq 2.5$) and clear lake (eq. 10, $SUVA_{254} < 2.5$) models, including multiplication with the quenching term $0.955exp(3.89/(1+UVA/UVA_{1/2}))$ and $0.02exp(5.25/(1+UVA/UVA_{1/2}))$ for dark and clear lakes, respectively. Calculations in (**B**) by eqs. (13) and eqs. (16) for dark and clear lakes, respectively, integrated to full spectrum per depth interval by eq. (20).

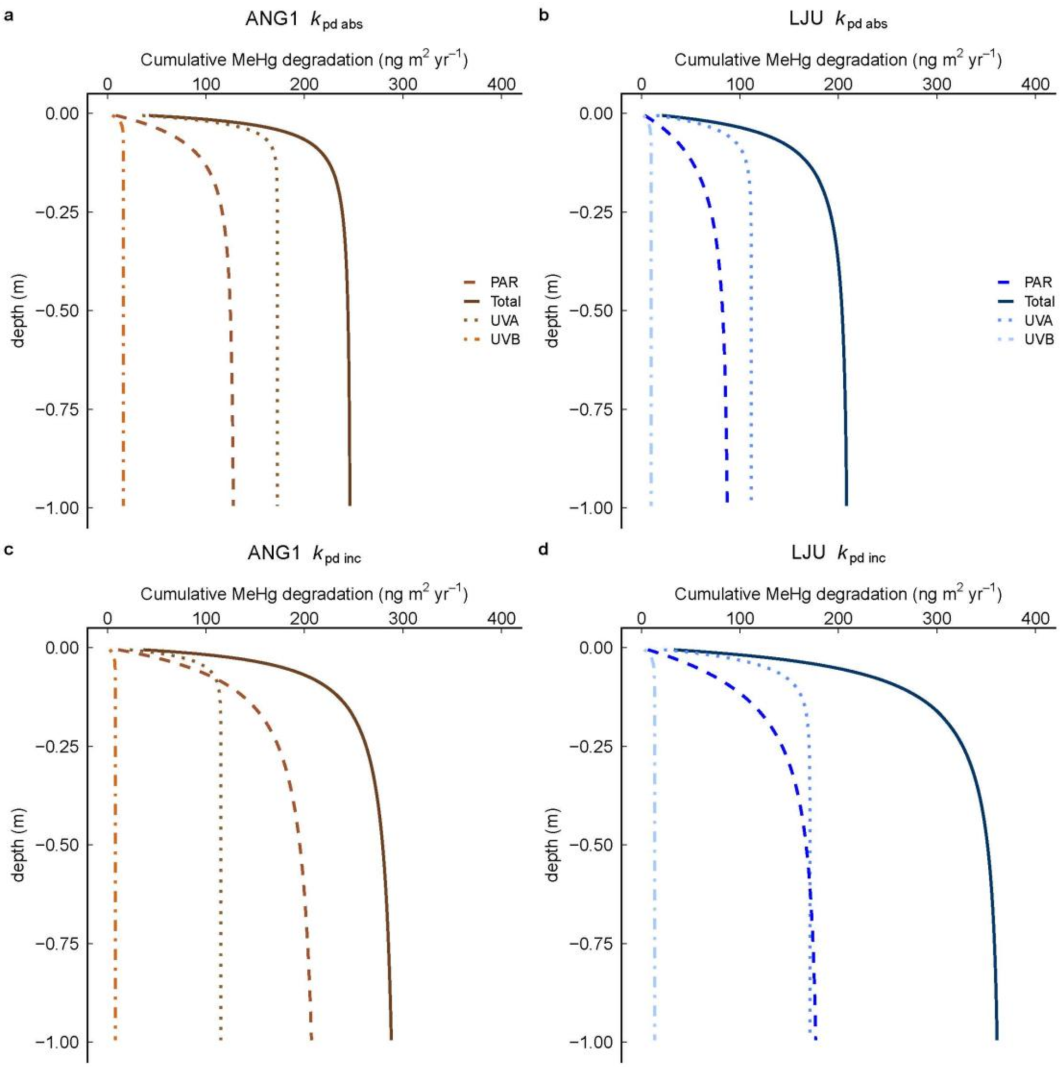

Fig. S15. MeHg photodegradation (ng m^{-2} y^{-1}) calculated by AQY ($k_{pd\ abs}$) and by incidence models ($k_{pd\ inc}$). The MeHg photodegradation is reported by depth in the dark (ANG) and the clear (LJU) lake, separated into wavebands of UV-B (250-309 nm), UV-A (310-399 nm) and PAR (400-700 nm). Models include the exponential term to cover the wavelength dependency of photon absorbance (top: calculated by eq. (S15) – ANG1, and eq. (10) – LJU) and of incident downwelling irradiation (bottom: eq. (S12) – ANG1, and eq. (S18) – LJU. Because both models were derived from experimental data obtained from the very lakes they were applied to (when calculating the integrated MeHg photodegradation by depth) all possible mechanisms (including the effect of RTS quenching) are incorporated in the model outputs. This means that the specific terms used for the quenching process should not be added to the models. Notable is that in the dark lake the UV-B and UV-A wavebands give rise to substantially larger absolute MeHg degradation by the absorbance than by the incidence model, whereas in the clear lake all three wavebands give rise to larger MeHg degradation by the incidence model. In both lakes PAR makes a larger contribution to MeHg degradation in the incidence model, than in the absorbance model. Concentrations of MeHg were 0.40 ng L^{-1} and 0.02 ng L^{-1} in the dark and clear lakes, respectively, and concentrations of DOC were 16.5 and 2.9 mg L^{-1}, respectively. To keep the light conditions the same in both lakes, we used the calculated ten-year-average (2006-2015) for lake ANG. The depth integration step was 0.005 m in both lakes.

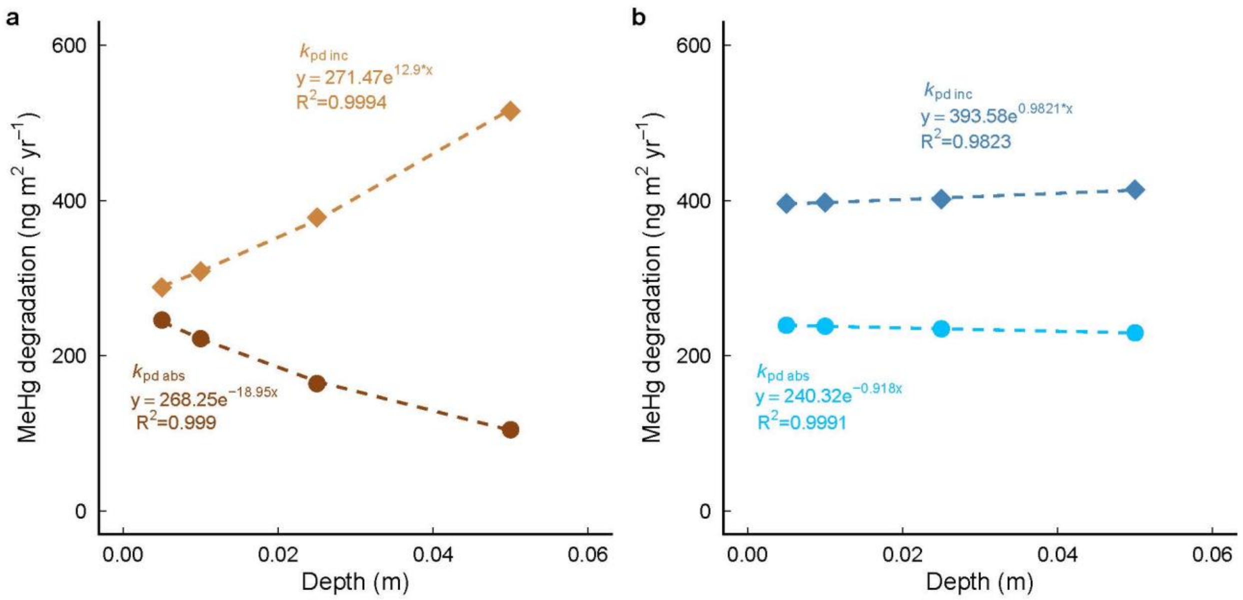

Fig. S16. Comparison of the outcome when applying models based on absorbed or incident irradiance in the dark (ANG) and a clear lake (LJU), as a function of lake integration depth-intervals. In the dark lake the integration interval is crucial for the result and if small enough, the two models (absorbance and incident radiation) give similar results (268 and 271 ng m^{-2} yr^{-1}, respectively), even if the spectral contribution is very different (**Fig. S15**). In the clear lake (LJU) the integration depth-interval is less important for the result and the MeHg degradation is always much higher when calculated by the incidence model (average 393 ng m^{-2} y^{-1}) than by the absorbance model (average 240 ng m^{-2} y^{-1}). Concentrations of MeHg were 0.40 ng L^{-1} and 0.02 ng L^{-1} in the dark and clear lake, respectively, and concentrations of DOC were 16.5 and 2.9 mg L^{-1}, respectively. The light conditions were the same in both lakes, calculated as ten-year-average (2006-2015) for lake ANG.

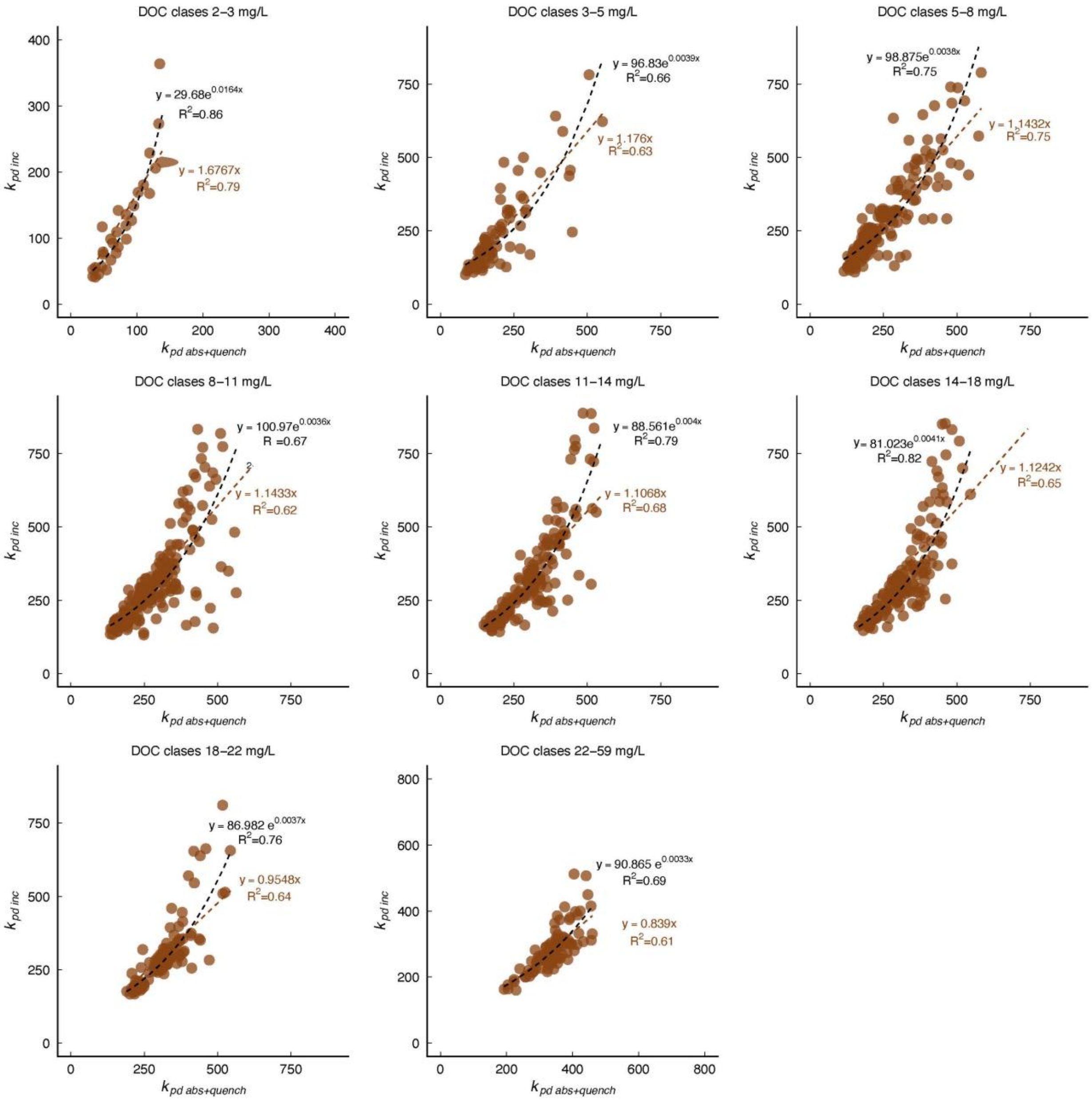

Fig. S17. Data on MeHg degradation (ng m^{-2} y^{-1}) in the data set of 1033 Swedish lakes calculated by incident ($k_{pd\ inci}$, eq. S12, y-axis) and AQY ($k_{pd\ abs+quenc}$, eq. S15, x-axis) dark lake models (derived from lake ANG1) divided into DOC classes. Exponential relationships in black and linear regression in brown. Slopes of the latter above or below unity demonstrate an overestimate and underestimate, respectively, of MeHg photodegradation by the incidence model.

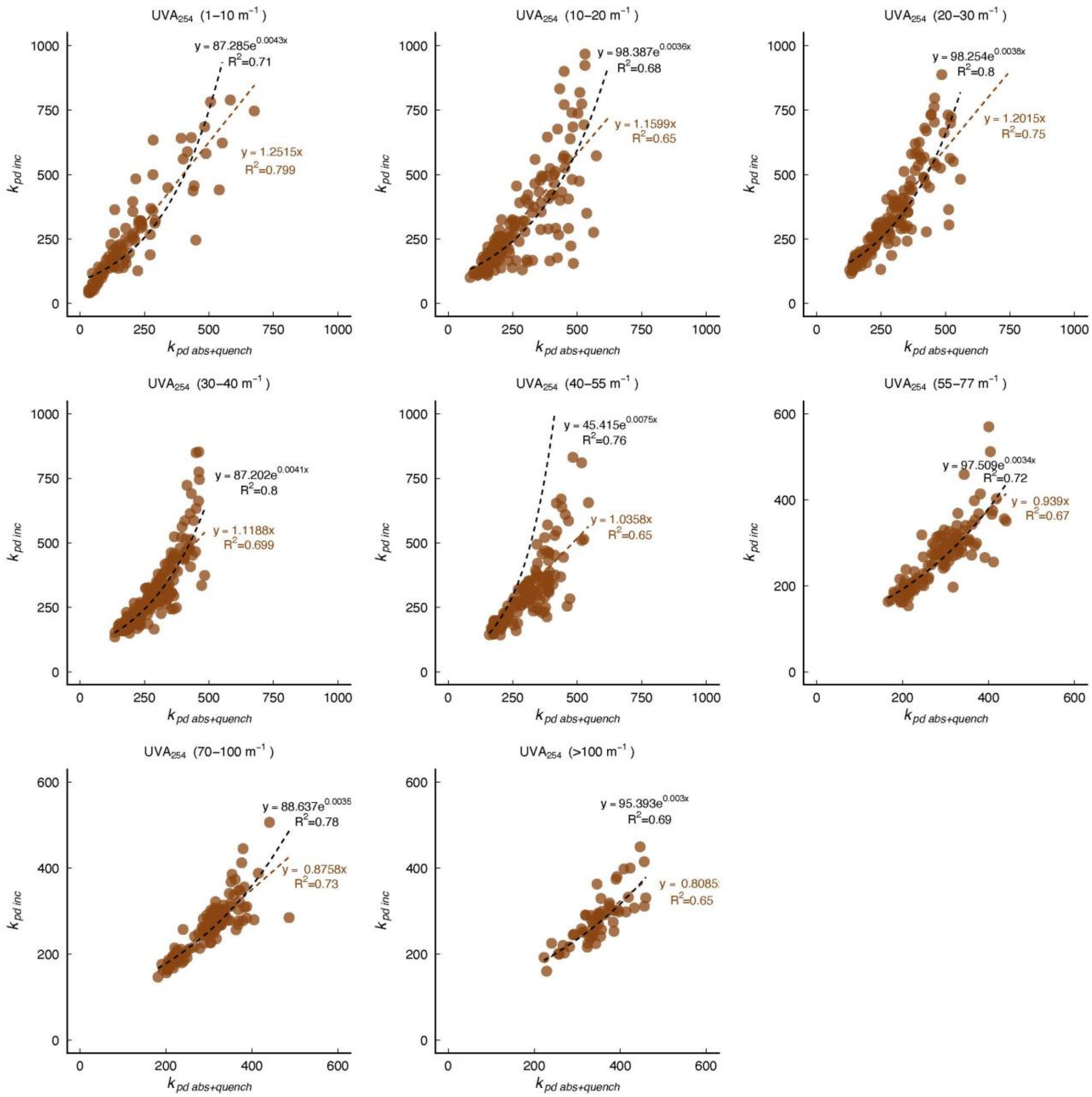

Fig. S18. Data on MeHg degradation (ng m^{-2} y^{-1}) in the data set of 1033 Swedish lakes calculated by incident ($k_{pd\ inci}$, eq. S12, y-axis) and AQY ($k_{pd\ abs+quenc}$, eq. S15, x-axis) dark lake models (derived from lake ANG1) divided into UVA_{254} classes. Exponential relationships in black and linear regression in brown. Slopes of the latter above or below unity demonstrate an overestimate and underestimate, respectively, of MeHg photodegradation by the incidence model.

Supplementary Tables S1-S12

Table S1 – Selected chemical data of the three experimental lakes LJU, LS, ANG
Table S2 - Linear regressions and merit-of-fits for AQY models derived for dark (LS+ANG12) and clear (LJU) lakes
Table S2 – Chemical characteristics and experimentally determined rate constants for MeHg photodegradation in 25 globally distributed lakes
Table S3 – Ancillary chemistry for the 25 lakes for which experimental data on MeHg photodegradation were determined.
Table S4 – A summary of the MeHg photodegradation models and constants derived from three experiemntal data sets in this work
Table S6 - Linear regressions and merit-of-fits for AQY_{quench} models applied to clear and dark lakes
Table S7 – Summary of lake water chemistry for the set of 1033 Swedish lakes
Table S8 – Annual rates of MeHg photodegradation calculated for lakes in for Sweden, United Kingdom, and for the global regions of South Boreal Scandinavia, Temperate Europe, Temperate North America, Sub-tropical North America and Tropical South America
Table S9 – Rates of MeHg photodegradation in lakes reported in literature
Table S10 – Rates of MeHg photodegradation calculated for Toolik lake, Alaska. Comparison with literature data.
Table S11 – Rates of MeHg photodegradation calculated for 1033 Swedish lakes by use of incident radiation models
Table S12a – Selected data and rates of MeHg photodegradation calculated for 119 Wisconsin lakes
Table S12b – Selected data and rates of MeHg photodegradation calculated for 23 UK lakes
Table S12c – Data on MeHg concentrations collected in wet and dry seasons of 2005 at stations in Florida Everglades
Table S12d – Selected data and rates of MeHg photodegradation calculated for Florida Everglades

Table S1. Characteristics and selected chemical properties of the three contrasting lakes (LJU, LS and ANG) for which experimental data were obtained to derive the spectral AQY models. Data from ANG includes data at one sampling occasion in February 2014 analyzed after different storage time (ANG1 – immediately measured and ANG2 – stored for 1 month. Data from LJU and LS is from one sampling occasion in summer 2014. Nitrate concentrations were below the detection limit of 0.1 mg L^{-1}. Decadic $SUVA_{254}$ was corrected for the absorbance by iron, following Weishaar *et al.* [34]

**Lake**	**Lake area** (km^2)	**Maximum lake depth** (m)	**MeHg** ng L^{-1}	**Fe** mg L^{-1}	**pH**	**DOC** mg L^{-1}	**$SUVA_{254}$** L mg^{-1} m^{-1}	**Fe-corrected $SUVA_{254}$** L mg^{-1} m^{-1}
Ljustjärn (LJU)	0.12	11	0.02	<0,1	5.9	2.9	1.8	1.8
Lilla Sångaren (LS)	0.24	17	0.50	<0,1	5.8	9.1	4.2	4.2
Ängessjön (ANG12)	0.70	3	1.71	4.8	4.8	39.5	4.9	3.7

Table S2. Linear regressions ($y = ax + b$) established between data on experimentally determined AQY_{exp} for the five cut-off filters ($\Phi_{250\text{-}700}$, $\Phi_{310\text{-}700}$, $\Phi_{351\text{-}700}$, $\Phi_{381\text{-}700}$, and $\Phi_{421\text{-}700}$) and AQY_{model} ($\Phi_{250\text{-}700}$, $\Phi_{310\text{-}700}$, $\Phi_{351\text{-}700}$, $\Phi_{381\text{-}700}$, and $\Phi_{421\text{-}700}$) (x). These relationships are illustrated in **Fig. S4** (dark lakes) and **Fig S5** (clear lake).

	Models	y = *a*x + *b*	R^2	Merit-of-fit (eq. 4)
	LS+ANG12 dark lake model			
AQY	Power function, eq. (5)	*y*=0.9999*x* – 0.00009	0.926	0.037
AQY	Vähätalo function, eq. (6)	*y*=0.9996x - 0.00001	0.929	0.039
AQY	Exponential function, eq. (7)	*y*=0.9999*x* - 0.00005	0.945	0.024
	LJU clear lake model			
AQY	Power function, eq. (8)	*y*=1.0052 - 0.00009	0.925	0.019
AQY	Vähätalo function, eq. 9)	*y*=1.0002*x* - 0.0001	0.925	0.020
AQY	Exponential functio, eq. (10)	*y*=0.9995x - 0.0028	0.924	0.048

Table S3. Three types of mathematical functions, eqs. (1), (2), and (3), were incorporated in the models describe the spectral dependence of MeHg photodegradation data in the clear lake (LJU), the two dark lakes (ANG12+LS) and in the dark end-member lake (ANG1). For all three data sets, spectral AQY_{λ} models were developed. To be able to compare with previously reported results in the literature, using models based on incidence irradiation, we also calculated the photodegradation rate constant $k_{pd\ (\lambda)\ inci}$ for the clear (LJU) and the very dark end-member lake (ANG1).

Wavelength functions	Power eq. (1) $a\lambda^{-b}$	Vähätalo eq. (2) $a10^{-b\lambda}$	Exponential eq. (3) $exp^{-(m1+m(2\lambda-290))}$
Experimental data sets			
LJU (clear lake)	$k_{pd\ (\lambda)\ inci}$ AQY_{λ}	$k_{pd\ (\lambda)\ inci}$, AQY_{λ}	$k_{pd\ (\lambda)\ inci}$, AQY_{λ}
ANG12+LS (dark lakes)	AQY_{λ}	AQY_{λ}	AQY_{λ}
ANG1 (dark end-member lake)	$k_{pd\ (\lambda)\ inci}$, AQY_{λ}	$k_{pd\ (\lambda)\ inci}$, AQY_{λ}	$k_{pd\ (\lambda)\ inci}$ AQY_{λ}

Table S4. Geographic coordinates, physical and chemical characteristics, experimentally determined MeHg photodegradation rate constants $k_{pd\ inci}$, $k_{pd\ abs}$ and apparent quantum yield (AQY, by eqs. S1-S5) for the 25 globally distributed lakes (**Fig. S7**). Decadic $SUVA_{254}$ was corrected for the absorbance by iron, following Weishaar *et al.*[34] EC = electric conductivity, NA = Data not available.

**Lake/Reservoir**	**Latitude**	**Longitude**	**Depth (m) (m)**	**MeHg (ng L^{-1})**		**k_{pd} (E m^{-2})**		**AQY**	**EC**	**pH**	**DOC**	**$SUVA_{254}$**
**Temperate Europe**			(z max)	mean	SD	Inci	Abs	nmol E^{-1}	µS cm^{-1}		mg L^{-1}	L mg^{-1} C m^{-1}
Stechlin (GE)	53°09'06"N	13°01'34"E	68	0.141	0.01	0.0039	0.248	0.072	301	7.2	5.0	1.8
Grosse Fuchskuhle (GE)	53°06'21"N	12°59'05"E	4	0.666	0.05	0.0049	0.026	0.008	31	5.7	17.3	3.3
Melzer see (GE)	53°31'38"N	12°42'12"E	3	0.184	0.01	0.0050	0.110	0.036	774	7.6	11.3	1.8
Elvåga (NO)	59°52'46"N	10°54'35"E	23	0.277	0.02	0.0045	0.049	0.014	18	5.7	5.8	4.1
Solbergvann (NO)	59°53'60"N	10°51'59"E	5	0.434	0.02	0.0032	0.016	0.005	15	4.8	13.9	4.1
Svartjärn (SE)	59°53'27"N	15°15'28"E	7	0.506	0.02	0.0034	0.008	0.002	25	5.0	36.3	5.7
Ljustjärn (SE)	59°55'26"N	15°27'12"E	11	0.020	NA	0.0057	0.234	0.027	14	5.9	2.9	1.8
Lilla Sångaren (SE)	59°53'59"N	15°23'32"E	17	0.510	0.03	0.0035	0.020	0.002	37	5.8	9.0	4.2
Ängesjön (SE)	64°02'36"N	20°50'12"E	3	1.707	0.95	0.0027	0.006	0.001	62	4.8	39.5	3.7
Feeagh (IR)	53°56'49"N	9°34'26"W	45	0.344	0.03	0.0047	0.097	0.033	104	7.0	7.6	4.0
Bunaveela (IR)	54°01'17"N	9°32'44"W	10	0.267	0.02	0.0047	0.048	0.014	96	6.2	6.2	3.9
Black (IR)	53°54'48"N	9°35'04.8"W	2	0.255	0.02	0.0062	0.120	0.037	131	6.9	4.7	2.7
**Temperate N America**												
Lake Mendota (WI, USA)	43° 6'24"N	89 25'29"W	25	0.211	0.01	0.0053	0.306	0.097	559	8.5	7.9	1.1
Mary Lake (WI, USA)	46°15'2"N	89 54'1.2"W	20	0.922	0.11	0.0024	0.007	0.002	20	5.8	28.7	3.8
Trout Bog (WI, USA)	46°01'46"N	89°40'25"W	7	1.037	0.09	0.0035	0.014	0.004	12	5.1	20.1	3.7
**Subtropical N America**												
Monument (FL, USA)	25°52'08"N	81°06'51"W	0.4	0.312	0.01	0.0045	0.026	0.008	292	6.7	17.6	2.9
Shark lake (FL, USA)	25°45'04"N	80°45'43"W	0.5	0.610	0.04	0.0060	0.082	0.027	429	7.3	16.1	2.0
160 (FL, USA)	25°23'43"N	80°34'54"W	0.4	0.185	0.03	0.0051	0.115	0.033	606	7.4	14.5	1.7
Ent (FL, USA)	25°37'2"N	80°34'31"W	NA	NA	NA	0.0022	0.046	0.013	379	7.5	6.4	2.0
**Tropical S America**												
Funil Reservoir (BR)	22°32'18"S	44°39'21"W	22	0.290	0.04	0.0051	0.187	0.055	102	6.4	3.9	2.3
UHE Samuel (BR)	8°52'22"S	63°16'56"W	25	0.353	0.04	0.0045	0.070	0.022	282	7.5	5.2	3.5
Juruena Salto Augusto (BR)	8°55'05"S	58°33'25"W	5.5	0.148	0.01	0.0041	0.087	0.026	90	7.1	4.2	2.8
Marfil Lake (BR)	15°31'07"S	60°13'26"W	3	0.147	0.01	0.0033	0.059	0.019	112	7.0	5.4	2.9
Siá Mariana Lake (BR)	16°17'37" S	55°52'34"W	8.5	0.144	0.02	0.0046	0.165	0.048	54	6.5	2.4	2.7
Baía Grande Lake (BR)	16°38'06"S	57°29'00"W	12.6	0.168	0.01	0.0042	0.126	0.04	59	6.9	1.8	3.0

Table S5. Selected ancillary chemistry determined in the water of the 25 lakes for which experimental data on MeHg photodegradation were collected. NA = Data not available.

**Lake/Reservoir**	**pH**	**EC**	**Cl**	**F**	**NO_3^-**	**PO_4^{3-}**	**SO_4^{2-}**	**NH_4^+**	**Na^+**	**Fe**	**Ca**
**Temperate Europe**		µS cm^{-1}	mg L^{-1}	mg L^{-1}	mg L^{-1}	mg L^{-1}	mg L^{-1}	mg L^{-1}	mg L^{-1}	mg L^{-1}	mg L^{-1}
Stechlin (GE)	7.2	300	14	0.12	<0.01	<0.01	37	0.01	8.2	<0.09	3.2
Grosse Fuchskuhle (GE)	5.7	31	3.7	0.09	<0.01	<0.01	1.8	<0.01	2.4	<0.09	0.54
Melzer see (GE)	7.6	770	45	0.09	<0.01	<0.01	120	0.13	20	<0.09	124
Elvåga (NO)	5.7	18	2.9	0.04	0.28	<0.01	2.7	0.02	2.1	<0.09	0.35
Solbergvann (NO)	4.8	15	2.1	0.04	0.24	<0.01	1.8	0.10	2.0	0.26	1.0
Svartjärn (SE)	5.0	25	4.7	0.05	0.24	<0.01	2.4	0.19	3.1	0.53	0.31
Ljustjärn (SE)	5.9	14	2.0	0.06	0.52	<0.01	1.9	<0.01	1.4	<0.09	0.41
Lilla Sångaren (SE)	5.8	37	NA	NA	NA	NA	NA	NA	NA	NA	NA
Ängesjön (SE)	4.8	62	6.8	0.09	0.05	<0.01	8.6	0.04	5.6	4.8	1.5
Feeagh (IR)	7.0	100	22	0.01	0.17	<0.01	3.2	0.04	12	<0.09	2.7
Bunaveela (IR)	6.2	96	21	0.02	0.01	<0.01	2.7	<0.01	12	0.13	2.8
Black (IR)	6.9	130	29	0.02	<0.01	<0.01	4.4	0.08	10	<0.09	1.3
**Temperate N America**											
Lake Mendota (WI, USA)	8.5	560	52	0.06	0.38	<0.01	20	0.26	28	<0.09	37
Mary Lake (WI, USA)	5.8	20	0.7	0.03	<0.01	<0.01	1.1	0.08	1.4	0.23	3.6
Trout Bog (WI, USA)	5.1	12	0.3	0.03	<0.01	<0.01	0.51	0.02	0.73	0.30	1.1
**Subtropical N America**											
Monument (FL, USA)	6.7	290	8.8	0.03	0.24	<0.01	0.65	0.04	6.4	<0.09	62
Shark lake (FL, USA)	7.3	430	28	0.10	0.02	<0.01	7.0	0.11	20	<0.09	68
160 (FL, USA)	7.4	610	56	0.08	0.18	<0.01	0.15	0.39	37	<0.09	89
Ent (FL, USA)	7.5	NA	NA	NA	NA	NA	NA	NA	NA	NA	NA
**Tropical S America**											
Funil Reservoir (BR)	6.4	100	6.6	0.10	1.9	<0.01	12	0.08	10	<0.09	1.3
UHE Samuel (BR)	7.5	280	3.2	0.06	0.04	<0.01	2.8	0.14	6.4	<0.09	25
Juruena Salto Augusto	7.1	90	1.1	0.08	0.07	<0.01	0.22	0.08	4.0	<0.09	8.4
Marfil Lake (BR)	7.0	110	1.9	0.06	0.01	<0.01	0.14	0.04	3.9	<0.09	11
Siá Mariana Lake (BR)	6.5	54	0.7	0.04	0.44	<0.01	0.42	0.04	2.2	<0.09	5.4
Baía Grande Lake (BR)	6.9	59	0.6	0.05	0.25	<0.01	0.84	<0.01	2.8	<0.09	5.6

Table S6. Linear regressions ($y = ax + b$) established between data on experimentally determined AQY_{exp} ($\Sigma\Phi_{250\text{-}700}$) and $k_{abs\ exp}$ (y) and modeled AQY_{quench} ($\Sigma\Phi_{250\text{-}700}$) and $k_{abs\ quench}$ (x) for 25 globally distributed lakes. The average and range of the inhibitory factor (quenching), as represented by the term $a\exp(b/(1+UVA/UVA_{1/2}))$, are calculated for the 25 global lakes. The size of this parameter suggests that the DOM inhibition effect in lake LJU (0.85-0.98, e.g. below unity) is slightly less (85-98 %) than the average in the 22 globally distributed lakes. In stark contrast, the quenching term in the dark lakes LS+ANG12 is 8 times (810-840%) higher than the average in the 22 globally distributed lakes. Residual standard error of regression (RSE) has the unit $m^2\ E^{-1}$ for $k_{pd\ abs\ quench}$ and the unit $nmol\ E^{-1}$ for the term AQY_{quench}.

	Models	y = *a*x + *b*	R^2	RSE	Merit-of-fit (eq. 4)	Average and range of quenching term
	LS+ANG12 dark lake model					
AQY_{quench}	Power function (eq.11)	*y*=0.9999*x* - 0.00001	0.724	0.0126	0.122	8.1 (1.3-20.2)
AQY_{quench}	Vähätalo function (eq. 12)	*y*=1.0008*x* - 0.00001	0.730	0.0125	0.120	8.4 (1.4-20.8)
AQY_{quench}	Exponential function (eq. 13)	*y*=0.9999*x* - 0.00005	0.752	0.0122	0.115	8.4 (1.4-20.9)
$k_{pd\ abs+quench}$	Exponential function (eq. 17)	*y*=0.9998 - 0.0052	0.839	0.034	0.073	8.4 (1.4-20.9)
	LJU clear lake model					
AQY_{quench}	Power function (eq. 14)	*y*=0.9999*x* - 0.00001	0.719	0.0128	0.125	0.88 (0.06-2.1)
AQY_{quench}	Vähätalo function (eq. 15)	*y*=1.0001*x* - 0.00001	0.716	0.0128	0.126	0.85 (0.05-2.0)
AQY_{quench}	Exponential function (eq. 16)	*y*=1.0009*x* - 0.00001	0.730	0.0125	0.120	0.98 (0.06-2.3)
$k_{pd\ abs+quench}$	Exponential function (eq. 18)	*y*=0.9964 - 0.0044	0.809	0.037	0.086	0.98 (0.06-2.3)

Table S7. Summarized physical and chemical characteristics of the data set of 1033 Swedish lakes.[35]

Lake characteristics	Mean ± SE	Range	Median
Area (km^2)	1.46±0.24	0.00007-158.5	0.13
Mean depth (m)[1]	3.8±0.08	0.5-38.7	3.2
Volume (1000 m^3)	15980±6040	20-6140000	499
Ice breakup (day of year)[2]	122±1	55-179	118
Ice duration (days per year)[2]	133±1	0-247	125
pH[3]	6.6±0.02	4.2-8.3	6.7
Fe-corrected $SUVA_{254\ nm}$[3]	3.1±0.03	0.60 – 5.0	3.2
Nitrate (µg L^{-1})[3]	45±3.7	1-2880	18
Total iron (µg L^{-1})[3]	640±22	10-5500	410
Total phosphorus (µg L^{-1})[3]	16.8±0.67	1-395	11.0
Total nitrogen (µg L^{-1})[3]	487±9.8	51-3500	412
Total organic carbon (mg L^{-1})[3]	13.3±0.23	1.9-50.3	12.0

[1]Mean depth for lakes >0.1 km^2 in area (n= 251) was available from the Swedish lake register of the Swedish Meteorological and Hydrological Institute. For lakes 0.01–0.1 km^2 area (n = 771) mean depth was calculated according to Sobek et al., 2011.[36] For the remaining lakes <0.01km^2 (n = 64), mean depth was assigned to the median depth of the lakes from 0.01 to 0.015 km^2 area (2.3 m).[7]

[2]Long-term average (1961–1990) ice breakup and ice duration for each lake was calculated based on an arc-cosine air temperature function[37] using air temperatures from the Swedish Meteorological and Hydrological Institute.[7]

[3]Water chemical data from the Swedish National Lake Inventory 2009 are available at the "Data host for inland waters" from the Swedish University of Agricultural Sciences (http://info1.ma.slu.se/ri/www_ri.acgi$Project?ID=2009KS, in Swedish). $SUVA_{254\ nm}$ corrected for Fe-absorbance following Weishaar *et al.*[34]

Table S8. Calculated, annual rates of MeHg photodegradation (mean ± SE) upscaled for lakes in Sweden, UK and five "global regions" (South-Boreal Scandinavia, Temperate Europe, Temperate North America, Subtropical North America and Tropical South America). Besides the spectral absorbance in lakes (not reported here), important input parameters for the AQY model and upscaling (lake mean depth, MeHg concentration, annual solar irradiance and $SUVA_{254}$) are reported as mean ± SD. Solar radiation is above the water surface (spectral range 280 – 600 nm) calculated as annual average for the period 2005-2016. The dark lake model was applied to lakes with Fe-corrected $SUVA_{254} \geq 2.0$ L m^{-1} mg^{-1} and the clear lake model was applied to lakes with $SUVA_{254} < 2.0$ L m^{-1} mg^{-1}. *Denotes data from 23 of the experimental lakes divided into five regions and four data sets represent upscaling for lake water data sets from Sweden (Boreal Europe), UK (Temperate Europe), Wisconsin (Temperate North America) and Florida Everglades (Subtropical North America).

Region	Latitudal range (N)	No. (lakes)	Mean depth (m)	MeHg (ng L^{-1})	Solar radiation (E m^{-2} y^{-1})	Number of ice-free days (y^{-1})	$SUVA_{254}$ (L m^{-1} mg^{-1})	DOC (mg L^{-1})	pH	MeHg photo-degradation (ng m^{-2} y^{-1})
Sweden	55˚05'-68˚08'	1033	3.8 ± 2.5	0.24 ± 0.15[1]	3340 ± 817	232 ± 1	3.1 ± 0.84	13.3 ± 7.5	6.6 ± 0.7	320 ± 4.5
*S Boreal Scandinavia	59˚52'-59˚55'	5	12 ± 20	0.35 ± 0.21	4751 ± 11	240	4.0 ± 1.5	12.1 ± 10	5.4 ± 0.5	470 ± 130
*Temperate Europe (Ireland & Germany)	53˚06'-54˚01'	6	22 ± 28	0.31 ± 0.19	5787 ± 146	300	2.9 ± 1.0	8.7 ± 4.9	6.8 ± 0.7	910 ± 110
United Kingdom	52˚48'-54˚47'	23	4.3 ± 2.3	0.22 ± 0.17[2]	5695 ± 150	300	2.5 ± 0.70	7.7 ± 6.1	NA	800 ± 74
*Temperate N America (Wisconsin)	43˚06'-46˚01'	3	17 ± 9.3	0.72 ± 0.45	6644 ± 197	240	2.9 ± 1.5	18.9 ± 10	6.5 ± 1.8	1310 ± 320
Temperate N America (Wisconsin)	45˚60'-46˚30'	119	4.4 ± 2.5	0.24 ± 0.12[3]	6711 ± 197	240	2.2 ± 0.70	7.2 ± 3.1	6.1 ± 0.7[3]	1180 ± 45
*Subtropical N America (Florida Everglades)	25˚23'-25˚52'	3	0.43 ± 0.06	0.37 ± 0.22	11244 ± 231	365	2.2 ± 0.63	16.1 ± 1.6	7.1 ± 0.4	1410 ± 630
Subtropical N America (Florida Everglades)	25˚20'-26˚60'	118	0.41 ± 0.26[5]	0.39 ± 0.47[4]	11244 ± 231	365	2.2 ± 0.63	18.5 ± 7.8	7.3 ± 0.4	1510 ± 35
*Tropical S America	8˚52'S-22˚32'S	6	13 ± 9.0	0.21 ± 0.09	11334 ± 243	365	2.9 ± 0.39	3.8 ± 1.5	6.9 ± 0.4	2820 ± 520

Average ± SD calculated from DOC based on the linear relationships with MeHg for 54 Fennoscandian lakes (F**ig. S12a**), 25 Temperate European lakes (**Fig. S12c**), and 24 Wisconsin lakes (**Fig. S12b**). Average concentrations of MeHg from Florida Everglades from sampling in May 2005 (0.45 ± 0.29), representing dry season (Nov-Apr), and in Nov 2005 (0.29 ± 0.16 ng L^{-1}), representing the wet season (**Table S12c,d**). Mean water depth is 0.21 ± 0.20 m in dry and 0.60 ± 0.30 m in wet season. NA = Not available.

Table S9. Reported rates of MeHg degradation in lakes from *In Situ* field incubation studies. Units: decadic $SUVA_{254}$ L mg^{-1} $m^{-1,}$ DOC mg L^{-1}, MeHg ng L^{-1}. NA = Not available.

Lake	K_d (m^{-1}) naperian	$k_{pd\ inci}$ (10^{-3} m^2 E^{-1}) *(expressed in relation to measured PAR)	Incident sunlight E m^{-2} y^{-1}	MeHg degradation in lake	Remark	Reference
ELA L240, CA (pH 6.5-7.3, SUVA NA, DOC 7, MeHg 0.04 ng L^{-1})	UVB = 14<br>UVA = 5.4<br>PAR = 0.57	No data	4775 PAR (191 ice-free days)	195 ng m^{-2} y^{-1}<br>1.0 ng m^{-2} d^{-1}	Empirical calculation from surface incubations in lake by exponential decay to 50 cm. MeHg photodegradation corresponding to 106% of lake input. K_d, calculated from relationships with DOC[89]	*38*
Spring Lake, MN (pH 5.8, DOC 11.0, $SUVA_{254}$ 2.7, MeHg 0.07)	UVB, UVA, PAR from Morrison et al., 1995	No data	(270 ice-free days) Mar-Nov 2003	730 ng m^{-2} y^{-1}<br>2.7 ng m^{-2} d^{-1}	Empirical calculation from 3 cm depth incubations in lake by exponential decay to 50 cm. MeHg photodegradation corresponding to 71% of lake input	*39*
Toolik lake, AL (pH 7.6, DOC 4.4, SUVA NA, MeHg 0.05)	305-380 nm measured in lake	Full spectrum 2.6*	6200 PAR (100 ice-free days)	1300 ng m^{-2} y^{-1}<br>13 ng m^{-2} d^{-1}	MeHg photodegradation based on PAR measurements alone. MeHg photodegradation corresponding to 80% of sediment porewater influx	*18*

Table S9 continued. Reported, experimentally determined MeHg photodegradation constants ($k_{pd\ \lambda\text{-}int\ inci}$) in lake water calculated from wavelength intervals (UVB, UVA, PAR) of incident sunlight, light attenuation (K_d) and concentrations of MeHg. Experimental rates were upscaled to whole lake water bodies. Units: decadic $SUVA_{254}$ L mg^{-1} m^{-1} DOC mg L^{-1}, MeHg ng L^{-1}. NA = Not available.

Lake	K_d (m^{-1}) Naperian	$k_{pd\ \lambda\text{-}int\ inci}$ (10^{-3} m^2 E^{-1}) *(expressed in relation to measured PAR)	Incident sunlight E m^{-2} y^{-1}	MeHg degradation in lake	Remark	Reference
Langtjern, NO (pH 5.0, $SUVA_{254}$ 4.2, DOC 12.0, MeHg 0.08)	UVB = 32.3<br>UVA = 14.6<br>PAR = 2.11	UVB* = 0.11<br>UVA* = 2.04<br>PAR* = 1.13	5512 (197 ice-free days per year 2000-2013)	298 ng m^{-2} y^{-1}<br>1.5 ng m^{-2} d^{-1}	MeHg photodegradation corresponding to 27% of lake input.	*22*
Sognsvann, NO (pH 6.8, $SUVA_{254}$ 3.4, DOC 3.9, MeHg 0.02)	UVB = 9.85<br>UVA = 8.18<br>PAR = 0.68	UVB* = 0.41<br>UVA* = 2.04<br>PAR* = 0.74	5885 (257 ice-free days per year 2000-2013)	160 ng m^{-2} y^{-1}<br>0.62 ng m^{-2} d^{-1}		*22*
ELA L979, CA (pH NA, SUVA NA, DOC 12.8, MeHg 0.81)	UVB = 35<br>UVA = 11<br>PAR = 2.9	UVB* = 0.87<br>UVA* = 3.22<br>PAR* = 0.31	322 PAR (7 days in July 2006)	20 ng m^{-2} d^{-1}	K_d, calculated from relationships with DOC[89]	*40*
ELA L239, CA (pH 6.9-7.5, SUVA NA, DOC 6.4, MeHg 0.02)	UVB = 14<br>UVA = 5.4<br>PAR = 0.57	UVB* = 0.87<br>UVA* = 3.22<br>PAR* = 0.31	322 PAR (7 days in July 2006)	1.2 ng m^{-2} d^{-1}	K_d, calculated from relationships with DOC[89]	*40*
Florida Everglades, FL (pH NA, SUVA NA, DOC 16.5-22.9 MeHg 0.001 - 10)	UVB =0.415DOC$^{1.86}$<br>UVA=0.299DOC$^{1.53}$	UVB* = 2.45<br>UVA* = 11.2<br>PAR* = 0	365 ice-free days in year 2005	1240 ng m^{-2} y^{-1}<br>3.4 ng m^{-2} d^{-1}	MeHg photodegradation corresponding to 31% of sediment porewater influx. K_d, calculated from relationships with DOC[72]	*20*

Table S10. MeHg photodegradation in Toolik lake reported by Hammerschmidt and Fitzgerald,[18] and calculations by the dark and clear lake $k_{pd\ abs\ quench}$ models using MeHg concentrations (0.05 ng L^{-1}) from[18] and CDOM absorbance data sampled at 0.01, 1, 3 and 5 m depth for the period May-Aug 2010-2012 (Naperian a_{300}, a_{305},, a_{313},, a_{320},, a_{340},, a_{380}, a_{413}, m^{-1}), as reported by Cory et al.,[41] recalculated to a continuous function $a_\lambda = 2083.7exp^{-0,017\lambda}$, $R^2 = 0,9996$, n = 108). Decadic UVA_{254} was calculated to be 12.1 m^{-1} from corresponding decadic functions ($a_\lambda = 904.9\ exp^{-0,017\lambda}$, $R^2 = 0,9996$, n = 108). Upscaling to whole lake following (*28*), (integration steps of 0.5 cm down to 1 m and 5 cm down to a total of 6 m) was calculated by eq. (20) and (21). Sunlight PAR = 62 E m^{-2} d^{-1}, as reported by (*18*), was recalculated to full spectrum (280 – 700 nm) for the latitude of Toolik lake.

	Results reported by Hammerschmidt and Fitzgerald (ref *18*), calculated from data on incident PAR<br>$K_d = 0.65\ m^{-1}$, $k_{pd} = 0.0026\ m^2\ E^{-1}$	Recalculation using data on PAR reported by Hammerschmidt and Fitzgerald (*ref 18*).<br>$K_d = 0.65\ m^{-1}$, $k_{pd} = 0.0026\ m^2\ E^{-1}$	Calculation of $k_{pd\ abs+quench}$ by dark lake model eq. (17)<br>$K_d = a_\lambda = 2083,7exp^{-0,017\lambda}$	Calculation of $k_{pd\ abs+quench}$ by clear lake model eq. (18)<br>$K_d = a_\lambda = 2083,7exp^{-0,017\lambda}$
1 day in Jun/Jul	13 ng m^{-2} d^{-1}	12.3 ng m^{-2} d^{-1}	2.7 ng m^{-2} d^{-1}	3.6 ng m^{-2} d^{-1}
100 ice-free days	1 300 ng m^{-2} y^{-1}	1 226 ng m^{-2} y^{-1}	268 ng m^{-2} y^{-1}	359 ng m^{-2} y^{-1}

Table S11. Calculated median and mean (± SE) MeHg photodegradation for 1033 Swedish lakes. The outcome of the AQY ($k_{pd\ abs+quench}$) model, eq. (17), for the dark lake and eq. (18) for the clear lake model) is compared with the outcome for the the incident irradiance model ($k_{\mathrm{pd\ inci}}$), for which all three wavalength-dependent functions (exponential, Vähätalo and power) are reported.

MeHg photodegradation	$k_{pd\ abs+quench}$ Exponential Function Clear lake model (eq. 18)	$k_{pd\ abs+quench}$ Exponential Function Dark lake model (eq. 17)	$k_{\mathrm{pd\ inci}}$ Exponential Function Clear lake model (eq. S18)	$k_{\mathrm{pd\ inci}}$ Exponential Function Dark lake model (eq. S12)	$k_{\mathrm{pd\ inci}}$ Vähätalo Function Clear lake model (eq. S17)	$k_{\mathrm{pd\ inci}}$ Vähätalo Function Dark lake model (eq. S11)	$k_{\mathrm{pd\ inci}}$ Power Function Clear lake model (eq. S16)	$k_{\mathrm{pd\ inci}}$ Power Function Dark lake model (eq. S10)
Median, ng m^{-2} yr^{-1}	288	297	332	274	340	294	391	330
Mean, ng m^{-2} yr^{-1}	326 ± 4.5	293 ± 3.4	374 ± 6.5	311 ± 5.5	384 ± 6.7	330 ± 5.9	442 ± 7.7	374 ± 6.7

Table S12a. Absorbance, decadic $SUVA_{254}$ and calculated annual rates of MeHg photodegradation in 119 Wisconsin lakes (Temperate North America), using the combined dark ($SUVA_{254} \geq 2.0$) and clear lake ($SUVA_{254} < 2.0$) exponential models eqs. (13) and (16). Depth-integrated annual rates of MeHg photodegradation were calculated by eqs. (20) and (21). Concentrations of MeHg were calculated from the relationship with DOC (**Fig. S12b**). Spectral, absorbance data (not shown here) were provided by the courtesy of Corinna Gries and Paul Hanson, University of Wisconsin – Madison.

lat	lon	Country	Naperian $Abs_{254\,nm}$ (m^{-1})	DOC ($mg\ L^{-1}$)	Decadic $SUVA_{254}$ ($L\ mg^{-1}\ m^{-1}$)	MeHg ($ng\ L^{-1}$)	MeHg photodegradation ($ng\ m^{-2}\ y^{-1}$)
46,25608	-89,6067	USA	60,4	9,6	2,7	0,33	1051
46,24268	-89,1443	USA	58,1	8,9	2,8	0,30	999
46,22889	-89,156	USA	35,3	7,1	2,2	0,23	1327
46,20132	-89,4081	USA	43,9	7,1	2,7	0,23	955
46,2722	-89,6581	USA	72,1	13,2	2,4	0,47	1265
46,26372	-89,6544	USA	33,1	5,4	2,7	0,17	975
46,24905	-89,5165	USA	70,8	11,5	2,7	0,40	1181
46,24857	-89,5159	USA	72,5	10,6	3,0	0,37	1062
46,23566	-89,1698	USA	77,8	16,5	2,0	0,60	1584
46,21882	-89,2347	USA	42,8	5,9	3,2	0,19	845
46,17543	-89,0474	USA	53,4	8,4	2,8	0,28	1119
46,16898	-89,0686	USA	81,3	16,9	2,1	0,61	1619
46,02802	-88,9326	USA	31,5	5,7	2,4	0,18	1166
46,02852	-89,004	USA	72,0	13,0	2,4	0,46	1166
45,94335	-89,0638	USA	7,7	3,4	1,0	0,09	1643
45,79066	-89,3171	USA	19,6	4,5	1,9	0,13	1697
45,8227	-89,5452	USA	8,0	3,8	0,9	0,10	1731
45,95527	-89,4411	USA	66,5	9,6	3,0	0,33	1027
46,17195	-89,3129	USA	25,2	5,1	2,1	0,16	1138
45,92873	-89,6016	USA	21,9	5,4	1,8	0,17	2770
45,74642	-89,6643	USA	39,2	8,0	2,1	0,27	1529
45,94173	-89,1506	USA	11,3	4,1	1,2	0,12	2228

45,81732	-89,5819	USA	8,4	3,8	1,0	0,11	1628
45,91925	-89,742	USA	28,4	6,3	2,0	0,20	1292
46,08828	-89,2675	USA	8,8	3,8	1,0	0,11	2024
45,95258	-89,5309	USA	19,0	5,8	1,4	0,18	2474
46,17201	-89,293	USA	20,6	6,0	1,5	0,19	2649
45,80338	-89,618	USA	16,5	4,0	1,8	0,11	2395
46,17271	-89,301	USA	10,7	3,7	1,3	0,10	1614
45,58542	-89,5387	USA	17,2	4,9	1,5	0,15	2226
45,71875	-89,6044	USA	7,4	2,9	1,1	0,07	1451
45,62711	-89,4565	USA	42,5	6,8	2,7	0,22	1018
46,06407	-89,3365	USA	10,0	3,9	1,1	0,11	1764
45,64763	-89,6419	USA	20,8	6,1	1,5	0,20	1624
45,63248	-89,5796	USA	42,3	7,2	2,5	0,24	1168
45,62928	-89,5778	USA	69,9	11,9	2,5	0,42	1183
45,61491	-89,4891	USA	31,7	5,0	2,8	0,15	986
45,60632	-89,547	USA	54,0	9,6	2,5	0,33	1265
45,62238	-89,337	USA	77,7	12,7	2,7	0,45	1175
45,59595	-89,4076	USA	69,1	10,0	3,0	0,35	1064
45,70183	-89,5991	USA	68,7	12,4	2,4	0,44	1349
45,67715	-89,431	USA	57,6	7,7	3,3	0,26	894
45,66473	-89,4389	USA	56,2	9,6	2,5	0,33	1271
45,70531	-89,1731	USA	68,3	9,7	3,1	0,33	953
45,78163	-89,7061	USA	47,9	8,5	2,4	0,29	1195
45,74528	-89,5724	USA	18,7	4,7	1,7	0,14	1573
45,79075	-89,5219	USA	62,2	7,7	3,5	0,25	809
45,87082	-89,7572	USA	62,0	11,0	2,5	0,38	1344
45,87622	-89,5804	USA	38,4	6,6	2,5	0,21	1078
45,88227	-89,4159	USA	51,3	7,3	3,0	0,24	958
45,91774	-89,4276	USA	75,0	11,2	2,9	0,39	1044
46,01413	-89,4176	USA	20,1	5,0	1,7	0,15	1960
45,93497	-89,3243	USA	24,7	5,4	2,0	0,17	1444

45,95014	-89,1983	USA	60,9	10,1	2,6	0,35	1205
46,04327	-89,3989	USA	61,3	9,7	2,8	0,33	1153
46,1311	-89,24	USA	50,1	9,9	2,2	0,34	1372
46,07993	-89,2424	USA	78,8	11,1	3,1	0,39	1000
45,75673	-89,4506	USA	29,9	7,6	1,7	0,25	1610
46,00703	-89,4326	USA	34,1	5,2	2,8	0,16	950
46,00645	-89,4339	USA	25,8	4,7	2,4	0,14	1043
45,84913	-89,7981	USA	16,4	4,6	1,6	0,14	1608
45,80823	-89,7389	USA	16,2	3,9	1,8	0,11	1398
45,97003	-89,7083	USA	12,9	4,0	1,4	0,11	1782
45,85053	-89,7336	USA	28,0	5,6	2,2	0,17	1246
45,58178	-89,4083	USA	46,0	7,6	2,6	0,25	1040
45,74797	-89,5854	USA	17,9	5,1	1,5	0,16	1861
45,8439	-89,3671	USA	69,6	10,3	2,9	0,36	1007
45,94892	-89,2286	USA	46,6	7,1	2,9	0,23	977
46,06592	-88,9749	USA	13,0	3,8	1,5	0,11	1758
45,86663	-89,0795	USA	12,1	3,4	1,5	0,09	1057
45,89165	-88,9905	USA	46,5	7,1	2,9	0,23	1056
45,9047	-88,9591	USA	11,7	3,4	1,5	0,09	1512
45,8022	-89,162	USA	15,4	5,3	1,3	0,16	2660
46,16087	-89,3089	USA	8,8	3,8	1,0	0,10	1832
46,14883	-89,1588	USA	33,3	7,8	1,9	0,26	1644
46,11536	-89,1595	USA	46,9	8,0	2,6	0,27	1063
46,13588	-89,4511	USA	62,3	9,3	2,9	0,32	1028
46,25667	-90,0762	USA	71,4	11,5	2,7	0,40	1094
46,12544	-90,1771	USA	33,2	6,2	2,3	0,20	1145
46,08795	-89,7068	USA	7,4	3,7	0,9	0,10	2348
46,21285	-90,124	USA	51,9	6,7	3,3	0,22	814
46,13815	-89,5379	USA	36,2	5,7	2,8	0,18	955
45,9835	-89,8188	USA	44,0	7,8	2,5	0,26	1149
46,13765	-90,1236	USA	61,2	8,0	3,3	0,27	884

45,97681	-90,1927	USA	27,1	9,6	1,2	0,33	2380
45,96125	-90,2016	USA	16,1	5,3	1,3	0,16	2226
45,92397	-89,942	USA	25,5	7,2	1,5	0,24	1719
45,97223	-89,7956	USA	11,3	5,0	1,0	0,15	1663
45,91462	-89,9512	USA	45,6	12,3	1,6	0,43	1843
46,01431	-89,9758	USA	75,1	13,8	2,4	0,49	1320
46,02775	-89,7518	USA	18,7	4,8	1,7	0,14	1741
46,001	-89,7305	USA	7,6	3,0	1,1	0,07	1185
46,00757	-89,6063	USA	63,0	8,1	3,4	0,27	853
46,08333	-90,2567	USA	44,7	7,9	2,4	0,27	1113
46,10165	-89,7092	USA	77,2	12,1	2,8	0,43	1214
46,20258	-90,0311	USA	51,1	6,6	3,4	0,21	813
46,12888	-89,4185	USA	16,7	5,0	1,5	0,15	2143
46,10805	-90,1645	USA	22,9	5,6	1,8	0,18	2257
46,18858	-90,076	USA	68,2	10,8	2,7	0,38	1050
46,25932	-89,8712	USA	73,4	11,8	2,7	0,41	1104
46,28406	-89,896	USA	50,8	9,1	2,4	0,31	1181
46,15664	-90,0888	USA	31,1	6,7	2,0	0,22	1296
46,10546	-89,8825	USA	44,4	8,3	2,3	0,28	1176
45,97928	-89,8943	USA	8,7	3,6	1,1	0,10	2516
46,03928	-89,8443	USA	15,3	4,6	1,5	0,13	2003
46,13935	-89,9063	USA	26,6	4,5	2,6	0,13	949
46,11825	-89,8288	USA	29,4	4,9	2,6	0,15	938
46,16335	-89,7835	USA	30,9	5,4	2,5	0,17	1101
46,1941	-89,6319	USA	67,2	10,9	2,7	0,38	1083
46,07545	-89,595	USA	13,0	4,4	1,3	0,13	1822
46,05737	-89,5854	USA	33,0	5,6	2,6	0,17	1008
46,25597	-89,7851	USA	67,2	11,0	2,7	0,38	1206
46,161	-89,3634	USA	18,6	4,4	1,9	0,13	1577
46,21819	-89,607	USA	70,2	10,5	2,9	0,36	1028
46,18662	-89,4573	USA	43,9	7,6	2,5	0,25	1107

Table S12b. Absorbance, SUVA $_{254\ nm}$ and calculated annual rates of MeHg photodegradation in 23 lakes of the UK, using the combined dark (SUVA ≥ 2.0) and clear lake (SUVA<2.0) exponential function models eqs. (13) and (16). Depth-integrated annual rates of MeHg photodegradation were calculated by eqs. (20) and (21). Concentrations of MeHg were calculated from the relationship with DOC (**Fig. S12c**). Spectral absorbance data (not shown here) were provided by the courtesy of Jessica Adams (UK Centre for Ecology and Hydrology, UK).

lat	lon	name	Country	Naperian Abs$_{254\ nm}$ (m^{-1})	DOC (mg L^{-1})	Decadic SUVA$_{254nm}$ (L mg^{-1} m^{-1})	MeHg (ng L^{-1})	MeHg photodegradation (ng m^{-2} y^{-1})
54,39658	-2,97661	LD1	United_Kingdom	25	3,6	0,11	3,0	594
54,44694	-2,99639	LD10	United_Kingdom	14	2,1	0,06	3,0	553
54,42791	-3,0276	LD2	United_Kingdom	12	1,7	0,05	3,0	536
54,36316	-2,98625	LD3	United_Kingdom	17	2,9	0,09	2,5	584
54,44898	-3,01924	LD4	United_Kingdom	16	2,2	0,07	3,1	475
54,36472	-2,85822	LD5	United_Kingdom	35	5,2	0,15	2,9	648
54,46551	-2,85726	LD6	United_Kingdom	15	2,2	0,07	3,0	543
54,43038	-3,01077	LD7	United_Kingdom	10	1,9	0,06	2,3	531
54,34979	-2,83127	LD8	United_Kingdom	14	2,8	0,08	2,2	704
54,37212	-2,99046	LD9	United_Kingdom	17	3,9	0,12	1,9	743
53,78272	-1,95612	PR4	United_Kingdom	93	8,9	0,26	4,5	447
52,7816	-2,33798	SCM1	United_Kingdom	210	27,7	0,79	3,3	652
53,21927	-2,59303	SCM10	United_Kingdom	28	7,4	0,22	1,6	1553
53,21478	-2,59297	SCM11	United_Kingdom	28	7,8	0,23	1,6	1462
52,98977	-2,64798	SCM12	United_Kingdom	94	16,7	0,48	2,4	742
53,29081	-2,47701	SCM13	United_Kingdom	44	7,4	0,22	2,6	766
53,2421	-2,23072	SCM14	United_Kingdom	43	7,5	0,22	2,5	827
53,29372	-2,42003	SCM15	United_Kingdom	61	11,5	0,33	2,3	790
52,89046	-2,85606	SCM17	United_Kingdom	24	6,2	0,18	1,7	1614
53,29081	-2,47701	SCM3	United_Kingdom	40	7,7	0,22	2,3	847
53,2393	-2,28015	SCM4	United_Kingdom	32	8,4	0,24	1,7	1426
52,89414	-2,84425	SCM5	United_Kingdom	56	10,7	0,31	2,3	810
53,24583	-2,67132	SCM8	United_Kingdom	142	20,1	0,58	3,1	656

Table S12c. Concentrations of MeHg in water sampled during wet and dry season at stations in the Florida Everglades. Data (collected in the studies of refs *20, 42*) were provided by courtesy of Yong Cai, Florida International University.

Florida Everglades May 2005 (dry season)		Florida Everglades November 2005 (wet season)	
STATION	MeHg (ng L^{-1})	STATION	MeHg (ng L^{-1})
370		489	0,74
365	0,244	488	0,20
364		374	0,15
351	0,623	368	0,35
350	2,36	367	2,8
124		258	0,085
123		257	0,098
122		256	0,19
121	0,261	255	0,10
120	0,324	254	0,22
119	0,551	253	0,12
118	0,694	252	0,30
117	0,182	251	0,28
115	0,14	249	0,51
114	0,113	245	0,39
112		244	0,22
110	3,83	242	0,13
109		240	0,57
108		239	0,20
107	1,27	238	1,5
106	0,678	237	0,65
105	0,153	236	0,26
104	1,06	234	0,18
102	0,394	233	1,1
101	0,549	231	0,98

100	0,725	230	0,17
99	0,249	229	0,23
97	2,57	228	0,53
96	0,405	227	0,90
95	0,187	226	0,18
94	0,287	225	0,39
93	0,197	224	0,10
91	0,305	223	0,61
90	0,2	222	0,26
89	0,979	221	0,14
88	0,371	220	0,062
87	0,278	219	0,070
86	0,275	218	0,19
85	0,535	217	0,11
84	0,347	216	0,22
83	0,613	215	0,17
82	0,356	214	0,14
81	0,324	213	0,15
80	0,318	212	0,16
77	0,197	211	0,38
76	0,841	210	0,12
75	0,349	208	0,38
74	0,129	207	0,18
73	0,137	206	0,26
72	0,298	205	0,28
70	0,279	204	0,24
69	0,275	203	0,21
68	0,298	202	0,53
67	0,114	200	0,22
66	0,191	199	0,31
65		198	0,39

64	0,271	197	0,26
63	0,41	195	0,19
62	0,224	194	0,10
61	0,0526	193	0,28
59	0,141	192	0,24
58	0,0349	191	0,74
57	1,4	190	0,29
56	0,226	189	0,49
55	0,143	188	0,14
54	0,125	187	0,15
53	0,195	186	0,48
52	0,0759	185	0,23
51	0,267	184	0,35
49	0,163	183	0,33
48		182	0,12
47	0,0741	181	0,19
46	0,293	180	0,26
45	0,195	179	0,13
43	0,133	178	0,24
41		177	0,34
40	1,75	176	0,26
38		175	0,099
37	0,15	174	0,074
36		173	0,11
35	0,431	172	0,11
33	0,442	171	0,26
32		170	0,11
31	0,476	169	0,038
30	0,233	167	0,19
29		166	0,24
28	0,557	165	0,38

27		163	0,62
26		162	0,19
25		161	0,41
24		160	0,078
23		159	0,37
21		158	0,36
20		157	0,29
19		156	0,14
18		155	0,33
17		154	0,070
16		153	0,087
15		152	0,13
14		151	0,12
13		150	0,14
12	0,703	149	0,066
11		148	0,34
7		147	0,31
5		146	0,27
4		145	0,080
3		144	0,070
2		143	0,070
1		142	0,12
		141	0,38
		138	0,040
		137	0,080
		136	0,070
		135	0,10
		132	0,063
		131	0,091
		130	0,069
		128	0,073

May 2005 - dry season (Nov-April)		Nov 2005 - wet season (May-Oct)	
Average	0,48	Average	0,29
SD	0,61	SD	0,32
mean depth 20 cm		mean depth 60 cm	

Table S12d. MeHg photodegradation (ng m^{-2}) calculated monthly for Florida Everglades by the dark and clear lake exponential spectral models, eqs. (13) and (16), respectively. Depth-integrated annual rates of MeHg photodegradation were calculated by eqs. (20) and (21). Dry season average MeHg concentrations (measured in May 2005, Table S10c) were adopted for the period May-Oct and wet season average MeHg concentrations (measured in Nov 2005, Table S12c) we adoped for the period Nov-Apr. Solar irradiance was calculated monthly for Florida Everglades (average 2006-2015) and spectral absorbance data from Shark lake, Monument, and lake 160 were used as input to the model. Our modelling result of 1 514 ng m^{-2} y^{-1} can be compared with the previously reported rate of MeHg photodegradation (1 240 ng m^{-2} y^{-1}), as calculated by an incidence irradiance model.[31] In both studies the MeHg concentrations in Table S12c were used, while spectral CDOM absorbance in Li et al.[20] was determined from relationships with DOC in Canadian lakes.[43]

	Dry_dark lake model (ng m^{-2})			Dry_clear lake model (ng m^{-2})			Wet_dark lake model (ng m^{-2})			Wet_clear lake model (ng m^{-2})		
	Shark_lake	Monument	Lake_160	Shark_lake	Monument	Lake_160	Shark_lake	Monument	Lake_160	Shark_lake	Monument	Lake_160
Jan	107	79	132	112	76	140	66	48	81	69	47	88
Feb	125	92	151	129	88	159	76	56	93	80	54	99
Mar	169	124	209	174	118	220	103	75	128	107	72	137
Apr	187	138	230	191	131	241	114	84	141	119	80	151
May	197	149	248	202	141	259	120	90	152	125	86	162
Jun	179	135	221	183	127	231	109	82	136	113	78	144
Jul	182	139	233	186	131	244	111	84	143	115	80	152
Aug	173	127	220	177	119	230	106	77	135	109	73	143
Sep	155	113	190	158	107	199	94	69	117	98	65	124
Oct	143	105	174	148	100	183	87	63	107	91	61	114
Nov	116	85	142	121	82	151	71	51	87	75	50	94
Dec	103	75	127	107	72	135	63	45	78	67	44	85
Sum dry	1030	767	1287	1054	724	1346						
Sum wet							492	359	608	517	348	654
Sum year	1521	1126	1895	1571	1072	2000						

	all year	all year	dry season	dry season	wet season	wet season
	Dark lake model	Clear lake model	Dark lake model	Clear lake model	Dark lake model	Clear lake model
Average	1514	1548	1028	1042	486	506
SD	385	465	260	311	124	153

REFERENCES

1. Jankowski, J. J., D. J. Kieber, and K. Mopper. Nitrate and nitrite ultraviolet actinometers. *Photochem. Photobiol.* **1999**, 70: 319-328.

2. Jankowski, J. J., D. J. Kieber, K. Mopper, and P. J. Neale. Development and intercalibration of ultraviolet solar actinometers. *Photochem. Photobiol.* **2000**, 71: 431-440.

3. Koehler, B., Powers, L.C., Cory, R.M, Einarsdóttir, K. Gu, Y., Tranvik, L.J., Vähätalo, A.V., Ward, C.P., Miller, W.L. Inter-laboratory differences in the apparent quantum yield for the photochemical production of dissolved inorganic carbon in inland waters and implications for photochemical rate modeling. *Limnology and Oceanography: Methods*. **2022**, 20(6), 320-337.

4. Snell, J. P., Stewart, I. I., Sturgeon, R. E., & Frech, W. Species-specific isotope dilution calibration for determination of mercury species by gas chromatography coupled to inductively coupled plasma-or furnace atomisation plasma ionisation-mass spectrometry. *Journal of Analytical Atomic Spectrometry.* **2000**, 15(12), 1540-1545.

5. Lambertsson, L., & Björn, E. Validation of a simplified field-adapted procedure for routine determinations of methyl mercury at trace levels in natural water samples using species-specific isotope dilution mass spectrometry. *Analytical and bioanalytical chemistry*. **2004**, 380, 871-875.

6. Hu, C., Muller-Karger, F.E., and Zepp, R.G. Absorbance, absorption coefficient, and apparent quantum yield: a comment on common ambiguity in the use of these optical concepts. *Limnol. Oceanogr*. **2002**, *47*, 1261-1267.

7. Koehler, B., Landelius, T., Weyhenmeyer, G.A., Machida, N., and Tranvik, J.L. Sunlight-induced carbon dioxide emissions from inland waters. *Global Biogeochem. Cycles* **2014**. 28, 696-711.

8. Emde, C., R. Buras-Schnell, A. Kylling, B. Mayer, J. Gasteiger, U. Hamann, J. Kylling, B. Richter, C. Pause, T. Dowling, and L. Bugliaro. The libRadtran software package for radiative transfer calculations (version 2.0.1). GMD. **2016**, 9(5):1647-1672.

9. Mayer, B., and A. Kylling. Technical note: The libRadtran software package for radiative transfer calculations—Description and examples of use, *Atmos. Chem. Phys*. **2005**, 5, 1855–1877.

10. Belward, A. and Loveland, T. The DIS 1-km land cover data set, GLOBAL CHANGE, The IGBP Newsletter. **1996**, 27.

11. Dee, D. P., Uppala, S. M., Simmons, A. J., Berrisford, P., Poli, P., Kobayashi, S., Andrae, U., Balmaseda, M. A., Balsamo, G., Bauer, P., Bechtold, P., Beljaars, A. C. M., van de Berg, L., Bid- lot, J., Bormann, N., Delsol, C., Dragani, R., Fuentes, M., Geer, A. J., Heimberger, L., Healy, S. B., Hersbach, H., Holm, E. V., Isaksen, L., Kallberg, P., Koehler, M., Matricardi, M., McNally, A. P., Monge-Sanz, B. M., Morcrette, J.-J., Park, B.-K., Peubey, C., de Rosnay, P., Tavolato, C., Thepaut, J.-N., and Vitart, F. The ERA-Interim reanalysis: configuration and performance of the data assimilation system, Q. *J. Roy. Meteorol. Soc*. **2011**, 137, 553–597, doi:10.1002/qj.828.

12. Lindfors, A., Heikkilä, A., Kaurola, J., Koskela, T., & Lakkala, K. Reconstruction of solar spectral surface UV irradiances using radiative transfer simulations. *Photochem. Photobiol.* **2009***,* 85, 1233–1239.

13. Arola, A., Kazadzis, S., Lindfors, A. et al. A new approach to correct for absorbing aerosols in OMI UV, *Geophys. Res. Lett.*, **2009**, 36, 22 805, doi:10.1029/2009GL041137.

14. Tanskanen, A., Krotkov, N., Herman, J. R., and Arola, A. Surface ultraviolet irradiance from OMI, *IEEE Trans. Geo. Rem. Sens.* **2006**, 44, 1267–1271.

15. Kinne, S., O'Donnel, D., Stier, P., Kloster, S., Zhang, K., Schmidt, H., Rast, S., Giorgetta, M., Eck, T. F., and Stevens, B. MAC-v1: A new global aerosol climatology for climate studies. *J. Adv. Model. Earth Sys.* **2013,** 5, 704–740.

16. Fichot, C.G., and Miller, W.L. An approach to quantify depth-resolved marine photochemical fluxes using remote sensing: Application to carbon monoxide (CO) photoproduction. *Remote Sens. Environ.* **2010**, 114, 1363-1377.

17. Quan, X., and Fry, E.E. Empirical equation for the index of refraction of seawater. *Appl. Opt.* **1995.** 34, 3477-3480.

18. Hammerschmidt, C. R. and Fitzgerald, W. F. Photodemethylation of methylmercury in an Arctic Alaskan lake. *Environ. Sci. Technol.* **2006,** *40*, (4), 1212-1216.

19. Lehnherr, I.; Louis, V. L. S. Importance of Ultraviolet Radiation in the Photodemethylation of Methylmercury in Freshwater Ecosystems. *Environ. Sci.Technol.* **2009,** *43*, (15), 5692-5698.

20. Li, Y., Mao, Y., Liu, G., Tchiev, G., Roelant, D., Feng, X., and Cai, Y. Degradation of methylmercury and its effects on mercury distribution and cycling in the Florida Everglades. *Environ. Sci. Technol.* **2010,** *44*, 6661-6666.

21. Fernández-Gómez, C., Drott, A., Björn, E., Díez, S., Bayona, J.M, Tesfalidet, S., Lindfors, A. and Skyllberg, U. Towards universal wavelength-specific methyl mercury photodegradation rate constants in humic waters – exemplified by a boreal lake-wetland gradient. *Environ. Sci. Technol.* **2013**, 47, 6279-6287.

22. Poste, A.E., Vetteberg Braaten, H.F., de Wit, H.A., Sorensen, K., and Larssen, T. Effects of photodemethylation on the methylmercury budget of boreal Norwegian lakes. *Environ. Toxicol. Chem.* **2015**, *34*, 1213-1223.

23. Braaten, H. F. V.; de Wit, H. A.; Fjeld, E.; Rognerud, S.; Lydersen, E.; Larssen, T. Environmental Factors Influencing Mercury Speciation in Subarctic and Boreal Lakes. *Sci. Total Environ.* **2014**, 476-477, 336–345.

24. Bravo, A. G.; Bouchet, S.; Tolu, J.; Björn, E.; Mateos-Rivera, A.; Bertilsson, S. Molecular Composition of Organic Matter Controls Methylmercury Formation in Boreal Lakes. *Nat. Commun.* **2017**, *8*, 14255

25. Isidorova, A.; Bravo, A. G.; Riise, G.; Bouchet, S.; Björn, E.; Sobek, S. The Effect of Lake Browning and Respiration Mode on the Burial and Fate of Carbon and Mercury in the Sediment of Two Boreal Lakes. *J. Geophys. Res. Biogeosciences* **2016**, *121* (1).

26 Matilainen, T., and Verta, M. Mercury methylation and demethylation in aerobic surface waters. *Can. J. Fish Aquat. Sci.* **1995**, 52 (8), 1597-1608.

27. Økelsrud, A., Lydersen, E., & Fjeld, E. Biomagnification of mercury and selenium in two lakes in southern Norway. *Sci, Total Environ.* **2016**, 566, 596-607.

28. Rask, M., Verta, M., Korhonen, M., Salo, S., Forsius, M., Arvola, L. Jones, R. and Kiljunen, M. Does lake thermocline depth affect methyl mercury concentrations in fish? *Biogeochem.* **2010**. 101, 311-322.

29. Regnell, O., Ewald, G., & Lord, E. Factors controlling temporal variation in methyl mercury levels in sediment and water in a seasonally stratified lake. *Limnol. Oceanogr.* **1997**. *42*(8), 1784-1795.

30. Selvendiran, P., Driscoll, C. T., Montesdeoca, M. R., Choi, H. D., & Holsen, T. M. Mercury dynamics and transport in two Adirondack lakes. *Limnol. Oceanogr.* **2009**. *54*(2), 413-427.

31. Watras, C. J.; Morrison, K. a.; Host, J. S.; Bloom, N. S. Concentration of Mercury Species in Relationship to Other Site-Specific Factors in the Surface Waters of Northern Wisconsin Lakes. *Limnol. Oceanogr.* **1995**, *40* (3), 556–565.

32. Watras CJ, Back RC, Halvorsen S, Hudson RJ, Morrison KA, Wente SP. Bioaccumulation of mercury in pelagic freshwater food webs. *Sci. Total Environ*. **1998**. 219, 183-208.

33. Watras, C. J., Morrison, K. A., Kent, A., Price, N., Regnell, O., Eckley, C. and Hubacher, T. Sources of methylmercury to a wetland-dominated lake in northern Wisconsin. *Environ. Sci. Technol.* **2005**. *39*(13), 4747-4758.

34. Weishaar, J.L., Aiken, G.R., Bergamaschi, B.A., Fram, M.S., Fujii, R., and Mopper, K. Evaluation of specific ultraviolet absorbance as an indicator of the chemical composition and reactivity of dissolved organic carbon. *Environ. Sci. Technol.* **200**3, 37, 4702-4708.

35. The 2009 Swedish National Lake Inventory by the Swedish University of Agricultural Sciences (http://info1.ma.slu.se/ri/www_ri.acgi$Project?ID=2009KS).

36. Sobek, S., J. Nisell, and J. Fölster. Predicting the depth and volume of lakes from map-derived parameters, *Inland Wat.* **2011**, 1(3), 177–184.

37. Weyhenmeyer, G. A., M. Meili, and D. M. Livingstone.Nonlinear temperature response of lake ice breakup, *Geophys. Res. Lett*. **2004**, 31, L07203, doi:10.1029/2004GL019530.

38. Sellers, P.; Kelly, C. A.; Rudd, J. W. M. Fluxes of methylmercury to the water column of a drainage lake: The relative importance of internal and external sources. *Limnol. Oceanogr.* **2001,** *46*, (3), 623-631.

39. Hines, N. A.; Brezonik, P. L. Mercury inputs and outputs at a small lake in northern Minnesota. *Biogeochemistry* **2007,** *84*, (3), 265-284.

40. Lehnherr, I.; Louis, V. L. S. Importance of Ultraviolet Radiation in the Photodemethylation of Methylmercury in Freshwater Ecosystems. *Environ. Sci.Technol.* **2009,** *43*, (15), 5692-5698.

41. Cory, R.M., Ward, C.P., Crump, B.C, and Kling, G.W. Sunlight controls water column processing of carbon in arctic fresh waters. *Science* **2014**, 345, 925-928.

42. Liu, G., Cai, Y., Kalla, P., Scheidt, D., Richards, J., Scinto, L.J., Gaiser, E., and Appleby, C. Mercury mass budget estimates and cycling seasonality in the Florida Everglades. *Environ. Sci. Technol.* **2008,** *42*, 1954—1960.

43. Scully, N.M., and Lean, D.R.S. The attenuation of ultraviolet radiation in temperate lakes. *Ergeb. Limnol.* **1994**, 43, 133-144.
